# Vitamin C deficiency can lead to pulmonary hypertension: a systematic review of case reports

**DOI:** 10.1186/s12890-024-02941-x

**Published:** 2024-03-19

**Authors:** Harri Hemilä, Angelique M.E. de Man

**Affiliations:** 1 Department of Public Health, University of Helsinki, POB 41, Helsinki, FI-00014 Finland; 2 Department of Intensive Care Medicine, Amsterdam University Medical Centers, location VUmc, Amsterdam, The Netherlands

**Keywords:** Antioxidants, Ascorbic acid, Case report, Heart failure, Oxidative stress, Pulmonary hypertension, Pulmonary vascular resistance, Scurvy, Systematic review

## Abstract

**Background:**

In the early literature, unintentional vitamin C deficiency in humans was associated with heart failure. Experimental vitamin C deficiency in guinea pigs caused enlargement of the heart. The purpose of this study was to collect and analyze case reports on vitamin C and pulmonary hypertension.

**Methods:**

We searched Pubmed and Scopus for case studies in which vitamin C deficiency was considered to be the cause of pulmonary hypertension. We selected reports in which pulmonary hypertension was diagnosed by echocardiography or catheterization, for any age, sex, or dosage of vitamin C. We extracted quantitative data for our analysis. We used the mean pulmonary artery pressure (mPAP) as the outcome of primary interest.

**Results:**

We identified 32 case reports, 21 of which were published in the last 5 years. Dyspnea was reported in 69%, edema in 53% and fatigue in 28% of the patients. Vitamin C plasma levels, measured in 27 cases, were undetectable in 24 and very low in 3 cases. Diet was poor in 30 cases and 17 cases had neuropsychiatric disorders. Right ventricular enlargement was reported in 24 cases. During periods of vitamin C deficiency, the median mPAP was 48 mmHg (range 29–77 mmHg; *N* = 28). After the start of vitamin C administration, the median mPAP was 20 mmHg (range 12–33 mmHg; *N* = 18). For the latter 18 cases, mPAP was 2.4-fold (median) higher during vitamin C deficiency. Pulmonary vascular resistance (PVR) during vitamin C deficiency was reported for 9 cases, ranging from 4.1 to 41 Wood units. PVR was 9-fold (median; *N* = 5) higher during vitamin C deficiency than during vitamin C administration. In 8 cases, there was direct evidence that the cases were pulmonary artery hypertension (PAH). Probably the majority of the remaining cases were also PAH.

**Conclusions:**

The cases analyzed in our study indicate that pulmonary hypertension can be one explanation for the reported heart failure of scurvy patients in the early literature. It would seem sensible to measure plasma vitamin C levels of patients with PH and examine the effects of vitamin C administration.

**Supplementary Information:**

The online version contains supplementary material available at 10.1186/s12890-024-02941-x.

## Background

In the early literature, vitamin C deficiency was associated with exertional dyspnea. James Lind wrote in his 18th century monograph that “the case of scorbutic patients is somewhat singular, that though when at rest they find themselves quite well; yet, upon the least exercise, they are subject to panting and breathlessness” [1, 2]. Lind proposed that the cause of exertional dyspnea might be in the right ventricle: “upon using exercise, the velocity of blood is accelerated through lungs, and much greater quantity, which when at rest, was almost stagnating in the veins, is at once returned into the right cavities of the heart, and from thence into the lungs; the weakened vessels of the lungs not being able so quickly to transmit so great a quantity, causes a laborious breathing and panting” [1, 2].

In another major monograph on scurvy two centuries later, Alfred Hess described that dilatation in the right ventricle was common in patients with scurvy [2, 3]. Erdheim reported right heart enlargement in 21 out of 31 autopsies of infantile scurvy, with a higher degree of enlargement and hypertrophy of both ventricles in the more severe cases [4]. Right heart dilatation was also described in other reports [5–14]. Furthermore, some early reports proposed vitamin C for treating heart failure (HF) [15–17].

Animal studies indicated that vitamin C has effects on the function and structure of the heart and arteries. Experimental vitamin C deficiency in guinea pigs led to enlargement of the heart and degenerative changes in heart valves and myocardium [18–29]. Vitamin C deficiency also caused degenerative changes in the lungs [20–24], such as narrowing of arterioles. In mice, vitamin C deficiency led to alterations in the aorta wall, such as the disruption of elastic laminae and smooth muscle cell proliferation [30]. Vitamin C prevented pulmonary hypertension in broilers, which are especially prone to it due to their high metabolic rates and oxygen requirements [31, 32].

In clinical context, pulmonary hypertension (PH) is defined as mean pulmonary arterial pressure (PAP) over 20 mmHg [33–36]. Elevated PH increases the risk of right heart failure (RHF) and mortality [37, 38]. PH is categorized into 5 groups. Group 1 encompasses pulmonary artery hypertension (PAH), one cause of which is endothelial dysfunction [33, 34, 39, 40]. Pathophysiology of PAH involves impaired production of vasodilators such as nitric oxide (NO) and prostacyclin, along with the over-expression of vasoconstrictors such as thromboxane and endothelin-1. Most current drugs for PAH are focused on these pathways [33, 34, 36]. Furthermore, elevated levels of hypoxia-inducible factor 1α (HIF-1α) seem to play an important role in PAH, such that the accumulation of HIF-1α can lead to pulmonary vasoconstriction and smooth muscle cell proliferation [41]. PAH is also associated with epigenetic changes [42]. Carnitine participates in energy metabolism and has been proposed for treating PAH [43]. Exercise intolerance in PAH may be caused by the degeneration of skeletal muscles [44].

Vitamin C may influence PAH by several mechanisms. Physiological studies have demonstrated that vitamin C has effects on endothelial function in coronary arteries [45–49]. It participates in the synthesis of type IV collagen, which is required for basement membrane formation and endothelial cell adhesion [50]. Vitamin C participates in the synthesis of NO [50–52], and may increase prostacyclin levels [53–57]. A controlled trial found decreased thromboxane levels in the vitamin C group [58], and another study reported that vitamin C prevented the effects of endothelin-1 [59]. Vitamin C participates in the hydroxylation of specific proline residues of HIF-1α and thereby increases its rate of degradation, leading to decreased HIF-1α levels [60, 61]. Vitamin C participates in the demethylation of DNA and histones and influences over 1000 genes through epigenetic changes [62, 63]. Vitamin C participates in the biosynthesis of carnitine [64, 65], and carnitine administration extended the life span of scorbutic guinea pigs [66]. Finally, in the guinea pig, vitamin C deficiency leads to the degeneration of skeletal muscles [18–21, 67], and in humans, vitamin C administration increased resistance of skeletal muscle to fatigue in COPD patients [68].

Over the last decade, an increasing number of case reports have suggested vitamin C deficiency as a cause of PH. The purpose of our study was to analyze the findings of such reports, to evaluate the timing and effect of vitamin C, and to classify the published PH cases.

## Methods

### Search for case reports

We included case reports in which vitamin C deficiency was considered the cause of PH. We restricted our analysis to reports in which PH was diagnosed by echocardiography or catheterization. Catheterization is the gold standard method to diagnose PH [33]; however, it is not widely available, and it is invasive. The correlation between the PAP from echocardiography and catheterization is high [69–71]. When both methods were used, we selected the outcomes from catheterization.

We did not restrict our report selection based on age or sex, or dose and route of vitamin C administration. Our searches are described in Supplement 1, which includes data about the patients, their signs and symptoms, findings, diet before diagnosis, vitamin C treatment, and the effects of vitamin C. Supplement 2 (spreadsheet) includes numerical data extracted from the case reports, and calculations. We were able to contact several authors for further details.

### Primary outcome

We used the mean PAP (mPAP) as the outcome of primary interest. PH is diagnosed when mPAP > 20 mmHg [33].

### Statistical methods

Many case reports did not publish mPAP. Some reported systolic PAP (sPAP) and we calculated an estimate of the mPAP using the Chemla formula: mPAP = 0.61×sPAP + 2 mmHg [72, 73]. Some further case reports published maximal tricuspid regurgitation velocity (TRV). Assuming right atrial pressure (RAP) = 10 mmHg [71, 74], we calculated an estimate of sPAP using the modified Bernoulli Eq. [69, 71, 74, 75]: sPAP = 4×TRV^2^ + RAP; thereafter we calculated the mPAP as above.

In our main analyses we include all cases, ignoring other vitamins, minerals, and drugs; this is discussed further in the Results section.

## Results

### Description of the included case reports

We identified 32 case reports which suggested that vitamin C deficiency was the cause of PH [76–105] (Supplements 1 and 2). The oldest reports were published in 1996 [92] and 2003 [99], while 21 have been published since 2019 (Table 1). Over half were from the USA and the remaining cases originated from several other countries.

**Table 1:** Characteristics of the patients in the case reports and the findings

Study [ref]	Country	Age (y)	Sex	Neuro-psychiatric disorders	mPAP		Ratio ( mPAP pre/post )	Benefit on cardiac outcomes (days)^a^
					diagnosis (mmHg)	repeat ( mmHg)		
Vitamin C alone (16 cases)^b^								
Abbas (2016) [76]	USA	50	F	Anxiety	^c^			28
Abe (2021) [77]	USA	7	M	ASD	46	22	2.1	38
Azar (2023) [79]	USA	35	F	Anxiety	41			21
Conte (2021) [81]	USA	48	F	Anxiety	35			
Ferreira (2020) [84]	Brazil	51	M	Paranoid	39			
Ghulam Ali (2018) [87]	Italy	66	M		61	19	3.2	14
Gilmore (2021) [88]	USA	22	M	ASD	32	12	2.8	42
Kurnick (2023) [91]	USA	25	F		45	24	2.0	
Mehta (1996) [92]	USA	40	F	Anorexia	36			19
Mertens (2011) [93]	USA	74	F	Food delusions	51			150
Nagamatsu (2009) [94]	USA	73	F		48	23	2.1	90
Petersen (2019) [97]	USA	5	M	ASD	^c^			14
Shah (2021) [101]	USA	35	F	Anxiety	54	19	2.9	14
Singh (2017) [102]	USA	48	F		41			30
Tan (2021) [103]	Malaysia	7	M	ASD	48	14	3.4	10
Valencia (2022) [105]	USA	19	M		76	33	2.3	
Vitamin C with other vitamins or minerals (15 cases)								
Benhamed (2019) [80]	France	40	M		63	19	3.3	2
Dean (2018) [82]	USA	6	M		43	14	3.2	2
Duvall (2013) [83]	USA	9	M	ASD	45			9
Frank (2019) [85]	USA	17	M		51	22	2.3	30
Gayen (2020) [86]	USA	60	M		41	20	2.1	150
Ichiyanagi (2019) [89]	Japan	3	M	ASD	77	19	4.1	1
Kupari (2012) [90]	Finland	40	F		48	15	3.2	7
Nariai (2022) [95]	Japan	2	F		54	21	2.5	2
Penn (2019) [96]	USA	48	F		41	26	1.6	42
Quinn (2022) [98]	USA	6	M	ASD	42	21	2.0	9
Ratanachu (2003a) [99]	Thailand	2	M		^c^			7
Ratanachu (2003b) [99]	Thailand	3	M		^c^			7
Sakamornchai (2022a) [100]	Thailand	6	M	ASD	57			60
Sakamornchai (2022b) [100]	Thailand	5	M	ASD	57			1
Ueki (2022) [104]	Japan	11	M	ASD	29	23	1.2	105
Vitamin C was not administered (1 case)								
Azar (2019) [78]	USA	73	F		50			Died

This table summarizes the data collected in Supplements 1 and 2

*ASD *Autism spectrum disorder

^a^Many studies reported only a late repeat echocardiograph. Late benefit does not indicate that the effect of vitamin C necessarily was slow. For example, Mertens (2011) [93] stated that a repeat echocardiography was carried out after 5 months, but there is no information about the cardiac status before the 5-month time point. See a survival curve of the effect of vitamin C on cardiac outcomes in Fig. S2

^b^Vitamin C alone indicates that no other vitamins or minerals were administered. Many patients in this group were administered drugs for PAH. However, no PAH drugs were administered for the patients reported in Abbas (2016) [76], Abe (2021) [77], Ferreira (2020) [84], Gilmore (2021) [88], Mehta (1996) [92], and Shah (2021) [101]

**Fig. 1:**
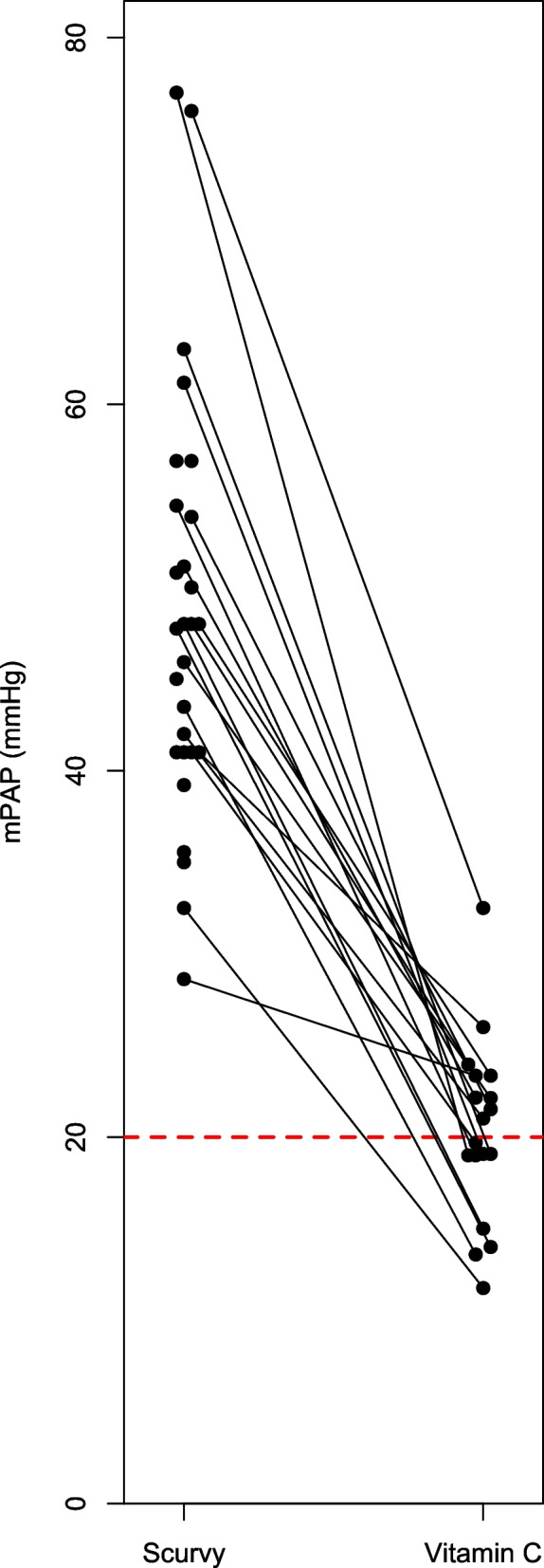
Mean pulmonary artery pressure (mPAP) during scurvy and after vitamin C administration. mPAP was available at baseline for 28 patients, but 1 patient died before vitamin C was given [78]. The median mPAP level was 47 mmHg during scurvy. mPAP level after vitamin C administration was available for 18 cases, with the median mPAP 20 mmHg. The lines indicate the patients with both values. The mPAP level during scurvy was 2.4 fold (median; range 1.2–4.1) higher than the level during vitamin C administration. The red dash line indicates 20 mmHg, which is the limit for concluding PH [33]

^c^Four case reports stated that PH was demonstrated, but no mPAP estimate is available. They are not shown in Fig. 1

Twelve cases concerned children aged 1–10 years, 10 cases were in patients aged 11–44, and 10 were about patients aged 45–74 years. The sexes were distributed equally (Table 1). Half of the cases had a neuropsychiatric background such as autism, anxiety, anorexia, or paranoidism. Half of the children had autistic spectrum disorder (ASD).

The health condition that led to the patient being hospitalized lasted for over 3 months in 12 cases, 1–2 months in 7 cases, and 3 weeks or less in 7 cases.

Dyspnea was reported in 69%, edema in 53% and fatigue in 28% of the patients (Table 2). Chest pain was reported in 4 patients. Common symptoms included petechiae and ecchymoses, and pains in muscles and joints. Pathological changes in the gums were reported only in 56% of the cases. Of the remaining cases, 7 were thorough reports and we consider it likely that gum changes would have been reported if observed [76, 87, 90, 91, 98, 100, 103]. Corkscrew hairs are pathognomonic to scurvy but were reported for only 6 patients. Possibly physicians are more consistent in reporting dyspnea in patients with PH than on searching and reporting corkscrew hairs. Thus, the percentages should not be considered as the definite assessment of prevalence.

**Table 2:** Distribution of symptoms and signs in patients with scurvy-induced pulmonary hypertension

Symptom	Cases
Dyspnea	22
Edema	17
Fatigue	9
Chest pain	4
Gum pathology	18
Petechiae and ecchymoses	17
Pains in muscles and joints	19
Corkscrew hairs	6

The frequencies should not be compared as definite evidence of different prevalence of the symptoms, since physicians may record and report, for example, dyspnea more consistently than corkscrew hairs. This table is based on Supplements 1 and 2

For 9 of the 12 children, the reported heart rates and respiratory rates were above the reference range [106] (Fig. S1 in Supplement 1). Only 1 child had levels within the reference range [104]. Over all ages, low systolic blood pressure < 100 mmHg was reported in 10 out of 22 cases (Fig. S1). Of the 20 cases that reported the level of blood O_2_ saturation, 6 reported low saturation (< 90% or “hypoxia”), and oxygen administration was reported in 7 cases. B-type natriuretic peptide (BNP or NT-proBNP) was reported in 12 cases, and all were elevated. The majority of cases that published the hemoglobin level reported anemia with Hb < 100 (11/16 cases).

For 30 cases diet was reported to be poor. The patients consumed a narrow selection of foods, avoiding fruit and vegetables. Two cases did not comment on diet, one was a brief report [88], and another had Crohn’s disease and underwent liver transplantation [105].

Vitamin C plasma level was measured in 27 cases. In 24 cases the level was undetectable with a local assay. The level was measurable but very low in 3 cases: 3 µM [84], 6 µM [76], and 11 µM [92].

The dose of vitamin C was reported for 19 cases. In 9 cases the dose was between 0.15 and 0.84 g/day, in 8 cases it was 1 g/day, and in 2 cases 2 g/day. At the start of the treatment, vitamin C was administered orally in 13 cases, and intravenously in 11 cases. Vitamin C was administered without other micronutrients in 17 cases, while in 14 cases other vitamins or trace elements were administered together with vitamin C.

### Cardiac findings in the case reports

In 31 cases, echocardiogram was used to diagnose PH, followed by catheterization in 14 cases to confirm the diagnosis and to differentiate between pre- and post-capillary PH. In 1 case the diagnosis was based solely on catheterization. To exclude other diagnoses and to determine the etiology of PH, chest CT (20 cases), ventilation/perfusion scan (7 cases), pulmonary function test (5 cases), and cardiac MRI (1 case) were performed (see Supplements 1 and 2). From ECGs, 7 cases suggested right ventricle (RV) hypertrophy: 5 had right axis deviation and 3 had right bundle branch block.

Of the 32 case reports, 26 reported cardiac features of PH: RV enlargement in 24 cases and septal flattening in 14 cases. Pericardial effusion was reported in 4 cases [79, 90, 99, 103]. The P2 sound is common in PH [33, 107] and was reported in 6 cases.

The mPAP levels were published or could be calculated for 28 cases. At the time of the PH diagnosis, the median mPAP was 48 mmHg (range 29–77 mmHg) (Fig. 1). After vitamin C administration, mPAP was available for 18 cases, with a median of 20 mmHg (range 12–33 mmHg). For the latter 18 cases, mPAP was 2.4 fold (median; range 1.2–4.1) higher during periods of scurvy than during vitamin C administration.

The post-treatment mPAP level and the route of vitamin C administration was available for 16 cases. With oral administration, the median mPAP was 19 mmHg (range 12–26 mmHg; 8 cases), compared with intravenous administration with a median mPAP of 22 mmHg (range 13–33 mmHg; 8 cases).

**Fig. 2:**
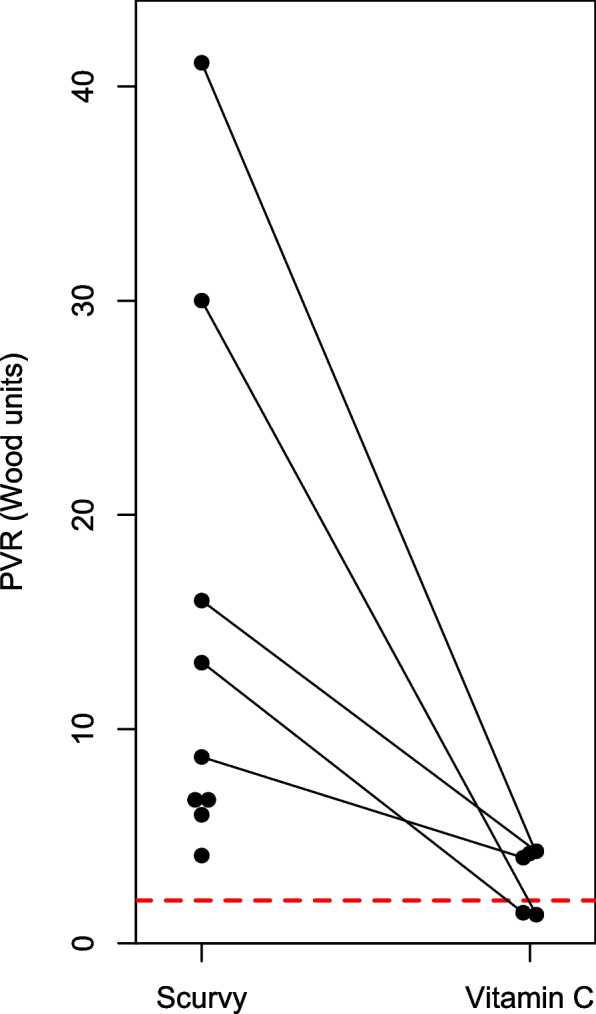
Pulmonary vascular resistance (PVR) during scurvy and after vitamin C administration. PVR was available at the baseline for 9 cases, but 1 case died before vitamin C [78]. Follow-up PVR level was available for 5 cases with the lines showing the paired values. During scurvy, the PVR level was 9 times (median) as high as during vitamin C administration. The red dash line indicates 2 Wood units, which is the limit for concluding PAH [33]. See extracted data in Supplement 2

Nine cases reported pulmonary vascular resistance (PVR) at the time of PH diagnosis, ranging from 4.1 to 41 Wood units (WU); PVR > 2 WU indicates pre-capillary PH [33] (Fig. 2). One of these 9 patients died before vitamin C was administered [78]. Five cases published the PVR level after vitamin C administration. During periods of scurvy PVR was 9-fold (median; range 2.2–22) higher.

Of the 19 patients for whom a time point of observed benefit from vitamin C on symptoms or signs was reported, 12 described that there was substantial benefit within 4 days. In this calculation, we have made the interpretation that “rapidly” [76], “in a few days” [79, 99], and “progressively” [77] indicate within 4 days.

A time point for demonstrated benefit on cardiac outcomes was reported in 28 cases. These time points are not based on regular daily follow-up; instead, repeat echocardiography was usually carried out after a fixed time period such as after 1 week or 1 month depending on local routines. Therefore, the recorded time of improvement in the cardiac outcomes is biased upwards. Nevertheless, it is useful to examine the rate of benefit. In 8 cases cardiac improvement was demonstrated in the first week, and in 7 other cases in the second week (Fig. S2). Thus, benefit in cardiac outcomes within 2 weeks was shown in half of the patients (15/28).

**Fig. 3:**
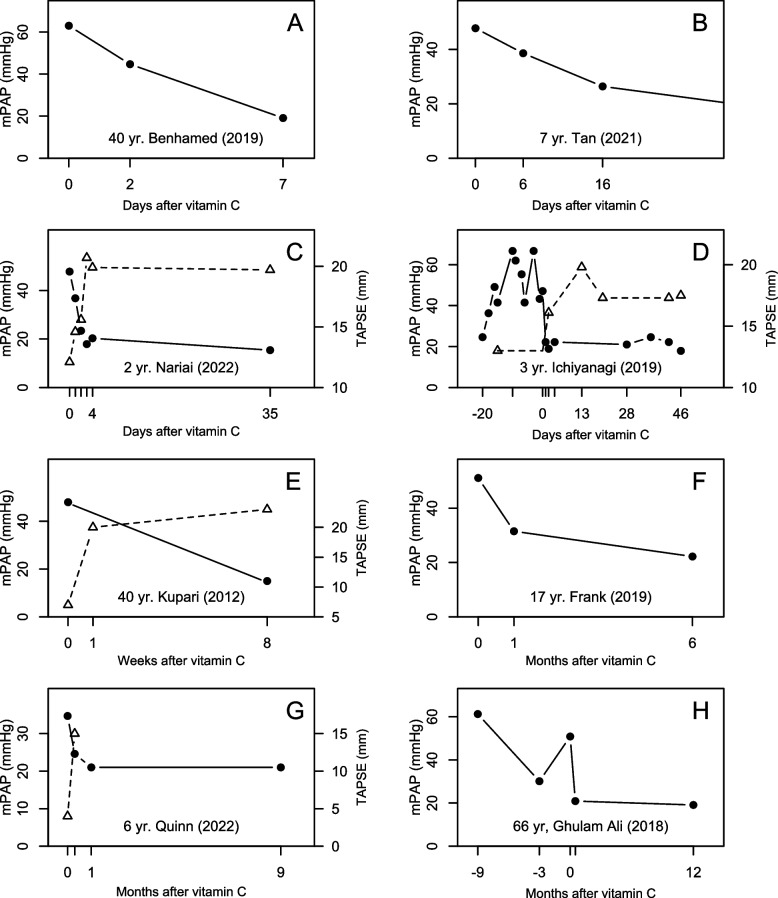
Evolution of mPAP and TAPSE over time. mPAP is shown with filled circles on the left-hand side, and the TAPSE with open triangles on the right-hand side. Each point indicates one observation, and the lines are added to help visualize the change over time. Normal TAPSE ≥ 18 mm [33]. Note that the time scale of the horizontal axis is not constant. Time points ≤ 0 indicate the period during scurvy. Vitamin C administration was started soon after the time point 0. Data are for: (**A**) a 40-year-old male in France [80], (**B**) a 7-year-old boy in Malaysia [103], (**C**) a 2-year-old girl in Japan [95], (**D**) a 3-year-old boy in Japan [89], (**E**) a 40-year-old female in Finland [90], (**F**) a 17-year-old male in the USA [85], (**G**) a 6-year-old boy in the USA [98], (**H**) a 66-year-old male in Italy [87]. See extraction of data in Supplementary files 1 and 2. TAPSE, tricuspid annular plane systolic excursion

Eight cases reported mPAP for more than 1 repeat examination after the start of vitamin C administration. In most cases there was a substantial decline in the mPAP level within a few days or weeks (Fig. 3).

Tricuspid annular plane systolic excursion (TAPSE) is a measure for RV function, with normal level ≥ 18 mm [33, 108]. In 4 patients, TAPSE rapidly increased to normal levels after vitamin C administration (Fig. 3). In one patient, TAPSE was 9.5 mm during scurvy, but 23 mm 4 months earlier (Fig. S3), but follow-up TAPSE after vitamin C administration was not reported [91].

**Fig. 4:**
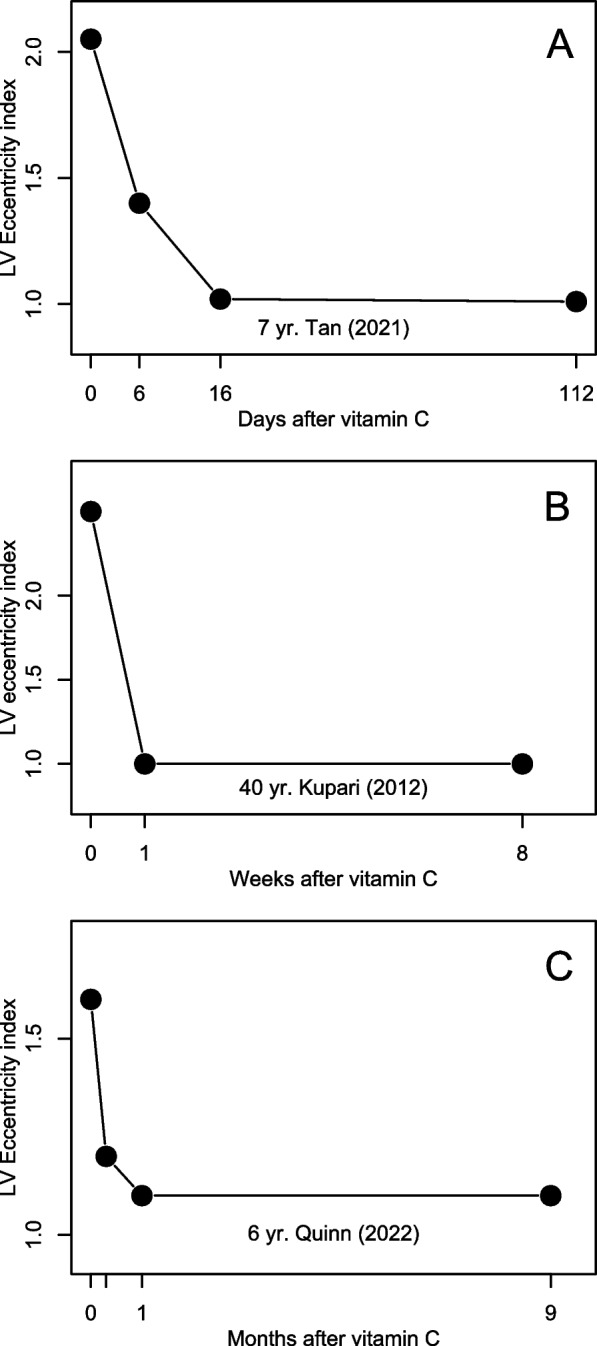
Effect of vitamin C on LV eccentricity index in pulmonary hypertension patients. Normal LV eccentricity index is 1.0 [33, 109]. Each point indicates an observation, and the lines are added to help visualize the change over time. Time point 0 indicates the start of vitamin C administration. Data are from (**A**) Tan [103], (**B**) Kupari [90], and (**C**) Quinn [98]

Normal left ventricles (LV) are quite rounded, and the LV eccentricity index is a measure of RV overload, such that 1.0 indicates normal RV volume and pressure load, whereas RV pressure overload is indicated by an eccentricity index ≥ 1.2 at the end of both diastole and systole [33, 109]. In 3 cases, an elevated LV eccentricity index rapidly decreased after vitamin C administration (Fig. 4).

After the start of vitamin C, the size of the right heart, and the diameter of vena cava inferior substantially decreased in one patient (Fig. S4A [90]), and in another patient, the RV systolic to diastolic duration ratio decreased, and the RV fractional area change increased (Fig. S4B [98]).

### Classification of the included cases

Authors of the reports proposed that 10 cases were PAH. According to current recommendation group 1 PAH classification is based on three criteria: mPAP > 20 mmHg, pulmonary wedge pressure < 15 mmHg and PVR > 2 WU [33]. Only 5 of the 10 proposed PAH cases demonstrated these criteria [78, 79, 82, 86, 96]. However, we noted 3 further cases satisfying the criteria [89–91]. Thereby, 8 of the 32 patients were PAH cases.

Thus, 24 cases were not demonstrated to have PAH. Nevertheless, some or all of them might be cases of PAH, but firm conclusions cannot be drawn in the absence of definitive data. On the other hand, of these remaining 24 cases with unclear PH classification, chronic pulmonary thromboembolism was excluded in 21 cases by CT and/or ventilation-perfusion scans, and lower limb deep vein thrombosis in 4 cases by Doppler ultrasound, thereby excluding group 4 PH [33]. None of these 24 case reports indicated interstitial lung diseases or COPD, making group 3 PH unlikely [33]. In addition, none of the 24 case reports indicated substantial long-term LV dysfunction before the PH episode, and in 23 of the 24 cases echocardiography would have shown LV dysfunction if it existed, thereby excluding group 2 PH [33]. Finally, in most cases the recovery from PH was rapid and such a response to vitamin C is inconsistent with the chronic conditions behind PH groups 2, 3 and 4. Only Valencia et al. reported wedge pressure 30 mmHg and PVR 6.7 WU in a patient after liver transplant [105], probably explained by left-sided heart disease [33].

### Effect of vitamin C on laboratory endpoints

In a few cases, laboratory assays were reported after vitamin C administration. Ueki et al. reported a 80–90% decrease in BNP, CRP and D-dimer levels [104]. Dean et al. reported normalization of the BNP level [82]. Hb level doubled in 2 cases during vitamin C administration [90, 104].

### Specificity of the effects of vitamin C

There are no control patients in our analysis and some of the changes over time may have been caused by factors other than vitamin C. Evidently, even with its potential limitations, hospital food is better than the food eaten at home for most of the included patients, but none of the cases improved just by being taken to hospital.

Hospital stay usually includes a number of medical treatments, but the other treatments did not lead to sufficient improvement in the included cases. Within hospitals, several patients were administered standard PAH drugs such as prostacyclins [90, 96, 102, 105], phosphodiesterase 5 inhibitors [78, 79, 82, 89, 90, 96, 98, 102, 103, 105], endothelin receptor antagonist [87, 103, 105], inhaled NO [82, 98, 103], calcium channel blockers [93, 94, 100], phosphodiesterase 3 inhibitors [82, 91, 95, 105], and nonspecified pulmonary vasodilator [100].

None of them led to substantial clinical improvement. Furthermore, when sildenafil and tadalafil [82, 89, 90, 96, 102], bosentan [87], and nifedipine [93] were discontinued after discharge, the patients remained cured of PH by taking vitamin C alone. Finally, no standard PAH drugs were administered for 13 cases.

Another potential confounding factor is the administration of other vitamins and minerals. Thiamine (vitamin B_1_) deficiency can cause PH and RHF [5, 110–112], however, the level was normal in 9 of the 15 cases for whom plasma thiamine level was measured, and the few low levels were mostly marginally low. Thiamine was administered to 7 cases. Thus, no proportion of clinical improvement in 24 cases can be attributed to thiamine. Vitamin D was administered to 4 cases. A few cases were administered iron or multivitamins. However, 17 cases were administered vitamin C alone (Table 1).

A further approach to estimate the possible contribution of the other vitamins and minerals was to compare the post-treatment mPAP levels in those who received vitamin C alone vs. those who received other vitamins or minerals together with vitamin C. In 9 cases receiving vitamin C alone, the median post-treatment mPAP was 19 mmHg (range 12–33 mmHg). In 9 cases receiving other vitamins or minerals, the median post-treatment mPAP was 21 mmHg (13–26 mmHg).

In 7 cases, no PAH drugs and no other vitamins or minerals were administered and the improvements in those patients gives the most specific evidence that it was vitamin C that caused the decrease in mPAP levels [76, 77, 84, 88, 92, 97, 101].

Vitamin C was undetectable in 24 cases and very low in 3 cases. Evidently, such extremely low levels are rapidly increased with vitamin C administration, which can explain the dramatic and rapid effects on mPAP. In most cases there was a long progressive deterioration in the condition over weeks or months before the hospital assessment. In many cases the improvement in the condition was rapid after vitamin C was started. For example, “after 2 days, his symptoms improved and he was discharged home” [85], “[tricuspid regurgitation peak gradient] dramatically decreased [from 75 mmHg] to … 35 mmHg on day 3” [95], and after vitamin C “by 48 hours, her symptoms had resolved. Repeat right heart catheterization demonstrated … mPAP [from 41 to] 26 mm Hg” and PVR from 13 WU to 1.4 WU [96]. Given the long decline in health, such rapid improvements after starting vitamin C are consistent with the special role of the vitamin.

## Discussion

### Findings of the case reports

We identified 32 case reports that are informative about the effects of vitamin C on PH. One patient died before vitamin C was administered [78]. In the other 31 cases dramatic benefits from vitamin C were reported. In 18 cases with follow-up data, mPAP during scurvy was on average 2.4 times higher than during vitamin C administration (Fig. 1). In 8 cases which reported repeated mPAP measurements, vitamin C was shown to decrease the levels within days or weeks (Fig. 3). In 9 cases, the PVR was substantially elevated, and in 5 of those, vitamin C dramatically decreased the level (Fig. 2).

Low TAPSE (RV dysfunction) is an important predictor for decreased survival in PH [33, 108, 113–115]. Vitamin C rapidly improved TAPSE in 4 cases (Fig. 3).

Decreased survival in PH is associated with elevated LV eccentricity index (RV pressure overload), increased size of RA and RV, decreased RV fractional area change, increased RV systolic-to-diastolic duration ratio, and increased diameter of vena cava inferior [33, 113–120]. These endpoints were normalized rapidly by vitamin C in 4 cases (Fig. 4 and Figs. S3 and S4). Elevated BNP level is also associated with decreased survival [121]: 12 cases had elevated levels during scurvy, but only two reported follow-up levels which were normalized [82, 104].

Case reports such as those included in our analysis may be biased in the assessment of the treatment effects. The placebo effect and concurrent treatments might explain the observed benefits. However, the placebo effect is a concern for subjective outcomes, but less or not at all for objective outcomes [122, 123]. It does not seem plausible that the placebo effect of vitamin C could cause permanent declines in outcomes such as mPAP and PVR. Furthermore, in several RCTs, the mPAP level was not changed in the placebo groups of PH patients consistent with this argument [124–126]. Many of the included cases were administered drugs for treating PAH, and other vitamins and minerals, but it seems highly unlikely that the rapid and permanent benefits could be explained by factors other than vitamin C.

Intravenous vitamin C administration has often been proposed since it can lead to faster normalization and higher levels of plasma vitamin C [127]. In our set of cases, there was no indication of differences between intravenous and oral administration. Given the very low levels of vitamin C in plasma at the time of diagnosis, the route of administration may be a marginal issue. Furthermore, a meta-analysis comparing intravenous vs. oral vitamin C for the length of ICU stay found no difference [128]. Nevertheless, a meta-analysis on vitamin C and the incidence of atrial fibrillation (AF) found differences between oral and intravenous administration [129]. For very ill patients, who are also prone to gastroparesis, and have increased metabolic needs, intravenous vitamin C may be the only option, but oral vitamin C should not be generally underrated.

### Classification of the included cases

PH is a heterogeneous condition, the end result of a variety of underlying disorders. It is currently categorized into 5 groups [33]. Group 1 encompasses patients with PAH, group 2 encompasses those who have PH due to left heart disease, group 3 PH is due to chronic lung diseases such as COPD and pulmonary fibrosis, and group 4 PH is due to chronic pulmonary thromboembolic disease. Group 5 encompasses etiologies outside the 4 other groups.

In 8 cases, there was evidence that the cases were PAH. The classification of the remaining 24 cases is not clear. In controlled trials, vitamin C has influenced left ventricular ejection fraction (LVEF) [130], which indicates that vitamin C can have effects on LV function. Controlled trials have also shown that vitamin C can influence pulmonary function tests [131, 132]. Therefore, it is conceivable that in some situations vitamin C may have an effect on PH groups 2 and 3. However, most of the unclassified PH cases are inconsistent with groups 2 to 5. The patient reported by Valencia et al. is the only one with data indicating a contribution to PH from the LV [105]. Therefore, our conclusion is that the majority of the unclassified cases probably fall into group 1 PAH.

PAH is a severe disease with a 3-year survival rate of about 50% [133], but PAH is rare [33]. On the other hand, a population-based study in the Netherlands found that 8% of people older than 85 years had echocardiographic signs of PH [134]. Even mild PH increases mortality [38]. The potential role of vitamin C on group 2 and 3 PH should be investigated.

### Low vitamin C levels are not rare

Although the evidence from the included case reports indicates strongly that low vitamin C levels can lead to PH, it is important to emphasize that the plasma vitamin C levels were particularly low. Therefore, caution is needed when extrapolating these findings to less extreme vitamin C deficiency.

While the majority of the cases reported unmeasurable plasma vitamin C levels, 2 cases reported plasma vitamin C levels in the range 6–11 µmol/L [76, 92]. Thus, 11 µmol/L might be considered a potential upper limit for the range with an increased risk of PH due to vitamin C deficiency. This level needs to be compared with levels reported in various population groups.

In the NHANES 2003–2004 survey in the USA, median plasma vitamin C level was 54 µmol/L among the population aged over 20 [135]. However, 5% of those examined had vitamin C levels below 8 µmol/L. Furthermore, in some populations plasma vitamin C level is much lower than in the USA. For example, vitamin C plasma level < 11 µmol/L was reported in 25% of low-income males in the UK [136], in 39% of females in Mexico [137], and in 59% of subjects of a survey in India [138]. Finally, in Northern Russia mean vitamin C plasma level was 2.5 µmol/L in 1992 and 5 µmol/L in 2002 [139]. Therefore, if vitamin C level < 11 µmol/L increases the risk of PH, the issue is of concern in many population groups globally. Furthermore, one survey intentionally searched for scurvy symptoms and signs in geriatric patients and concluded that 12% had scurvy [140].

Finally, plasma vitamin C is a poor measure of vitamin C status in the body. Scurvy is usually associated with vitamin C plasma levels below 0.2 mg/dl (11 µmol/L), but 11 µmol/L should not be interpreted as a definitive level below which scurvy symptoms start to appear. For example, Hodges commented in their empirical vitamin C deficiency trial that “a distressing feature is the lack of precision of serum ascorbic acid levels. According to most authorities, deficiency appears after the serum level has fallen below 0.2 mg/100 ml, yet several men in these studies had obvious scurvy at a time when their serum levels were above this value” [141]. Emergence of scurvy symptoms had a much closer correlation with the total body pool of vitamin C than with plasma vitamin C level. Hodges further stated that “… a comparison between plasma levels of ascorbate and pool sizes showed a very poor correlation… it is fair to say that scurvy appeared when the [vitamin C] body pool size fell below 300 mg” [142]. However, it is not feasible to measure total body pool of vitamin C in hospitalized patients.

When the deficiency of vitamin C is considered, often the main focus is on the gums. However, only half of the included cases reported pathological changes in gums. Of the remaining cases, seven were thorough reports such that we would expect mentioning gum pathology if such existed. Accordingly, too much weight should not be put on the presence of gum pathology when considering the possibility of vitamin C deficiency.

### Ethical issues around not giving vitamin C to patients with severe vitamin C deficiency

RCTs are strongly encouraged to validly test whether a treatment is effective or not. However, there are ethical concerns regarding withholding vitamin C from patients who suffer from vitamin C deficiency. In old times, vitamin C deficiency led to the end of many lives [1–3, 143, 144]. The harms of vitamin C deprivation have not vanished. One of the cases included in our analysis died of scurvy, with the pathophysiological explanation being cardiogenic shock [78]. Four other modern case reports of vitamin C deficiency described patients who died [145–148]. Vitamin C deficiency has caused ECG changes, which also discourages randomizing half of deficient patients to a placebo group [149–153]. The Helsinki declaration states that “while the primary purpose of medical research is to generate new knowledge, this goal can never take precedence over the rights and interests of individual research subjects” [154]. It does not seem ethically acceptable to randomize any patients with vitamin C deficiency to a group that is not given the vitamin.

The LOVIT trial examined the effect of 4-day intravenous vitamin C administration on sepsis patients, and unexpectedly mortality was found to be elevated in the vitamin C group [155]. However, a secondary analysis showed that the increased mortality did not occur during vitamin C administration, but immediately after the abrupt termination of the vitamin [156]. A recent vitamin C for critically-ill COVID-19 patients trial observed similar harm from the abrupt termination of the 4-day vitamin C administration [157, 158]. Rapid termination of vitamin C can lead to very low plasma levels through the rebound effect [156]. It even seems possible that the extra deaths after the sudden termination of vitamin C in the LOVIT trial might have been due to RHF since vitamin C deficiency increases the risk of RHF [1–11], and RHF is common in sepsis patients [159, 160].

So, there are serious ethical issues if vitamin C is not administered to patients with vitamin C deficiency. Furthermore, when the size of the effect is large relative to the expected prognosis, RCTs are not necessary for drawing definite conclusions [161]. Thus, the findings described in our study should not be dismissed with a requirement that an RCT should be carried out to test whether maintaining scurvy maintains elevated mPAP levels.

### Other cardiac effects of vitamin C

Our study focuses on PH, but vitamin C appears to have other cardiac effects. Meta-analyses indicate that vitamin C can lower systemic blood pressure [162], increase low levels of LVEF [130], decrease the risk of AF in high-risk populations [129], decrease troponin levels in cardiac stress [163], and improve endothelial function [164]. One RCT found that vitamin C prevented postangioplasty restenosis [165], and another that vitamin C prevented myocardial injury after elective PCI [166]. An RCT on patients with HF found that a 4-week vitamin C administration increased the distance covered in a 6-min walk test by 49 m (26%) [167].

As to the possible effects of low vitamin C levels on HF, certain cohort studies are interesting. Among males of the EPIC study, a vitamin C plasma level of 23 µmol/L was associated with a 40% higher risk of HF compared with 70 µmol/L [168]. Another study with males found that a vitamin C plasma level of < 14 µmol/L was associated with twice the risk of HF compared with > 40 µmol/L [169]. Two further studies found that low vitamin C intake in patients with HF was associated with higher rates of cardiac events [170, 171].

Two Mendelian randomization studies on vitamin C and cardiac outcomes did not find association [172, 173]. However, the comparisons in these two studies corresponded to vitamin C groups with mean plasma levels of 47.3 and 52.7 µmol/L [174, 175]. Comparison of two groups with such high and closely similar vitamin C levels is uninformative about the effects of low plasma levels for cardiac health; compare with the plasma levels in the above mentioned cohort studies.

Case reports indicate that vitamin C deficiency can lead to cardiomegaly [176, 177], hypertension [178], hypotension [179–183], lower limb pitting edema [184–189], and vasoplegia [190]. Other case reports described exertional dyspnea together with cardiac tamponade [191], lightheadedness [192], or lightheadedness and abnormalities of LV wall motion [193].

In the Sheffield study on experimental vitamin C deficiency, 2 participants had chest pain and ECG changes [149–151]. The 2 participants showed striking improvement within a few days after vitamin C administration. In the Iowa City study on experimental vitamin C deficiency, 2 participants had exertional dyspnea during deprivation [142]. In addition, 4 participants had reduced responsiveness of resistance vessels of the forearm to lower body negative pressure as compared to the state after vitamin C repletion [194]. In these two studies, there were 4 participants with chest pain or dyspnea due to vitamin C deprivation, which is 27% of all vitamin C deficient participants in the two studies (4/15).

Finally, in physiological laboratories vitamin C administration has shown a wide range of effects that are relevant to the cardiac system, such as increasing coronary artery diameter [45–49], coronary flow [195–201], improving effects of inotropic agents [202–206], increasing baroreflex sensitivity [207–210], alleviating postural tachycardia [211], increasing left ventricular diastolic function [212] and preventing nitrate tolerance [213–215]. Most of these physiological studies were short. However, our current analysis indicates that some of the reported mechanisms may have long term relevance.

After we received the reviewer reports, we noted a report on two cases of PH induced by scurvy [216]. The two cases are consistent with the set we analyzed, see a brief summary in our Supplement 1. We did not append them to our analysis.

## Conclusions

We identified 32 case reports that are informative about the effects of vitamin C deficiency on PH. The majority of the included cases seem to fall into the PAH group. Thus, PH should be included in the list of conditions associated with scurvy.

Neuropsychiatric symptoms such as ASD were common in the reported cases and vitamin C intake should be considered in such patients. It would seem appropriate to measure plasma vitamin C levels of patients with all forms of PH and examine the possible effects of vitamin C administration on all PH groups. The potential role of vitamin C for preventing and treating HF should be investigated in populations that have particularly low vitamin C intakes.

### Supplementary Information

**Supplementary Material 1.**

**Supplementary Material 2.**

## Data Availability

All data generated or analysed during this study are included in this published article [and its supplementary information files].

## References

[1] Lind J. A Treatise of the Scurvy in Three Parts. Edinburgh, UK, A. Kincaid and A. Donaldson. 1753. 10.1017/CBO9781107256644. https://archive.org/details/b30507054.

[2] Hemilä H. The symptoms of vitamin C deficiency. 1. Observations and descriptions by Hess, Lind, Trotter and Blane. Zenodo. 2024. 10.5281/zenodo.10685194.

[3] Hess AF (1920). Scurvy: Past and Present.

[4] Erdheim J (1918). The heart in infantile scurvy [in German, translation available]. Wien Klin Wochenschr.

[5] Darling ST (1914). The pathologic affinities of beriberi and scurvy. JAMA.

[6] Hess AF (1915). Infantile scurvy. II. A new aspect of the symptomatology, pathology and diet. JAMA.

[7] Hess AF (1918). Focal degeneration of the lumbar cord in a case of infantile scurvy. J Infect Dis.

[8] Hess AF (1917). Subacute and latent infantile scurvy: the cardiorespiratory syndrome (a new sign). JAMA.

[9] Bierich R (1919). Scurvy [in German, translation available]. Dtsch Arch Klin Med.

[10] Swarbreck A (1932). Avitaminosis. Br Med J.

[11] Follis RH (1942). Sudden death in infants with scurvy. J Pediatr.

[12] Comrie JD (1920). Scurvy in North Russia. Edinb Med J.

[13] Platt R (1936). Scurvy as the result of dietetic treatment. Lancet.

[14] Taylor S (1937). Scurvy and carditis. Lancet.

[15] Evans W (1938). Vitamin C in heart failure. Lancet.

[16] Shaffer CF (1944). The diuretics effect of ascorbic acid: preliminary report on its use in cardiac decompensation. JAMA.

[17] Editorial (1944). Ascorbic acid as a diuretic. Lancet.

[18] Holst A, Frölich T (1907). Experimental studies relating to ‘ship-beri-beri’ and scurvy. J Hyg (Lond).

[19] Schultz MP (1936). Cardiovascular and arthritic lesions in guinea-pigs with chronic scurvy and hemolytic streptococcic infections. Arch Pathol.

[20] Höjer JA (1924). Histo-pathological studies. In: studies in scurvy. Acta Paediatr.

[21] Meyer A (1928). The minute morphology of experimental scurvy in the guinea pig. Stanf Univ Publications Univ Ser Med Sci.

[22] Menten ML, King CG (1935). The influence of vitamin C level upon resistance to diphtheria toxin: II. Production of diffuse hyperplastic arteriosclerosis and degeneration in various organs. J Nutr.

[23] Bessey OA, Menten ML, King CG (1934). Pathologic changes in organs of scorbutic guinea pigs. Proc Soc Exp Biol Med.

[24] Findlay GM (1921). The blood and blood-vessels in guinea pig scurvy. J Pathol Bacteriol.

[25] Findlay GM (1923). The relation of vitamin C to bacterial infection. J Pathol Bacteriol.

[26] Stimson AM, Hedley OF, Rose E (1934). Notes on experimental rheumatic fever. Public Health Rep.

[27] Rinehart JF, Mettier SR (1934). The heart valves and muscle in experimental scurvy with superimposed infection: with notes on the similarity of the lesions to those of rheumatic fever. Am J Pathol.

[28] Taylor S (1937). Scurvy and carditis. Lancet.

[29] McBroom J, Sunderland DA, Mote JR, Jones TD (1937). Effect of acute scurvy on the guinea-pig heart. Arch Path.

[30] Maeda N, Hagihara H, Nakata Y, Hiller S, Wilder J, Reddick R (2000). Aortic wall damage in mice unable to synthesize ascorbic acid. Proc Natl Acad Sci USA.

[31] Xiang RP, Sun WD, Wang JY, Wang XL (2002). Effect of vitamin C on pulmonary hypertension and muscularisation of pulmonary arterioles in broilers. Br Poult Sci.

[32] Zamani Moghaddam AK, Hassanpour H, Mokhtari A (2009). Oral supplementation with vitamin C improves intestinal mucosa morphology in the pulmonary hypertensive broiler chicken. Br Poult Sci.

[33] Humbert M, Kovacs G, Hoeper MM, Badagliacca R, Berger RMF, Brida M, Carlsen J, Coats AJS, Escribano-Subias P, Ferrari P, Ferreira DS, Ghofrani HA, Giannakoulas G, Kiely DG, Mayer E, Meszaros G, Nagavci B, Olsson KM, Pepke-Zaba J, Quint JK, Rådegran G, Simonneau G, Sitbon O, Tonia T, Toshner M, Vachiery JL, Vonk Noordegraaf A, Delcroix M, Rosenkranz S, ESC/ERS Scientific Document Group (2022). 2022 ESC/ERS guidelines for the diagnosis and treatment of pulmonary hypertension. Eur Heart J.

[34] Hassoun PM (2021). Pulmonary arterial hypertension. N Engl J Med.

[35] Newman JH (2020). Pulmonary hypertension by the method of Paul Wood. Chest.

[36] Gomberg-Maitland M, Dufton C, Oudiz RJ, Benza RL (2011). Compelling evidence of long-term outcomes in pulmonary arterial hypertension? A clinical perspective. J Am Coll Cardiol.

[37] Houston BA, Brittain EL, Tedford RJ (2023). Right ventricular failure. N Engl J Med.

[38] Kolte D, Lakshmanan S, Jankowich MD, Brittain EL, Maron BA, Choudhary G (2018). Mild pulmonary hypertension is associated with increased mortality: a systematic review and meta-analysis. J Am Heart Assoc.

[39] Gao Y, Chen T, Raj JU (2016). Endothelial and smooth muscle cell interactions in the pathobiology of pulmonary hypertension. Am J Respir Cell Mol Biol.

[40] Voelkel NF, Gomez-Arroyo J, Abbate A, Bogaard HJ, Nicolls MR (2012). Pathobiology of pulmonary arterial hypertension and right ventricular failure. Eur Respir J.

[41] Pullamsetti SS, Mamazhakypov A, Weissmann N, Seeger W, Savai R (2020). Hypoxia-inducible factor signaling in pulmonary hypertension. J Clin Invest.

[42] Huston JH, Ryan JJ (2016). The emerging role of epigenetics in pulmonary arterial hypertension: an important avenue for clinical trials. Pulm Circ.

[43] Agrawal V, Hemnes AR, Shelburne NJ, Fortune N, Fuentes JL, Colvin D, et al. L-Carnitine therapy improves right heart dysfunction through Cpt1-dependent fatty acid oxidation. Pulm Circ. 2022;12(3):e12107. 10.1002/pul2.12107. 10.1002/pul2.12107 PMC9326551 35911183

[44] Breda AP, Pereira de Albuquerque AL, Jardim C, Morinaga LK, Suesada MM, Fernandes CJ, Dias B, Lourenço RB, Salge JM, Souza R (2014). Skeletal muscle abnormalities in pulmonary arterial hypertension. PLoS ONE.

[45] Solzbach U, Hornig B, Jeserich M, Just H (1997). Vitamin C improves endothelial dysfunction of epicardial coronary arteries in hypertensive patients. Circulation.

[46] Kugiyama K, Motoyama T, Hirashima O, Ohgushi M, Soejima H, Misumi K, Kawano H, Miyao Y, Yoshimura M, Ogawa H, Matsumura T, Sugiyama S, Yasue H (1998). Vitamin C attenuates abnormal vasomotor reactivity in spasm coronary arteries in patients with coronary spastic angina. J Am Coll Cardiol.

[47] Jeserich M, Schindler T, Olschewski M, Unmüssig M, Just H, Solzbach U (1999). Vitamin C improves endothelial function of epicardial coronary arteries in patients with hypercholesterolaemia or essential hypertension-assessed by cold pressor testing. Eur Heart J.

[48] Schindler TH, Magosaki N, Jeserich M, Olschewski M, Nitzsche E, Holubarsch C, Solzbach U, Just H (2001). Effect of ascorbic acid on endothelial dysfunction of epicardial coronary arteries in chronic smokers assessed by cold pressor testing. Cardiology.

[49] Tousoulis D, Xenakis C, Tentolouris C, Davies G, Antoniades C, Crake T, Stefanadis C (2005). Effects of vitamin C on intracoronary L-arginine dependent coronary vasodilatation in patients with stable angina. Heart.

[50] May JM, Harrison FE (2013). Role of vitamin C in the function of the vascular endothelium. Antioxid Redox Signal.

[51] May JM (2000). How does ascorbic acid prevent endothelial dysfunction?. Free Radic Biol Med.

[52] Holowatz LA (2011). Ascorbic acid: what do we really NO?. J Appl Physiol.

[53] Beetens JR, Herman AG (1983). Vitamin C increases the formation of prostacyclin by aortic rings from various species and neutralizes the inhibitory effect of 15-hydroperoxy-arachidonic acid. Br J Pharmacol.

[54] Srivastava KC (1985). Ascorbic acid enhances the formation of prostaglandin E1 in washed human platelets and prostacyclin in rat aortic rings. Prostaglandins Leukot Med.

[55] Beetens JR, Coene MC, Veheyen A, Zonnekeyn L, Herman AG (1986). Vitamin C increases the prostacyclin production and decreases the vascular lesions in experimental atherosclerosis in rabbits. Prostaglandins.

[56] Eldor A, Vlodavsky I, Riklis E, Fuks Z (1987). Recovery of prostacyclin capacity of irradiated endothelial cells and the protective effect of vitamin C. Prostaglandins.

[57] Mahfouz MM, Kummerow FA (2004). Vitamin C or vitamin B6 supplementation prevent the oxidative stress and decrease of prostacyclin generation in homocysteinemic rats. Int J Biochem Cell Biol.

[58] Basili S, Pignatelli P, Tanzilli G, Mangieri E, Carnevale R, Nocella C, Di Santo S, Pastori D, Ferroni P, Violi F (2011). Anoxia-reoxygenation enhances platelet thromboxane A2 production via reactive oxygen species-generated NOX2: effect in patients undergoing elective percutaneous coronary intervention. Arterioscler Thromb Vasc Biol.

[59] Böhm F, Settergren M, Pernow J (2007). Vitamin C blocks vascular dysfunction and release of interleukin-6 induced by endothelin-1 in humans in vivo. Atherosclerosis.

[60] Mandl J, Szarka A, Bánhegyi G (2009). Vitamin C: update on physiology and pharmacology. Br J Pharmacol.

[61] Padayatty SJ, Levine M, Vitamin C (2016). The known and the unknown and Goldilocks. Oral Dis.

[62] Young JI, Züchner S, Wang G (2015). Regulation of the epigenome by vitamin C. Annu Rev Nutr.

[63] Brabson JP, Leesang T, Mohammad S, Cimmino L (2021). Epigenetic regulation of genomic stability by vitamin C. Front Genet.

[64] Hughes RE, Hurley RJ, Jones E (1980). Dietary ascorbic acid and muscle carnitine in guinea-pigs. Br J Nutr.

[65] Rebouche CJ. Ascorbic acid and carnitine biosynthesis. Am J Clin Nutr. 1991;54(6 Suppl):1147S–52S. 10.1093/ajcn/54.6.1147s. 10.1093/ajcn/54.6.1147s 1962562

[66] Jones E, Hughes RE (1982). Influence of oral carnitine on the body weight and survival time of avitaminotic-C guinea pigs. Nutr Rep Int.

[67] Dalldorf G (1929). The lesions in the skeletal muscles in experimental scorbutus. J Exp Med.

[68] Rossman MJ, Garten RS, Groot HJ, Reese V, Zhao J, Amann M, Richardson RS (2013). Ascorbate infusion increases skeletal muscle fatigue resistance in patients with chronic obstructive pulmonary disease. Am J Physiol Regul Integr Comp Physiol.

[69] Greiner S, Jud A, Aurich M, Hess A, Hilbel T, Hardt S, Katus HA, Mereles D (2014). Reliability of noninvasive assessment of systolic pulmonary artery pressure by Doppler echocardiography compared to right heart catheterization: analysis in a large patient population. J Am Heart Assoc.

[70] Lafitte S, Pillois X, Reant P, Picard F, Arsac F, Dijos M, Coste P, Dos Santos P, Roudaut R (2013). Estimation of pulmonary pressures and diagnosis of pulmonary hypertension by Doppler echocardiography: a retrospective comparison of routine echocardiography and invasive hemodynamics. J Am Soc Echocardiogr.

[71] Amsallem M, Sternbach JM, Adigopula S, Kobayashi Y, Vu TA, Zamanian R, Liang D, Dhillon G, Schnittger I, McConnell MV, Haddad F (2016). Addressing the controversy of estimating pulmonary arterial pressure by echocardiography. J Am Soc Echocardiogr.

[72] Chemla D, Humbert M, Sitbon O, Montani D, Hervé P (2015). Systolic and mean pulmonary artery pressures: are they interchangeable in patients with pulmonary hypertension?. Chest.

[73] Kind T, Faes TJ, Vonk-Noordegraaf A, Westerhof N (2011). Proportional relations between systolic, diastolic and mean pulmonary artery pressure are explained by vascular properties. Cardiovasc Eng Technol.

[74] McQuillan BM, Picard MH, Leavitt M, Weyman AE (2001). Clinical correlates and reference intervals for pulmonary artery systolic pressure among echocardiographically normal subjects. Circulation.

[75] Hatle L, Brubakk A, Tromsdal A, Angelsen B (1978). Noninvasive assessment of pressure drop in mitral stenosis by Doppler ultrasound. Br Heart J.

[76] Abbas F, Ha LD, Sterns R, von Doenhoff L (2016). Reversible right heart failure in scurvy: rediscovery of an old observation. Circ Heart Fail.

[77] Abe K, Kibe R, David K, Reddy V, Allard B, Fakaosita M. Reversible right-sided heart failure and pulmonary hypertension caused by scurvy in a 7-year-old boy with autism spectrum disorder and a review of the literature. Paediatr Int Child Health. 2023;43(4):95–9. 10.1080/20469047.2021.1901406. 10.1080/20469047.2021.1901406 34033530

[78] Azar J, Varasteh A, Iltchev D, Soliman M, Baez V, Altaqi B (2019). A very uncommon case of pulmonary arterial hypertension. Am J Med Case Rep.

[79] Azar J, Ayyad M, Jaber Y, Ayasa LA (2023). Scurvy-induced pulmonary arterial hypertension. BMJ Case Rep.

[80] Benhamed A, Bernard M, Tazarourte K (2019). Scurvy case revealed by dyspnea [in French]. Ann Françaises De Médecine d’Urgence.

[81] Conte L, Louden J, Weber LA (2021). Multivalve dysfunction and cardiogenic shock linked to scurvy: a case report. Anatol J Cardiol.

[82] Dean T, Kaushik N, Williams S, Zinter M, Kim P (2019). Cardiac arrest and pulmonary hypertension in scurvy: a case report. Pulm Circ.

[83] Duvall MG, Pikman Y, Kantor DB, Ariagno K, Summers L, Sectish TC, Mullen MP (2013). Pulmonary hypertension associated with scurvy and vitamin deficiencies in an autistic child. Pediatrics.

[84] Ferreira CCG, de Sá Pereira Belfort D, Neto PMC, Gouveia PADC (2020). Reversible pulmonary hypertension secondary to scurvy in a patient with a psychiatric disorder: a case report and literature review. Eur J Case Rep Intern Med.

[85] Frank BS, Runciman M, Manning WA, Ivy DD, Abman SH, Howley L. Pulmonary hypertension secondary to scurvy in a developmentally typical child. J Pediatr. 2019;208:291.e2. 10.1016/j.jpeds.2018.12.068. 10.1016/j.jpeds.2018.12.068 30738657

[86] Gayen SK, Abdelrahman AA, Preston IR, Petit RD, Hill NS (2020). Vitamin C deficiency-induced pulmonary arterial hypertension. Chest.

[87] Ghulam Ali S, Pepi M (2018). A very uncommon case of pulmonary hypertension. CASE (Phila).

[88] Gilmore AC, Van Dongen M, Despotes KA, Al-Qadi MO (2021). Reversible pulmonary hypertension due to severe vitamin C deficiency [Abstract]. Am J Respir Crit Care Med.

[89] Ichiyanagi S, Takeshita I, Kandil AI, Miyazu M, Kojima T (2019). Pulmonary hypertensive crisis during general anesthesia in a 3-year-old autistic boy with undiagnosed scurvy, undergoing cardiac catheterization: a case report. Pract.

[90] Kupari M, Rapola J (2012). Reversible pulmonary hypertension associated with vitamin C deficiency. Chest.

[91] Kurnick A, Zaveri S, Tadayoni A, Chandrakumar HP, John S (2023). Reversible severe pulmonary hypertension and right heart failure with cardiogenic shock due to scurvy: a case report. Eur Heart J Case Rep.

[92] Mehta CL, Cripps D, Bridges AJ (1996). Systemic pseudovasculitis from scurvy in anorexia nervosa. Arthritis Rheum.

[93] Mertens MT, Gertner E (2011). Rheumatic manifestations of scurvy: a report of three recent cases in a major urban center and a review. Semin Arthritis Rheum.

[94] Nagamatsu S, Nahum Avi, McEvoy CE. Pulmonary hypertension secondary to vitamin C deficiency [Abstract]. Chest 2009;136(4 Supplement):4S. 10.1378/chest.136.4_MeetingAbstracts.4S-e.

[95] Nariai R, Mafune R, Ide K, Nishimura N, Ono H (2022). Pulmonary hypertension caused by vitamin C deficiency in a 2-year-old girl. Pediatr Int.

[96] Penn EH, Olenchock BA, Marston NA (2019). A shocking deficiency. Circulation.

[97] Petersen E. When life doesn’t give you lemons: a rare case of acute heart failure [Abstract]. Published at Hospital Medicine 2019, March 24–27, National Harbor, Md. Abstract 467. https://shmabstracts.org/abstract/when-life-doesnt-give-you-lemons-a-rare-case-of-acute-heart-failure.

[98] Quinn LA, Gilley SP, Ta AD, Frank BS, Foley CB, Moore JM (2022). Case report: pulmonary hypertensive crisis leading to cardiac arrest during endoscopic evaluation in a 6-year-old boy with autism, severe malnutrition, and undiagnosed scurvy. Front Pediatr.

[99] Ratanachu-Ek S, Sukswai P, Jeerathanyasakun Y, Wongtapradit L (2003). Scurvy in pediatric patients: a review of 28 cases. J Med Assoc Thai.

[100] Sakamornchai W, Dumrongwongsiri O, Siwarom S (2022). Case report: vitamin C combined with multiple micronutrient deficiencies is associated with pulmonary arterial hypertension in children with autistic spectrum disorder. Front Nutr.

[101] Shah V, Shah RN, Greene L, DiMarino LM (2021). Severe pulmonary hypertension in a patient with scurvy: can a vitamin reverse it?. Case Rep Med.

[102] Singh L, Gay E, Waxman A. A sweet remedy for severe pulmonary arterial hypertension [Abstract]. American Thoracic Society 2017 Conference. Abstract A6187. https://www.atsjournals.org/doi/abs/10.1164/ajrccm-conference.2017.195.1_MeetingAbstracts.A6187.

[103] Tan JWY, Lee OPE, Leong MC (2021). Vitamin C deficiency as an unusual cause of pulmonary hypertension and refusal to walk. Cardiol Young.

[104] Ueki M, Sakamoto K, Nishioka N, Ohata H, Nobuta T, Takezaki S, Manabe A, Yamada M (2023). Rheumatologic manifestations with elevated levels of IL-6, IL-17A, and IL-23 in a patient with scurvy. Mod Rheumatol Case Rep.

[105] Valencia E, Vakili K, Thiagarajan RR, Mullen MP, Fynn-Thompson F, Weldon CB, Duvall MG (2022). Case 2-2022: an adolescent male in cardiac arrest 3 days after liver transplantation for end-stage liver disease. Pediatr Crit Care Med.

[106] Fleming S, Thompson M, Stevens R, Heneghan C, Plüddemann A, Maconochie I, Tarassenko L, Mant D (2011). Normal ranges of heart rate and respiratory rate in children from birth to 18 years of age: a systematic review of observational studies. Lancet.

[107] Colman R, Whittingham H, Tomlinson G, Granton J (2014). Utility of the physical examination in detecting pulmonary hypertension. A mixed methods study. PLoS ONE.

[108] Kaul S, Tei C, Hopkins JM, Shah PM (1984). Assessment of right ventricular function using two-dimensional echocardiography. Am Heart J.

[109] Ryan T, Petrovic O, Dillon JC, Feigenbaum H, Conley MJ, Armstrong WF (1985). An echocardiographic index for separation of right ventricular volume and pressure overload. J Am Coll Cardiol.

[110] Shibayama J, Tada H, Morita M, Yoshida S, Sakata K, Usui S, Kawashiri MA, Takamura M (2022). A case of pulmonary hypertension in a 67-year-old woman with thiamine deficiency following partial gastrectomy and exacerbated by diuretics. Am J Case Rep.

[111] Sakurai K, Fujiwara N, Takahashi K, Nakayashiro M (2019). Excessive soft drink may induce pulmonary hypertension via thiamine deficiency. Pediatr Int.

[112] Asakura T, Kodera S, Kanda J, Ikeda M (2013). Thiamine-responsive pulmonary hypertension. BMJ Case Rep.

[113] Ghio S, Klersy C, Magrini G, D’Armini AM, Scelsi L, Raineri C, Pasotti M, Serio A, Campana C, Viganò M (2010). Prognostic relevance of the echocardiographic assessment of right ventricular function in patients with idiopathic pulmonary arterial hypertension. Int J Cardiol.

[114] Baggen VJM, Driessen MMP, Post MC, van Dijk AP, Roos-Hesselink JW, van den Bosch AE, Takkenberg JJM, Sieswerda GT (2016). Echocardiographic findings associated with mortality or transplant in patients with pulmonary arterial hypertension: a systematic review and meta-analysis. Neth Heart J.

[115] Gorter TM, Hoendermis ES, van Veldhuisen DJ, Voors AA, Lam CS, Geelhoed B, Willems TP, van Melle JP (2016). Right ventricular dysfunction in heart failure with preserved ejection fraction: a systematic review and meta-analysis. Eur J Heart Fail.

[116] Martha JW, Pranata R, Wibowo A, Lim MA (2021). Tricuspid annular plane systolic excursion (TAPSE) measured by echocardiography and mortality in COVID-19: a systematic review and meta-analysis. Int J Infect Dis.

[117] Karatasakis GT, Karagounis LA, Kalyvas PA, Manginas A, Athanassopoulos GD, Aggelakas SA, Cokkinos DV (1998). Prognostic significance of echocardiographically estimated right ventricular shortening in advanced heart failure. Am J Cardiol.

[118] McCabe C, Vranesic II, Verdes MC, Kempny A, Khan U, Price L, Gatzoulis MA, Dimopoulos K, Wort SJ, Li W (2019). Right ventricular systolic to diastolic duration ratio: a novel predictor of outcome in adult idiopathic pulmonary arterial hypertension. Int J Cardiol.

[119] Brierre G, Blot-Souletie N, Degano B, Têtu L, Bongard V, Carrié D (2010). New echocardiographic prognostic factors for mortality in pulmonary arterial hypertension. Eur J Echocardiogr.

[120] Richter MJ, Fortuni F, Alenezi F, D’Alto M, Badagliacca R, Brunner NW, van Dijk AP, Douschan P, Gall H, Ghio S, Giudice FL, Grünig E, Haddad F, Howard L, Rajagopal S, Stens N, Stolfo D, Thijssen DHJ, Vizza CD, Zamanian RT, Zhong L, Seeger W, Ghofrani HA, Tello K (2023). Imaging the right atrium in pulmonary hypertension: a systematic review and meta-analysis. J Heart Lung Transpl.

[121] Hendriks PM, van de Groep LD, Veen KM, van Thor MCJ, Meertens S, Boersma E, Boomars KA, Post MC, van den Bosch AE (2022). Prognostic value of brain natriuretic peptides in patients with pulmonary arterial hypertension: a systematic review and meta-analysis. Am Heart J.

[122] Hróbjartsson A, Gøtzsche PC (2010). Placebo interventions for all clinical conditions. Cochrane Database Syst Rev.

[123] Wood L, Egger M, Gluud LL, Schulz KF, Jüni P, Altman DG, Gluud C, Martin RM, Wood AJ, Sterne JA (2008). Empirical evidence of bias in treatment effect estimates in controlled trials with different interventions and outcomes: meta-epidemiological study. BMJ.

[124] Simonneau G, Barst RJ, Galie N, Naeije R, Rich S, Bourge RC, Keogh A, Oudiz R, Frost A, Blackburn SD, Crow JW, Rubin LJ, Treprostinil Study Group (2002). Continuous subcutaneous infusion of treprostinil, a prostacyclin analogue, in patients with pulmonary arterial hypertension: a double-blind, randomized, placebo-controlled trial. Am J Respir Crit Care Med.

[125] Galiè N, Rubin Lj, Hoeper M, Jansa P, Al-Hiti H, Meyer G, Chiossi E, Kusic-Pajic A, Simonneau G (2008). Treatment of patients with mildly symptomatic pulmonary arterial hypertension with bosentan (EARLY study): a double-blind, randomised controlled trial. Lancet.

[126] Jansa P, Pulido T (2018). Macitentan in pulmonary arterial hypertension: a focus on combination therapy in the SERAPHIN trial. Am J Cardiovasc Drugs.

[127] Padayatty SJ, Sun H, Wang Y, Riordan HD, Hewitt SM, Katz A, Wesley RA, Levine M (2004). Vitamin C pharmacokinetics: implications for oral and intravenous use. Ann Intern Med.

[128] Hemilä H, Chalker E (2019). Vitamin C can shorten the length of stay in the ICU: a meta-analysis. Nutrients.

[129] Hemilä H, Suonsyrjä T (2017). Vitamin C for preventing atrial fibrillation in high risk patients: a systematic review and meta-analysis. BMC Cardiovasc Disord.

[130] Hemilä H, Chalker E, de Man AME (2022). Vitamin C may improve left ventricular ejection fraction: a meta-analysis. Front Cardiovasc Med.

[131] Hemilä H (2013). Vitamin C may alleviate exercise-induced bronchoconstriction: a meta-analysis. BMJ Open.

[132] Hemilä H (2013). Vitamin C and common cold-induced asthma: a systematic review and statistical analysis. Allergy Asthma Clin Immunol.

[133] Benza RL, Miller DP, Barst RJ, Badesch DB, Frost AE, McGoon MD (2012). An evaluation of long-term survival from time of diagnosis in pulmonary arterial hypertension from the REVEAL Registry. Chest.

[134] Moreira EM, Gall H, Leening MJ, Lahousse L, Loth DW, Krijthe BP, Kiefte-de Jong JC, Brusselle GG, Hofman A, Stricker BH, Ghofrani HA, Franco OH, Felix JF (2015). Prevalence of pulmonary hypertension in the general population: the Rotterdam Study. PLoS ONE.

[135] Schleicher RL, Carroll MD, Ford ES, Lacher DA (2009). Serum vitamin C and the prevalence of vitamin C deficiency in the United States: 2003–2004 National Health and Nutrition Examination Survey (NHANES). Am J Clin Nutr.

[136] Mosdøl A, Erens B, Brunner EJ (2008). Estimated prevalence and predictors of vitamin C deficiency within UK’s low-income population. J Public Health (Oxf).

[137] Villalpando S, Montalvo-Velarde I, Zambrano N, García-Guerra A, Ramírez-Silva CI, Shamah-Levy T, et al. Vitamins A, and C and folate status in Mexican children under 12 years and women 12-49 years: a probabilistic national survey. Salud Publica Mex. 2003;45(Suppl 4):S508–19. 10.1590/s0036-36342003001000007. 10.1590/s0036-36342003001000007 14746045

[138] Ravindran RD, Vashist P, Gupta SK, Young IS, Maraini G, Camparini M, Jayanthi R, John N, Fitzpatrick KE, Chakravarthy U, Ravilla TD, Fletcher AE (2011). Prevalence and risk factors for vitamin C deficiency in north and south India: a two centre population based study in people aged 60 years and over. PLoS ONE.

[139] Paalanen L, Prättälä R, Alfthan G, Salminen I, Laatikainen T (2014). Vegetable and fruit consumption, education and plasma vitamin C concentration in Russian and Finnish Karelia, 1992–2002. Public Health Nutr.

[140] Raynaud-Simon A, Cohen-Bittan J, Gouronnec A, Pautas E, Senet P, Verny M, Boddaert J (2010). Scurvy in hospitalized elderly patients. J Nutr Health Aging.

[141] Hodges RE (1971). What’s new about scurvy?. Am J Clin Nutr.

[142] Hodges RE, Hood J, Canham JE, Sauberlich HE, Baker EM (1971). Clinical manifestations of ascorbic acid deficiency in man. Am J Clin Nutr.

[143] Drummond JC, Wilbraham A. William Stark, M.D.: an eighteenth century experiment in nutrition. Lancet. 1935;226:459–63. 10.1016/S0140-6736(00)94709-3.

[144] Carpenter KJ. The History of Scurvy and Vitamin C. Cambridge University Press, 1988. https://www.cambridge.org/fi/academic/subjects/history/history-medicine/history-scurvy-and-vitamin-c.

[145] Hashizume H, Ishikawa Y, Ajima S (2023). Modern scurvy revisited: Japanese cases of a forgotten disease. J Dermatol.

[146] Doll S, Ricou B (2013). Severe vitamin C deficiency in a critically ill adult: a case report. Eur J Clin Nutr.

[147] Maltos AL, Portari GV, Saldanha JC, Bernardes Júnior AG, Pardi GR, da Cunha DF (2012). Scurvy in an alcoholic malnourished cirrhotic man with spontaneous bacterial peritonitis. Clin (Sao Paulo).

[148] Allender WJ (1982). Post mortem tissue levels of ascorbic acid in a scurvy case. J Anal Toxicol.

[149] Medical Research Council (1948). Vitamin-C requirement of human adults; experimental study of vitamin-C deprivation in man. Lancet.

[150] Krebs HA (1953). The Sheffield experiment on the vitamin C requirement of human adults. Proc Nutr Soc.

[151] Bartley W, Krebs HA, O’Brien JRP (1953). Vitamin C requirement of human adults: case histories. Spec Rep Ser Med Res Counc (GB).

[152] Shafar J (1967). Rapid reversion of electrocardiographic abnormalities after treatment in two cases of scurvy. Lancet.

[153] Sament S (1970). Cardiac disorders in scurvy. N Engl J Med.

[154] World Medical Association. WMA Declaration of Helsinki—Ethical Principles for Medical Research Involving Human Subjects. 2022. https://www.wma.net/policies-post/wma-declaration-of-helsinki-ethical-principles-for-medical-research-involving-human-subjects.

[155] Lamontagne F, Masse MH, Menard J, Sprague S, Pinto R, Heyland DK, Cook DJ, Battista MC, Day AG, Guyatt GH, Kanji S, Parke R, McGuinness SP, Tirupakuzhi Vijayaraghavan BK, Annane D, Cohen D, Arabi YM, Bolduc B, Marinoff N, Rochwerg B, Millen T, Meade MO, Hand L, Watpool I, Porteous R, Young PJ, D’Aragon F, Belley-Cote EP, Carbonneau E, Clarke F, Maslove DM, Hunt M, Chassé M, Lebrasseur M, Lauzier F, Mehta S, Quiroz-Martinez H, Rewa OG, Charbonney E, Seely AJE, Kutsogiannis DJ, LeBlanc R, Mekontso-Dessap A, Mele TS, Turgeon AF, Wood G, Kohli SS, Shahin J, Twardowski P, Adhikari NKJ (2022). LOVIT investigators and the Canadian Critical Care Trials Group. Intravenous vitamin C in adults with sepsis in the intensive care unit. N Engl J Med.

[156] Hemilä H, Chalker E (2023). Abrupt termination of vitamin C from ICU patients may increase mortality: secondary analysis of the LOVIT trial. Eur J Clin Nutr.

[157] LOVIT-COVID Investigators, on behalf of the Canadian Critical Care Trials Group, and the, REMAP-CAP Investigators; Adhikari NKJ, Hashmi M, Tirupakuzhi Vijayaraghavan BK, Haniffa R, Beane A et al. Intravenous vitamin C for patients hospitalized with COVID-19: two harmonized randomized clinical trials. JAMA. 2023;330(18):1745–1759. 10.1001/jama.2023.21407. 10.1001/jama.2023.21407 PMC10600726 37877585

[158] Hemilä H. Divergence in mortality at 5 days is overlooked by Adhikari. (2023) in a trial on vitamin C for COVID-19 patients. Pubpeer. 2024. https://pubpeer.com/publications/38194670B815AEE6450D922AA405E2#2.

[159] Vallabhajosyula S, Kumar M, Pandompatam G, Sakhuja A, Kashyap R, Kashani K, Gajic O, Geske JB, Jentzer JC (2017). Prognostic impact of isolated right ventricular dysfunction in sepsis and septic shock: an 8-year historical cohort study. Ann Intensive Care.

[160] Winkelhorst JC, Bootsma IT, Koetsier PM, de Lange F, Boerma EC (2020). Right ventricular function and long-term outcome in sepsis: a retrospective cohort study. Shock.

[161] Glasziou P, Chalmers I, Rawlins M, McCulloch P (2007). When are randomised trials unnecessary? Picking signal from noise. BMJ.

[162] Juraschek SP, Guallar E, Appel LJ, Miller ER (2012). Effects of vitamin C supplementation on blood pressure: a meta-analysis of randomized controlled trials. Am J Clin Nutr.

[163] Rozemeijer S, Hemilä H, van Baaren M, de Man AME (2023). Vitamin C may reduce troponin and CKMB levels after PCI and CABG: a meta-analysis. BMC Cardiovasc Disord.

[164] Ashor AW, Lara J, Mathers JC, Siervo M (2012). Effect of vitamin C on endothelial function in health and disease: a systematic review and meta-analysis of randomised controlled trials. Atherosclerosis.

[165] Tomoda H, Yoshitake M, Morimoto K, Aoki N (1996). Possible prevention of postangioplasty restenosis by ascorbic acid. Am J Cardiol.

[166] Wang ZJ, Hu WK, Liu YY, Shi DM, Cheng WJ, Guo YH, Yang Q, Zhao YX, Zhou YJ (2014). The effect of intravenous vitamin C infusion on periprocedural myocardial injury for patients undergoing elective percutaneous coronary intervention. Can J Cardiol.

[167] Ho CC. Effects of antioxidant on cardiovascular performance, exercise capacity, and functional status in patients with chronic heart failure [PhD thesis]. Cleveland, Ohio: Case Western Reserve University; 2007. Available: https://rave.ohiolink.edu/etdc/view?acc_num=case1157980994.

[168] Pfister R, Sharp SJ, Luben R, Wareham NJ, Khaw KT (2011). Plasma vitamin C predicts incident heart failure in men and women in European prospective investigation into Cancer and Nutrition-Norfolk prospective study. Am Heart J.

[169] Wannamethee SG, Bruckdorfer KR, Shaper AG, Papacosta O, Lennon L, Whincup PH (2013). Plasma vitamin C, but not vitamin E, is associated with reduced risk of heart failure in older men. Circ Heart Fail.

[170] Song EK, Kang SM, Vitamin C (2018). Deficiency, high-sensitivity C-reactive protein, and cardiac event-free survival in patients with heart failure. J Cardiovasc Nurs.

[171] Wu JR, Song EK, Moser DK, Lennie TA (2019). Dietary vitamin C deficiency is associated with health-related quality of life and cardiac event-free survival in adults with heart failure. J Cardiovasc Nurs.

[172] Chen L, Sun X, Wang Z, Lu Y, Chen M, He Y, Xu H, Zheng L (2021). The impact of plasma vitamin C levels on the risk of cardiovascular diseases and Alzheimer’s disease: a mendelian randomization study. Clin Nutr.

[173] Yuan S, Zheng JS, Mason AM, Burgess S, Larsson SC (2022). Genetically predicted circulating vitamin C in relation to cardiovascular disease. Eur J Prev Cardiol.

[174] Hemilä H, Chalker E (2022). Vitamin C and the risk of atrial fibrillation: mendelian randomization study may be misleading. Clin Nutr.

[175] Hemilä H, Chalker E (2022). Vitamin C and the risk of cardiovascular diseases: mendelian randomization study may be misleading. Eur J Prev Cardiol.

[176] Singh D, Chan W (1974). Cardiomegaly and generalized oedema due to vitamin C deficiency. Singapore Med J.

[177] Kieffer P, Thannberger P, Wilhelm JM, Kieffer C, Schneider F (2001). Multiple organ dysfunction dramatically improving with the infusion of vitamin C: more support for the persistence of scurvy in our welfare society. Intensive Care Med.

[178] Weinstein M, Babyn P, Zlotkin S (2001). An orange a day keeps the doctor away: scurvy in the year 2000. Pediatrics.

[179] Crandon JH, Lund CC, Dill DB (1940). Experimental human scurvy. N Engl J Med.

[180] Zipursky JS, Alhashemi A, Juurlink D (2014). A rare presentation of an ancient disease: scurvy presenting as orthostatic hypotension. BMJ Case Rep.

[181] Bennett SE, Schmitt WP, Stanford FC, Baron JM (2018). Case 22-2018: a 64-year-old man with progressive leg weakness, recurrent falls, and anemia. N Engl J Med.

[182] Zavaleta JR, Burt N (2020). Eighteenth century complications with 21st century general anesthesia: a case report of scurvy. Pract.

[183] Roy-Lavallee J, Bahrani B, Weinstein M, Katzman DK (2020). Scurvy: an unexpected nutritional complication in an adolescent female with anorexia nervosa. J Adolesc Health.

[184] Reed RM (2010). Captain Ignose to the rescue. Am J Med.

[185] Smith A, Di Primio G, Humphrey-Murto S (2011). Scurvy in the developed world. CMAJ.

[186] Fleming JD, Martin B, Card DJ, Mellerio JE (2013). Pain, purpura and curly hairs. Clin Exp Dermatol.

[187] Zammit P (2013). Vitamin C deficiency in an elderly adult. J Am Geriatr Soc.

[188] Dufrost V, Risse J, Malgras A, Barraud H, Jaussaud R, Zuily S, Wahl D (2017). Unexpected cause of bleeding. Am J Med.

[189] Szterenlicht YM, Jarjoui A, Kurd R, Levy L, Munter G (2019). A peculiar case of purpura. Am J Med.

[190] Wieruszewski PM, Nei SD, Maltais S, Schaff HV, Wittwer ED (2018). Vitamin C for vasoplegia after cardiopulmonary bypass: a case series. Pract.

[191] Alnaimat S, Oseni A, Yang Y, Melvani V, Aronson A, Harris K, Panaich S (2019). Missing vitamin C: a case of scorbutic cardiac tamponade. JACC Case Rep.

[192] Pichan C, Dhaliwal G, Cusick A, Saint S, Houchens N (2021). Inadequate support. N Engl J Med.

[193] Meisel JL, McDowell RK (1995). Case 39-1995: a 72-year-old man with exertional dyspnea, fatigue, and extensive ecchymoses and purpuric lesions. N Engl J Med.

[194] Abboud FM, Hood J, Hodges RE, Mayer HE (1970). Autonomic reflexes and vascular reactivity in experimental scurvy in man. J Clin Invest.

[195] Kaufmann PA, Gnecchi-Ruscone T, di Terlizzi M, Schäfers KP, Lüscher TF, Camici PG (2000). Coronary heart disease in smokers: vitamin C restores coronary microcirculatory function. Circulation.

[196] Richartz BM, Werner GS, Ferrari M, Figulla HR (2001). Reversibility of coronary endothelial vasomotor dysfunction in idiopathic dilated cardiomyopathy: acute effects of vitamin C. Am J Cardiol.

[197] Schindler TH, Nitzsche EU, Munzel T, Olschewski M, Brink I, Jeserich M, Mix M, Buser PT, Pfisterer M, Solzbach U, Just H (2003). Coronary vasoregulation in patients with various risk factors in response to cold pressor testing: contrasting myocardial blood flow responses to short- and long-term vitamin C administration. J Am Coll Cardiol.

[198] Teramoto K, Daimon M, Hasegawa R, Toyoda T, Sekine T, Kawata T, Yoshida K, Komuro I (2004). Acute effect of oral vitamin C on coronary circulation in young healthy smokers. Am Heart J.

[199] McNulty PH, Robertson BJ, Tulli MA, Hess J, Harach LA, Scott S, Sinoway LI (2007). Effect of hyperoxia and vitamin C on coronary blood flow in patients with ischemic heart disease. J Appl Physiol.

[200] Basili S, Tanzilli G, Mangieri E, Raparelli V, Di Santo S, Pignatelli P, Violi F (2010). Intravenous ascorbic acid infusion improves myocardial perfusion grade during elective percutaneous coronary intervention: relationship with oxidative stress markers. JACC Cardiovasc Interv.

[201] Gao Z, Spilk S, Momen A, Muller MD, Leuenberger UA, Sinoway LI (2012). Vitamin C prevents hyperoxia-mediated coronary vasoconstriction and impairment of myocardial function in healthy subjects. Eur J Appl Physiol.

[202] Mak S, Newton GE (2001). Vitamin C augments the inotropic response to dobutamine in humans with normal left ventricular function. Circulation.

[203] Mak S, Egri Z, Tanna G, Colman R, Newton GE (2002). Vitamin C prevents hyperoxia-mediated vasoconstriction and impairment of endothelium-dependent vasodilation. Am J Physiol Heart Circ Physiol.

[204] Mak S, Overgaard CB, Newton GE (2005). Effect of vitamin C and L-NMMA on the inotropic response to dobutamine in patients with heart failure. Am J Physiol Heart Circ Physiol.

[205] Kato K, Fukuma N, Kimura-Kato Y, Aisu N, Tuchida T, Mabuchi K, Takano T (2006). Improvement of sympathetic response to exercise by oral administration of ascorbic acid in patients after myocardial infarction. Int J Cardiol.

[206] Shinke T, Shite J, Takaoka H, Hata K, Inoue N, Yoshikawa R, et al. Vitamin C restores the contractile response to dobutamine and improves myocardial efficiency in patients with heart failure after anterior myocardial infarction. Am Heart J. 2007;154(4):645.e1–8. 10.1016/j.ahj.2007.07.005. 10.1016/j.ahj.2007.07.005 17892985

[207] Nightingale AK, Blackman DJ, Field R, Glover NJ, Pegge N, Mumford C, Schmitt M, Ellis GR, Morris-Thurgood JA, Frenneaux MP (2003). Role of nitric oxide and oxidative stress in baroreceptor dysfunction in patients with chronic heart failure. Clin Sci (Lond).

[208] Piccirillo G, Nocco M, Moisè A, Lionetti M, Naso C, di Carlo S, Marigliano V (2003). Influence of vitamin C on baroreflex sensitivity in chronic heart failure [dose correction to grams in 2003;42:e5]. Hypertension.

[209] Monahan KD, Eskurza I, Seals DR (2004). Ascorbic acid increases cardiovagal baroreflex sensitivity in healthy older men. Am J Physiol Heart Circ Physiol.

[210] Bruno RM, Daghini E, Ghiadoni L, Sudano I, Rugani I, Varanini M, Passino C, Emdin M, Taddei S (2012). Effect of acute administration of vitamin C on muscle sympathetic activity, cardiac sympathovagal balance, and baroreflex sensitivity in hypertensive patients. Am J Clin Nutr.

[211] Stewart JM, Ocon AJ, Medow MS (2011). Ascorbate improves circulation in postural tachycardia syndrome. Am J Physiol Heart Circ Physiol.

[212] Ozemek C, Hildreth KL, Groves DW, Moreau KL (2016). Acute ascorbic acid infusion increases left ventricular diastolic function in postmenopausal women. Maturitas.

[213] Bassenge E, Fink N, Skatchkov M, Fink B (1998). Dietary supplement with vitamin C prevents nitrate tolerance. J Clin Invest.

[214] Watanabe H, Kakihana M, Ohtsuka S, Sugishita Y (1998). Randomized, double-blind, placebo-controlled study of the preventive effect of supplemental oral vitamin C on attenuation of development of nitrate tolerance. J Am Coll Cardiol.

[215] Watanabe H, Kakihana M, Ohtsuka S, Sugishita Y (1998). Randomized, double-blind, placebo-controlled study of ascorbate on the preventive effect of nitrate tolerance in patients with congestive heart failure. Circulation.

[216] Satawiriya M, Khongphatthanayothin A, Limsuwan A (2024). Reversible severe pulmonary hypertension related to scurvy in children. BMC Cardiovasc Disord.

