# Supplementary material for: Vitamin C deficiency can lead to pulmonary hypertension: a systematic review of case reports
Source: BMC Pulm Med. 2024 Mar 19;24:140. doi: 10.1186/s12890-024-02941-x (PMC10949735; doi:10.1186/s12890-024-02941-x)
Supplement: Supplementary file 1 — Supplementary Material 1. [file 12890_2024_2941_MOESM1_ESM.pdf]

Vitamin C deficiency can lead to pulmonary hypertension: a systematic review of case reports

Harri Hemilä, MD, PhD [1)], Angelique M.E. de Man, MD, PhD [2)]

1. Department of Public Health, University of Helsinki, POB 41, Helsinki, Finland

2. Department of Intensive Care Medicine, Amsterdam University Medical Centers, location VUmc, Amsterdam, The Netherlands

harri.hemila@helsinki.fi
https://orcid.org/0000-0002-4710-307X

ame.deman@amsterdamumc.nl
https://orcid.org/0000-0001-8738-8295

SUPPLEMENT 1

2024-2-21

This is supplement to a paper submitted to
BMC Pulmonary Medicine
https://bmcpulmmed.biomedcentral.com

Contents **Page**

Search for case reports on scurvy and pulmonary hypertension 2

Fig. S1: Heart rate, respiratory rate, and blood pressure by the age of the patient 3

Fig. S2: Reported improvement in cardiac outcomes over two weeks after the start of vitamin C 4

Fig. S3: Association of scurvy with TAPSE and the diameter of inferior vena cava 5

Fig. S4: Effect of vitamin C on cardiac outcomes in pulmonary hypertension patients 6

Abbreviations used in the description of the 32 cases 7

A case report published after the completion of the systematic review 8

Description of the 32 included case reports 9

Search for case reports on scurvy and pulmonary hypertension.

PubMed

On 2023-7-9, we carried out a search using the following search terms:

("pulmonary hypertens*" or "pulmonary arterial hypertension") and ("vitamin c" or ascorb*)

This gave 46 records

Scopus

On 2023-7-9. we carried out the following search:

(TITLE-ABS-KEY ("pulmonary hypertens*" OR "pulmonary arterial hypertension" OR "right heart failure") AND TITLE-ABS-KEY ("vitamin c" OR ascorb*))

This gave 178 records

Web of Science (Clarivate)

On 2023-5-13, we looked at the citations for the major identified case reports and carried out a search for citing publications. The reports used in this search were the following:

Dean T (2019) [82],

Duvall MG (2013) [83],

Frank BS (2019) [85],

Gayen SK (2020) [86],

Ghulam Ali S (2018) [87],

Ichiyanagi S (2019) [89],

Kupari M (2012) [90],

Mehta CL (1996) [92],

Mertens MT (2011) [93],

Penn EH (2019) [96].

This search gave 94 records.

We also read all the reference lists of the identified case reports.

Together, these approached gave us 32 case reports about scurvy and pulmonary hypertension.

Fig. S1. Heart rate, respiratory rate, and blood pressure by the age of the patient. Data were not available for all cases. For the age range <18 years, the blue continuous line indicates the upper limit, and the red dash line indicates the lower limit for the the estimated 90% coverage of normal population [106]. See extraction of data in Supplements 1 and 2.

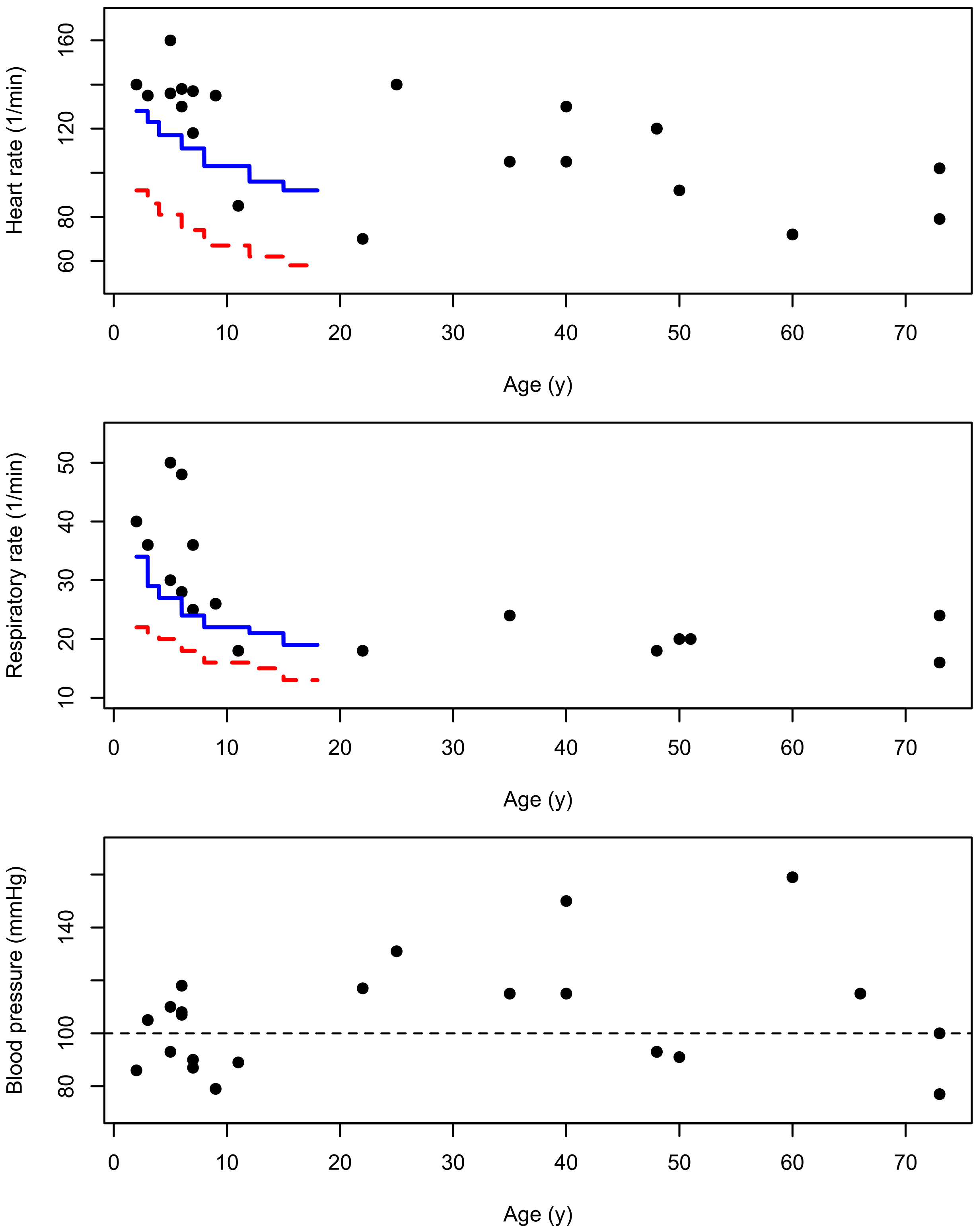

Fig. S2. Reported improvement in cardiac outcomes over two weeks after the start of vitamin C. In the ordinary hospital context, there is no regular daily follow-up by echocardiography or other examinations. Instead, repeat examinations are usually carried out after fixed time periods. Therefore, this recovery curve is biased towards delayed documented recovery. Nevertheless, this analysis indicates that about half of the cases demonstrated cardiac improvement within 2 weeks. The cardiac outcomes used in this figure are heterogeneous, including measures from echocardiography and catheterization, see Supplements 1 and 2. Time point 0 indicates the start of vitamin C administration.

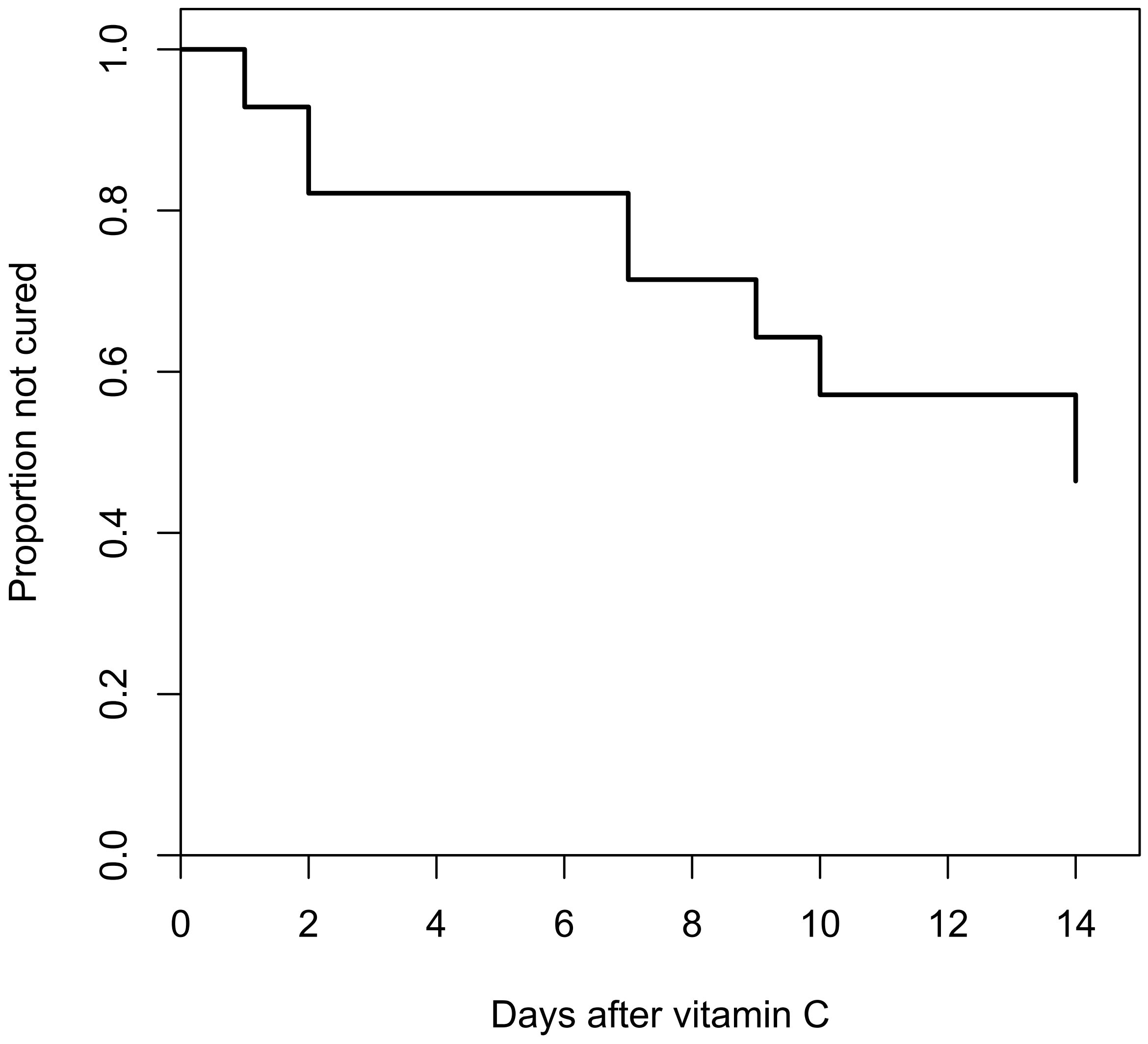

Fig. S3. Association of scurvy with TAPSE and the diameter of inferior vena cava.

A 25-year-old female in the USA [91]. Four months before admission to hospital, TAPSE was 23 mm, and when scurvy was diagnosed, it was 9.5 mm (filled circles on left). There are no TAPSE data after vitamin C was started. Inferior vena cava was 23 mm when scurvy was diagnosed, and 14 mm after vitamin C was administered (open triangles on right). The scale is different on the left-hand side. Period of scurvy is around day 0, which is the day of admission to hospital. The day when vitamin C was started was not published.

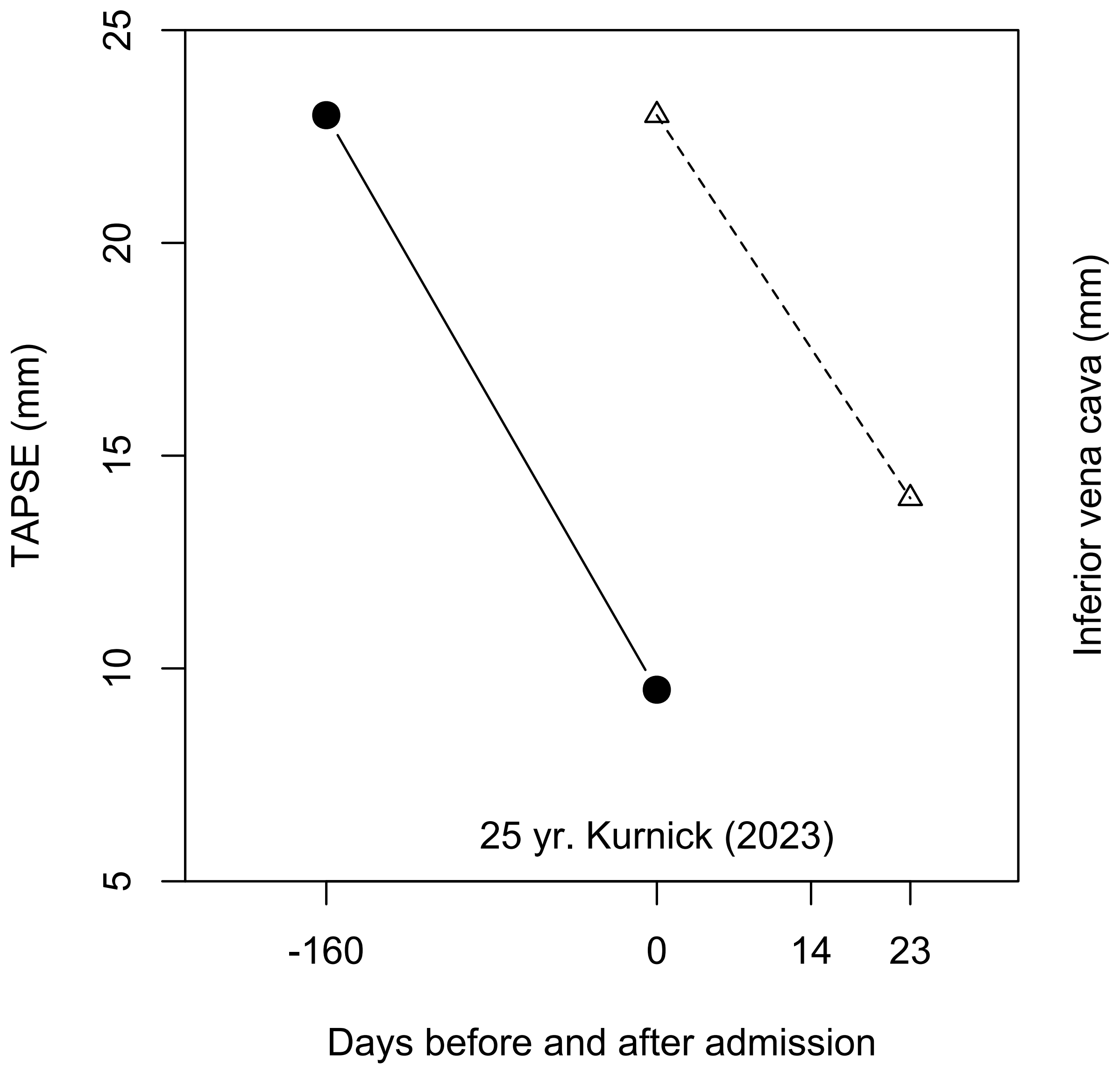

Fig. S4. Effect of vitamin C on cardiac outcomes in pulmonary hypertension patients.

A. a 40-year-old female in Finland [90]. The RV + RV area with filled circles on the left-hand side, and diameter of inferior vena cava with open triangles on the right-hand side.

B. a 6-year-old male in the USA [98]. The RV systolic to diastolic duration ratio with filled circles on the left-hand side, and RV area change with open triangles on the right-hand side.

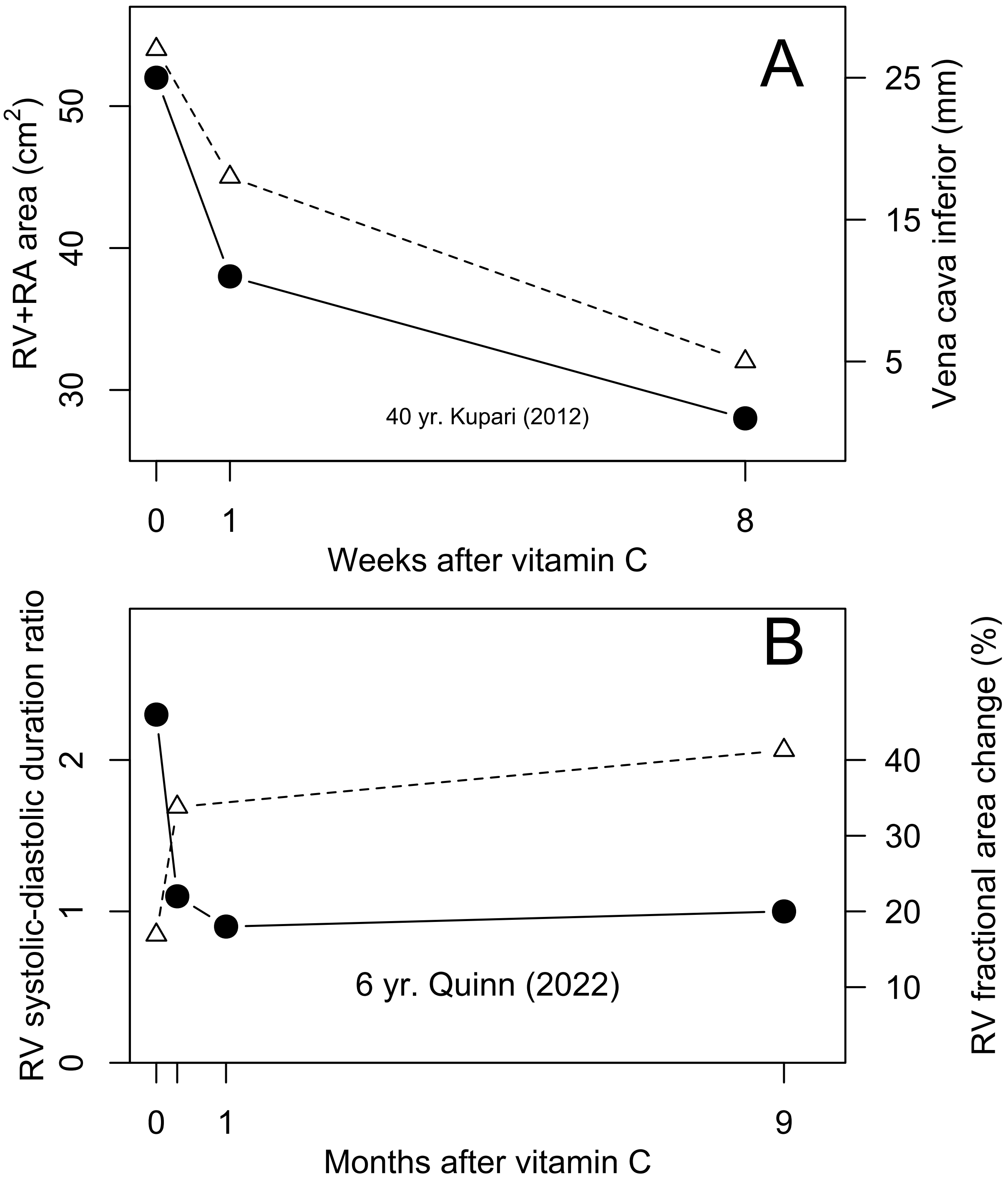

Abbreviations used in this Supplement

ALP, alkaline phosphatase
ALT, alanine transferase
ASD, autism spectrum disorder
AST, aspartate transferase
BNP, brain natriuretic peptide
BP, blood pressure
CT, computed tomography
CTA, CT angiography
CXR, chest X-ray
DLCO, diffusing capacity for carbon monoxide
DVT, deep vein thrombosis
ECG, electrocardiogram
ECHO, transthoracic echocardiogram
ESR, erythrocyte sedimentation rate
FEV_1, forced expiratory volume in 1 second
FVC, forced vital capacity
Hb, hemoglobin
HD, hospital day
HR, heart rate
ICU, intensive care unit
INR, internalized normalized ratio (measure of blood clotting and liver function)
LV, left ventricle/ventricular
LVEF, left ventricular ejection fraction
mPAP, pulmonary artery mean pressure (can be estimated from sPAP: $mPAP = 0.61 \times sPAP + 10$)
PAH, pulmonary artery hypertension
PAP, pulmonary artery pressure; mPAP, mean PAP, sPAP, systolic PAP
sPAP, pulmonary artery systolic pressure (can be estimated from TRPG: $sPAP = TRPG + 10$)
PH, pulmonary hypertension
RA, right atrium/atrial
RBBB, right bundle branch block
RHF, right heart failure
RV, right ventricle/ventricular
SpO2, oxygen saturation level
SR, sinus rhythm
TAPSE, tricuspid annular plane systolic excursion index
TR, tricuspid regurgitation
TRV, TR velocity (m/s)
TRPG, tricuspid regurgitation pressure gradient ($= 4 \times TRV^2$; used to estimate sPAP)

A case report published after the completion of the systematic review

After we received the reviewer reports, we noted a new report (2024) on two cases of PH induced by scurvy [1]. The two cases are consistent with the set we included in our analysis, but we did not append them to our analysis. This is a brief summary of the cases.

Case 1 was a 2-year-old boy presented to hospital with vomiting, diarrhea, and fever. He had hypotension and was transferred to the pediatric intensive care unit where he suddenly collapsed and had confirmed cardiac arrest. The mPAP was 49 mmHg, based on peak TRV of 4.1 m/s. Three days after referral, the right cardiac catheterization showed mPAP 20 mmHg. Vitamin C level was undetectable. The boy was administered 0.3 g/day vitamin C. After he was discharged from the hospital, his symptoms gradually improved and returned to his usual state of health within 2 weeks. At the one-year follow-up, he has been well with normal PAP. Prior to the episode, the boy had restrictive eating patterns. His dietary preference were rice, pork, eggs, and milk, with very few vegetables and fruits.

Case 2 was a 6-year-old boy with ASD. He had progressive dyspnea and low-grade fever for 1 month and refused to walk due to his swollen, painful left knee. Chest x-ray revealed mild cardiomegaly with prominent pulmonary artery trunk. Echocardiography showed enlargement of RA, RV, and main pulmonary artery and mPAP was 36 mmHg (peak TRV of 3.4 m/s). Right cardiac catheterization found mPAP 50 mmHg. His dietary intake consisted mainly of rice and egg with avoidance of milk, vegetables, and fruit. Vitamin C level was undetectable. With a dose of 0.3 g/day vitamin C, his symptoms improved gradually in 2 weeks, and he could walk in 1 month. His 2-year echocardiographic follow-up was normal without signs of PH.

1. Satawiriya M, Khongphatthanayothin A, Limsuwan A.
Reversible severe pulmonary hypertension related to scurvy in children.
BMC Cardiovasc Disord. 2024;24(1):24.
https://doi.org/10.1186/s12872-023-03629-6
https://www.ncbi.nlm.nih.gov/pmc/articles/PMC10765653

Description of the 32 included case reports

**Abbas (2016) [76]**	https://pubmed.ncbi.nlm.nih.gov/27682441
Patient	50 y woman, previously healthy. Never smoked, drank alcohol, or used recreational drugs. No current medications.
Duration of the symptoms before hospital	3 months
Signs and symptoms	Progressively worsening shortness of breath associated with extreme fatigue, anorexia, about 5 kg weight loss, yellowing of eyes, heavy menstrual bleeding, and a lower extremity rash.
Main findings	Neck veins were distended to her earlobes when sitting. Massive hepatomegaly.<br>A nonconfluent, nonblanching petechial rash on anterior legs, and a large ecchymosis on her thighs and buttocks.<br><br>HR 92/min, BP 91/56 mmHg, respiratory rate 20/min. SpO_2 85% on room air.<br><br>ECG showed SR with low voltage in the anterioinferior leads.<br><br>CT showed periportal edema with hepato-splenomegaly, enlarged and inferior vena cava, portal vein, and splenic vein.<br><br>**ECHO** showed enlarged RA and RV, septal flattening, right ventricular moderate hypokinesis, and mild-to-moderate TR without systolic reversal in the hepatic vein. No evidence of LV dysfunction.<br><br>Hb 34 g/L, MCV 90 fL, Fe 90 ug/L, ferritin 147 ng/mL<br>AST 726 U/L, ALT 670 U/L, ALP 154 U/L, INR 2.2<br>BNP 347 pg/mL (normal <100). Viral hepatitis: negative.<br><br>B1 and folate normal<br><br>Vitamin C plasma level 1 mg/L (5.7 µM)
Drugs in hospital	None
Other vitamins and minerals	
Diet before the event	Diet at home was strictly limited to cereal, eggs, and milk.
Vitamin C treatment	Vitamin C dose and route not reported.
Effect of vitamin C	Symptoms improved rapidly and remarkably. Heavy menstrual bleeding improved and liver enzymes normalized.<br>At 4 weeks, **ECHO** was completely normalized with no evidence of RV dysfunction and normal PAP.
NOTES	To ask for further details of the case, we contacted Farrukh Abbas by email on 2023-2-8 and 2023-2-23 but did not get a response.

**Abe (2021) [77]**<br>**Marissa Fakaosita**	https://pubmed.ncbi.nlm.nih.gov/34033530
Patient	7 y boy with ASD
Duration of the symptoms before hospital	1 month
Signs and symptoms	Pain in the right hip and need for wheelchair. Pain spread to several joints over the next 2 weeks.<br>Impaired walking owing to polyarthralgia, fatigue.
Main findings	Light perifollicular petechiae on the lower legs. Pain was elicited in both thighs and lower legs with deep palpation which seemed to impair mobility.<br><br>HR 137/min, BP 87/47 mmHg, respiratory rate of 25/min, SpO_2 of 100% in room air.<br><br>ECG showed sinus tachycardia, an incomplete RBBB and nonspecific T-wave abnormalities.<br><br>Chest contrast-enhanced CT showed normal-sized pulmonary arteries with enlargement of the RA and RV and evidence of concurrent moderate right and small left pulmonary effusion with no anomalous pulmonary venous return or embolism.<br><br>**ECHO** showed RHF with evidence of PH. The RA and RV were enlarged and TAPSE was 8 mm (normal ≥17). The interventricular septum was flattened (D-shaped), with sPAP 72 mmHg estimated by TR).<br>LV function was normal.<br><br>Hb 72 g/L, Fe content and saturation decreased, ferritin normal, but no data<br>CRP 14.7 mg/L (normal <5)<br>ESR 45 mm/h (<10).<br><br>Vitamins A, B1, B12, D, Zn, in normal range, but no data<br><br>Vitamin C level undetectable.
Drugs in hospital	None
Other vitamins and minerals	In addition to vitamin C, diet was supplemented with vitamin C-rich foods and PediaSure 1.5 Cal (see https://abbottnutrition.com/pediasure-1_5-cal).<br>The doses were low and we classify vitamin C alone.
Diet before the event	Extremely nutrient-selective, eating mainly carbohydrates but no fruit or multivitamin supplements. The last time he ate meat, fish or vegetables was more than 2 yr before presentation.
Vitamin C treatment	Vitamin C 0.15 g/d for 5 days intravenously.

Effect of vitamin C	There was rapid resolution of symptoms with vitamin C administration (“improved progressively thereafter”).<br>At follow-up he was able to walk and run without difficulty or pain.<br><br>A follow-up **ECHO** showed complete resolution of the RHF and PH. The RV dilation was completely resolved and the estimated sPAP 33 mmHg by TRV (normal <35).
NOTES	We were able to contact Marissa Fakaosita in 2023-5-3 and 2023-5-4, with the following response: “the patient did see a cardiologist in follow up that I don't have access to see the echo readings. I can only see the cardiologist note that the echo was normal with pulm HTN completely resolved. The dilated R atrium and R ventricle had also resolved. There are no other echos, as he was discharged from cardiology (since underlying issue was resolved)” … “Follow up echo was 38 days after first Vit C administration”

**Azar (2019) [78]**	https://doi.org/10.12691/ajmcr-7-5-2
Patient	73 y African American female. History of morbid obesity with bariatric surgery 10 y earlier. Subsequently 80 kg weight loss. Right hip replacement. 30-pack-year smoking. No regular medications.
Duration of the symptoms before hospital	2 months
Signs and symptoms	Increasing shortness of breath, which progressed to dyspnea at rest. Abdominal distension for 2 weeks. Bilateral lower limb swelling and spontaneous ecchymosis on lower limbs.
Main findings	Hepatomegaly. Jugular non-pulsatile venous distension up to the base of the neck, prolonged expiration with bilateral diminished air entry. Bilateral pitting lower limb edema to mid-tibial shaft, and scattered ecchymosis bilaterally.<br><br>HR 102/min, BP 77/56 mmHg, respiratory rate of 24/min. SpO_2 of 84% on room air, and 94% with 4 L/min O2.<br><br>Polysomnography 2 months earlier, no obstructive sleeping aphnea.<br><br>Abdominal ultrasound excluded cirrhosis and portal hypertension. Liver enzymes normal<br><br>Cardiac exam showed right ventricular heave, regular heart sounds, loud P2, pan-systolic murmur, S3 left to the xiphoid process.<br><br>CXR showed cardiomegaly.<br><br>ECG showed SR 102 bpm, right axis deviation, poor R-wave progression, RV hypertrophy, and RBBB. RV strain, including ST depression and T-wave inversion in V2-V5, RA dilation, manifested by high amplitude P wave (P pulmonale) in limb lead 2.<br><br>Pulmonary function tests showed a mild decline in DLCO, ruling out significant lung disease.<br><br>Ventilation/perfusion scan excluded pulmonary embolism.<br><br>**ECHO** showed significant signs of LV concentric hypertrophy, with normal systolic and diastolic function, and LVEF 75%. Increased RV wall thickness, and severe RV dilation with moderately reduced function, flattening of the interventricular septum. Moderate dilation of RA, severe TR, with jet 5.14 m/s. The pressure gradient between the RV and RA was 105 mmHg, and sPAP 112 mmHg.<br><br>Right heart **catheterization:**<br>mPAP 50 mmHg<br>PVR 6.7 Wood units.<br>Wedge pressure 9 mmHg.<br>cardiac index of 2.76 L/min/m^2.

	Vitamin C level undetectable.
Drugs in hospital	Furosemide<br>Spironolactone<br>Sildenafil, no clinical improvement<br>Inotropics, no clinical improvement
Other vitamins and minerals	
Diet before the event	Diet significantly unbalanced, lacking fruits and vegetables for the last 2 y. Poor compliance to vitamins and supplements prescribed after her bariatric surgery.
Vitamin C treatment	**5 days after admission, died before scurvy was diagnosed and treated.**
NOTES	To ask for further details of the case, we contacted Dr. Azar by email on 2023-5-23 and 2023-6-13 but did not get a response.

**Azar-2 (2023) [79]**	https://pubmed.ncbi.nlm.nih.gov/37011995
Patient	Female in her 30s. Significant obesity, major depression and generalized anxiety disorder
Duration of the symptoms before hospital	8 weeks
Signs and symptoms	Progressive exertional dyspnea, bilateral lower limb swelling.
Main findings	Bilateral ecchymotic lesions involving the four extremities, mainly the lower limbs, bilateral lower limb pitting oedema, corkscrew hair.<br><br>Jugular venous distension, regular rapid heart sounds, a loud P2 and a pansystolic murmur heard best left of the xiphoid process.<br><br>CXR: mild bilateral pleural effusion and cardiomegaly.<br><br>ECG sinus tachycardia at 112 bpm, right axis deviation and RV hypertrophy, P pulmonale in limb lead 2.<br><br>Pulmonary function tests no obstruction with FEV1/FVC >70% predicted, FVC 83% predicted. Diffusion capacity for CO reduced to 61% predicted.<br><br>**ECHO** showed LVEF 60%, normal LV functions, moderately dilated RV, dilated RA, moderately reduced RV systolic function, severe TR, estimated sPASP 56 mm Hg, flattening of the interventricular septum positive for D-sign.<br>Obstructive sleep apnea was excluded by polysomnography a few months prior to her admission.<br><br>HR chest no signs of interstitial lung disease; V/Q scan no mismatched defects, excluding chronic thromboembolic PH.<br><br>**Catheterization** mPAP 41 mmHg, wedge pressure 11 mmHg, PVR 6 WU. Cardiac index of 2.49 L/min/m^2, CO 5 L/min.<br><br>Ascorbic was undetectable.
Drugs in hospital	Tadalafil and diuretics.
Other vitamins and minerals	
Diet before the event	Diet was significantly unbalanced, had been lacking fruits and vegetables for the previous few years.
Vitamin C treatment	“Vitamin C supplementation” may indicate oral route, but not expolicitly stated, also dose is unclear
Effect of vitamin C	A few days later, she showed signs of clinical improvement with stabilisation of her vital signs, and she was successfully weaned from oxygen and transferred to the regular nursing floor.<br><br>3 wk later, a repeat **ECHO** showed normal RV size and systolic function, with no further findings suggesting PH.<br>She had resolution of petechiae and dyspnoea. She continued oral

	vitamin C supplementation.<br><br>3 months, ECHO showed normal left and right chambers size, normal EF, normal RV systolic function and no TR or signs of PH.<br>The patient was counselled to continue taking vitamin C supplementation.
NOTES	To ask for further details of the case, we contacted Dr. Azar by email on 2023-5-23 and 2023-6-13 but did not get a response.

**Benhamed (2019) [80]**	https://doi.org/10.3166/afmu-2019-0169
Patient	40 y male. Rheumatoid purpura in childhood.
Duration of the symptoms before hospital	10 days.
Signs and symptoms	Worsening exertional dyspnea. Asthenia, epistaxis.
Main findings	HR 130/min, BP 150/81 mmHg. SpO2 of 86% on room air. No signs of heart failure.<br><br>ECG SR, no abnormalities.<br>Angiography excluded pulmonary embolism.<br><br>**ECHO** revealed RV dilatation, paradoxical septum. sPAP 100 mmHg.<br><br>Laboratory results:<br>Arterial blood gass pH 7.55, $PaCO_2$ 21 mmHg, PaO_2 58 mmHg, lactate 3.7 mmol/l<br><br>D-dimer 643 µg/L<br><br>Vitamin C level undetectable (<5 µM).<br>In addition, vitamin K deficiency.
Drugs in hospital	Diuretic therapy (not specified) “Après traitement diurétique”
Other vitamins and minerals	
Diet before the event	Food allergies. Exclusively dairy products for several years.
Vitamin C treatment	Vitamin C dose and route not reported.<br>Authors write “From a therapeutic point of view, daily administration of vitamin C (1 g/d), divided into several doses daily for 15 days, remains the historical reference” [our translation], but they do not state explicitly that 1 g/day was used for their patient.
Effect of vitamin C	Day 2 sPAPs 70 mmHg and amelioration of symptoms.<br><br>Day 7 **ECHO** sPAP 28 mmHg.<br>“After diuretic treatment and vitamin C supplementation, the patient presented a marked improvement in symptoms, including a partial regression of purpuric lesions and PAH (PAPs measured at 70 mmHg at 48 hours). On the seventh day management, ultrasound control in cardiology measured PAPs at 28 mmHg” [our translation].
Time series	In their paper, authors reported [free translation]:<br><br>[baseline sPAP during scurvy] “systolic pulmonary arterial pressure (sPAP) measured at 100 mmHg… a marked improvement in symptoms, including partial regression of purpuric lesions and PAH (sPAP was 70 mmHg at 48 hours). On the seventh day of treatment, a repeat cardiologic ultrasound measured sPAP of 28 mmHg.”

	These figures are shown in our graph.
NOTES	To ask for further details of the case, we contacted Dr. Benhamed by email on 2023-5-13 and 2023-5-23 but did not get a response.

**Conte (2021) [81] Lauren Ann Weber**	https://pubmed.ncbi.nlm.nih.gov/33960312
Patient	48 y female. Generalized anxiety and interstitial cystitis. Recently liver injury secondary to polypharmacy.
Duration of the symptoms before hospital	2 weeks
Signs and symptoms	Rest and exertional dyspnea. Lower extremity edema, early satiety, increased abdominal girth.
Main findings	Anasarca.<br><br>New systolic and diastolic cardiac murmurs.<br><br>NT-proBNP elevated, no data<br>Cardiac troponins normal, no data<br><br>CT angiography and invasive coronary angiography excluded pulmonary embolism and coronary artery disease.<br><br>**ECHO** revealed normal LV systolic function, biatrial enlargement, moderate to severe mitral regurgitation, moderate aortic insufficiency, severe TR, decreased RV function, and moderate to severe PH. The estimated sPAP was 60 mmHg. Microbubble study was negative for intracardiac shunting.<br><br>Right heart **catheterization** demonstrated elevated left- and right-sided pressures, with an RA pressure of 20 mmHg, mPAP 35 mmHg, mean pulmonary wedge pressure 16 mmHg, and cardiac index of 2.1 L/min/m^2 using the Fick equation. PVR not reported<br><br>Vitamins B1, B6, B12, D, E, folate, Se, Zn normal (Table 1 shows data)<br><br>Vitamin C level undetectable.
Drugs in hospital	Diuretic therapy (not specified) led to improvement
Other vitamins and minerals	
Diet before the event	Had been avoiding foods containing vitamin C to decrease her interstitial cystitis symptoms as per internet guidance.
Vitamin C treatment	"vitamin C repletion" but dose and route not reported.
Effect of vitamin C	1 month later, hypervolemia and end organ dysfunction. Repeat right heart catheterization demonstrated a cardiac index of 1.5 L/min/m^2 using the Fick equation. Vitamin C levels were at the lower normal limit.<br>The patient was started on milrinone and referred for valve surgery. She underwent aortic and mitral valve replacement and tricuspid valve annuloplasty while continuing vitamin C.
NOTES	We were able to contact Lauren Ann Weber in 2023-2-23, with the following response: "I apologize I don't have the information any longer."

**Dean (2018) [82]**<br>**Paul Kim**	https://pubmed.ncbi.nlm.nih.gov/30351242
Patient	6 y boy.
Duration of the symptoms before hospital	3 months
Signs and symptoms	Bilateral lower extremity pain and refusal to bear weight. Before proceeding with planned bone biopsy, the boy became acutely tachycardic, hypotensive, and pulseless.<br>Returned to spontaneous circulation and a dopamine infusion was started, leading to stable blood pressures.
Main findings	BNP 850 pg/mL (normal <100).<br><br>Post-arrest **ECHO**, performed while on a dopamine infusion and shortly following epinephrine and phenylephrine boluses, demonstrated tachycardia 138 bpm with hyperdynamic LV systolic function (shortening fraction = 61%). Insufficient TR for estimation of RV pressures, but mild septal flattening was suggested.<br><br>A later, retrospective review of the above ECHO demonstrated an abnormal RV/LV diameter ratio of 1.7 [>1 has been associated with increased adverse events in pediatric PH]. LV eccentricity index (LVEI) was abnormal at 1.7 [LVEI > 1 in adult PH suggests impaired RV function]. The pulmonary artery acceleration time (PAAT) in the post-arrest ECHO showed an abnormal value of 90 ms (normal ≥ 100 ms).<br><br>Hospital day 3<br>BNP 2716 pg/mL, and developed poor urine output and peripheral edema.<br><br>Hospital day 5<br>**ECHO** showed significant RV dysfunction with both RA and RV dilation and prominent septal bowing. There was increased TR, with TR jet based pressure gradient 68 mmHg (excluding RA pressure).<br><br>Concern for PH prompted a retrospective review of the original post-arrest ECHO and a more detailed assessment of the RV function. The RV/LV diameter ratio had increased to 2.8, while the LVEI had increased to 2.85. The PAAT was inadequately assessed on HD5, in part because the Doppler flow pattern in the pulmonary artery was low at <50 cm/s (consistent with low RV output).<br><br>Hospital day 8<br>CT arteriogram of the chest, abdomen, and pelvis did not find evidence of pulmonary embolus or vasculitis.<br><br>Hospital day 9<br>**Catheterization** confirmed mild PH.<br>PVR index 3.5 Wood units × m^2 (Table 1)<br>We assume body surface are 0.85 m^2 which gives PVR = 4.1 Wood units.

	Wedge pressure 4 mmHg.<br>mPAP 12-13 on iNO and milrinone.<br><br><u>Hospital day 10</u>,<br>Vitamin C level undetectable, <5 µM.<br>Vitamin A level undetectable.<br><br>Vitamins B1, B3, B6, B12, D, E, Zn normal (Table 2 shows data)
Drugs in hospital	Dopamine after cardiac arrest<br>Epinephrine<br>Phenylephrine<br>iNO on HD5 and symptoms and ECHO improved<br>Milrinone on HD5 and symptoms and ECHO improved<br>Sildenafil on HD10, and subsequently was weaned off iNO and milrinone.
Other vitamins and minerals	Vitamin A 10000 IU/day.
Diet before the event	Diet lacked fruit, vegetables, vitamins, or fortified foods, resulting in multiple vitamin deficiencies.
Vitamin C treatment	HD10: Vitamin C 0.3 g/d intravenously.
Effect of vitamin C	By hospital day 12, BNP had normalized and **ECHO** parameters had nearly normalized (LV/RV diameter ratio of 0.9, LVEI 1.11–1.18, PAAT 140 ms, TR jet based pressure gradient 19 mmHg (excluding RA pressure), mild septal flattening but qualitatively normal RV function).<br><br>At 6 months, **ECHO** demonstrated no evidence of PH, and he was weaned off sildenafil.<br>At 12 months, he remained asymptomatic with a reassuring ECHO.
NOTES	To ask for further details of the case, we contacted Paul Kim by email on 2023-2-8 and 2023-2-23 but did not get a response.

**Duvall (2013) [83]**	https://pubmed.ncbi.nlm.nih.gov/24190688
Patient	9 y boy with ASD.
Duration of the symptoms before hospital	4 months
Signs and symptoms	Developed a limp. Neurologic and orthopedic evaluations did not reveal an etiology. Symptoms worsened despite physical therapy.<br>Finally, entirely unable to ambulate, and developed a dry cough and labored breathing.
Main findings	HR 135/min, BP 79/66 mmHg, respiratory rate of 26/min. SpO_2 of 96% on room air.<br><br>Was given normal saline, without improvement in tachycardia, persistent tachycardia continued 140-170 bpm, respiratory rate up to 56/min, evolving hypoxia required supplemental oxygen by facemask.<br><br>Early laboratory tests<br>BNP 2019 pg/mL (normal <100)<br>CRP 13 mg/L<br>ESR 23 mm/h (normal <20).<br><br>CXR showed diffuse, nodular, ill defined airspace opacities throughout the right lung greater than left lung (consistent with cardiogenic pulmonary edema versus pneumonia) and small right pleural effusion and an enlarged main pulmonary artery.<br><br>ECG showed sinus tachycardia with a left axis deviation, and right heart strain pattern with incomplete RBBB, and nonspecific ST and T-wave changes.<br><br>CT angiogram showed normal pulmonary veins, a dilated main pulmonary artery (3.2 cm), and no filling defects, excluding pulmonary embolism.<br>Lower limb Doppler ultrasound excluded DVT.<br><br>**ECHO** showed a severely dilated RV with mild to moderately depressed systolic function and a dilated RA and pulmonary artery. RV pressures determined by TRV sPAP 65 to 70 mmHg plus the right atrial v-wave, mPAP 45 mmHg, and end-diastolic pressure of 30 mmHg plus the right atrial pressure (not wave) determined from the pulmonary regurgitant velocities.<br><br>Serial **ECHOs** on HD 2, 3, and 4 showed persistent severe elevation of RV pressure as well as severely dilated RV with moderate dysfunction.<br><br>Vitamin B_1 55 nmol/L (normal 70-180)<br>Vitamin B_6 3.5 ng/mL (5-30)<br>Vitamin B_{12} <150 pg/mL (190-778)<br>Vitamin D 8.2 ng/mL (30-80)

	Vitamin C level undetectable, <5 μM.
Drugs in hospital	No PH drugs<br>Antibiotics for suspected pneumonia.
Other vitamins and minerals	Vitamins B1, B_{12} and D were administered with a multivitamin preparation.<br>After 3 weeks, a multivitamin preparation, and calcium and vitamin D were continued.
Diet before the event	For the past 3 y, the boy had eaten chicken nuggets, crackers, cookies, and water. Refused milk, juice, vegetables, and fruits and was not on vitamin supplementation.
Vitamin C treatment	Starting on HD 6, vitamin C was administered intravenously, but dose was not described.
Effect of vitamin C	By HD 11 (5 days vitamin C), respiratory rate had decreased to the normal range.<br><br>By HD 15 (9 days vitamin C), a repeat **ECHO** showed RV pressure less than half systemic pressure by septal position and resolution of RV dilation and normal systolic function.<br>At the time of discharge, at 3 weeks, he was able to ambulate with assistance without pain.<br><br>Over 18 months, vitamin levels remained normal on vitamin supplements, and repeat **ECHO** showed normal RV pressures and function.
NOTES	To ask for further details of the case, we contacted Melody Duvall by email on 2023-2-8 and 2023-2-23 and 2023-3-4 but did not get a response.

**Ferreira (2020) [84]**	https://pubmed.ncbi.nlm.nih.gov/32133311
Patient	51 y male. Paranoid personality disorder, low socioeconomic status.
Duration of the symptoms before hospital	1 month
Signs and symptoms	Dyspnea on minimal exertion, lower limb edema and painful bruises on the lower limbs.
Main findings	Lower limbs presented perifollicular hemorrhage, bilateral pitting edema and painful nodules and bruises.<br><br>Tachycardia, respiratory rate 20/min, SpO_2 of 98%.<br><br>ECG showed ST-segment elevation in leads DII, DIII and AVF associated with inverted T waves in leads V1-V4.<br><br>Chest CT and perfusion/ventilation scan excluded pulmonary embolism. Lower limb Doppler ultrasound excluded DVT.<br><br>**ECHO** showed enlarged RA and RV and RV systolic dysfunction: the difference between the RV area during diastolic and systolic stages (14.5 – 17.5 cm^2) was 15% (normal 30%). Estimated sPAP 61 mmHg with mild TR.<br><br>Cardiac markers normal, but levels not reported.<br>Hb 51 g/l<br>Fe 23 µg/dL (normal 59-158).<br>Folic acid 2.07 ng/mL (>5)<br>Vitamin B_{12} 118 pg/mL (210-980)<br><br>Vitamin C level 0.5 mg/L (2.8 µM).
Drugs in hospital	None
Other vitamins and minerals	
Diet before the event	Patient had been thinking that someone in his house was poisoning his meals, and therefore restricted his intake of food, including fruit and vegetables.
Vitamin C treatment	Vitamin C 1 g/day intravenously was initiated in the hospital.<br>After discharge, oral vitamin C was continued.
Effect of vitamin C	At 16 months, repeat **ECHO** did not show PH, and revealed normal RV function.
NOTES	

**Frank (2019) [85]**	https://pubmed.ncbi.nlm.nih.gov/30738657
Patient	17 y male, who spent hours indoors playing video games.<br>Developmentally normal, thriving adolescent.
Duration of the symptoms before hospital	2 weeks
Signs and symptoms	Leg pain, bruising without trauma, and chest pain.
Main findings	Symmetric pedal edema, bilateral lower extremity tender ecchymosis, petechiae, corkscrew hairs.<br><br>Cardiac exam found loud P2.<br><br>No unusual findings in X-ray of lower extremity and chest, MRI of the lower extremities, pelvis, and abdomen, CT with angiography of the chest, bilateral lower extremity ultrasound, and ECG.<br><br>**ECHO** showed a structurally normal heart with PH with TRV 4.2 m/s.<br><br>NT-proBNP 140 pg/ml<br>BNP <15 ng/L<br><br>Hb 111 g/L<br>MCV 76 fL<br>Fe 25 ug/dL<br>Ferritin 76 ng/mL<br><br>CRP 57 mg/L<br>ESR 19 mm/h<br><br>Vitamin D 17.8 ng/mL<br><br>Vitamin C level undetectable.
Drugs in hospital	None
Other vitamins and minerals	Vitamin D<br>Fe
Diet before the event	Diet devoid of fruits and vegetables.
Vitamin C treatment	Vitamin C supplement dose was not reported; term supplement suggests oral administration.
Effect of vitamin C	After 2 days, symptoms improved and he was discharged home.<br><br>After 1 month, TRV 3.1 m/s and the symptoms had resolved.<br><br>After 6 months, TRV 2.4 m/s, leg hairs had straightened, and was clinically well.
Time series	"An echocardiogram … (tricuspid regurgitation [TR] velocity of 4.2 m/s).<br>... treated with … ascorbic acid …<br>One month later, his TR peak velocity was 3.1 m/s and the symptoms had resolved.

	After 6 months, his TR peak velocity had normalized (2.4 m/s)”<br><br>These figures are shown in our graph.
NOTES	We were able to contact Benjamin Frank, who responded 2023-2-9 to our question about vitamin C dose and route as follows:<br><br>“vitamin C supplement was definitely given orally, I don't remember the dose...”<br><br>Same day further about dose and echo data:<br><br>“It would depend on finding the records to get the dose. The patient presented 5 or 6 years ago so I don’t think memory will hold.<br><br>We did not do a heart cath in either case so the diagnosis of PAH was empiric based on the data we had and what is known about vit c effect on the lung.”

**Gayen (2020) [86]**	https://pubmed.ncbi.nlm.nih.gov/32033656
Patient	A man in his 60s with a history of hypertension.
Duration of the symptoms before hospital	4 months
Signs and symptoms	Increasing exertional dyspnea, lower extremity swelling, rash along with joint pain in his hips, knees, and feet.
Main findings	Bilateral symmetrical pitting edema; hair loss on extremities.<br>A diffuse, petechial lower extremity rash bilaterally from mid-calf down.<br><br>Increased P2 on cardiac auscultation, no right ventricular heave, no jugular venous distention.<br><br>HR 72/min, BP 159/98, SpO2 99%.<br><br>**ECHO** showed a mildly dilated RV and an increased estimated sPAP 76 mmHg.<br><br>Right heart **catheterization:**<br>mPAP 50 41 mmHg (range 72/22 mmHg)<br>PVR 8.7 Wood units (694 dyn×s×cm^{-5}).<br>Wedge pressure 11 mmHg.<br><br>Hb 84 g/L<br>Fe 22 µg/dL (normal 49-181).<br>Mild deficiency of vitamin D.<br><br>Vitamin C level unmeasurable, < 0.1 mg/dL.
Drugs in hospital	None
Other vitamins and minerals	Vitamin D
Diet before the event	Diet consisted mainly of candy and sports drinks.
Vitamin C treatment	Vitamin C 2 g/d was prescribed, indicating oral administration.
Effect of vitamin C	In 5 months, lower extremity rash and swelling and dyspnea improved. Hemoglobin increased to 113 g/L.<br><br>Repeat **ECHO** showed normal RV size and function with a drop in estimated sPAP to 29 mmHg.
NOTES	

**Ghulam Ali (2018) [87]**	https://pubmed.ncbi.nlm.nih.gov/30582091
Patient	66 y man. Celiac disease and lactose intolerance. No smoking history.
Duration of the symptoms before hospital	well until 4 months prior to<br>… (first visit) discharged<br>… 6 months later (second visit) discharged<br>… 3 months later (third visit)… which led to diagnosis of PH.<br>Thus, in total symptoms lasted for 13 months before the recognition of scurvy and PH
Signs and symptoms	Increasing dyspnea, tachycardia, hypotension, asthenia.<br>Dyspnea and spontaneous onset of ecchymosis in the abdomen (before the 3rd visit).
Main findings	<u>1st visit</u><br>BP 115/70 mmHg, SpO_2 of 91%.<br><br>BNP 298 pg/mL (normal <100).<br>D-dimer 878 ng/mL.<br><br>ECG showed SR and mild ST-segment depression with inverted T waves in leads V1-V4.<br>CXR normal.<br>Lower limb Doppler ultrasound excluded DVT.<br>In spirometry, a moderate depression of DLCO.<br>Chest CT and ventilation/perfusion scan excluded pulmonary embolism.<br><br>Cardiac MRI showed RV hypertrophy and dilatation and hypokinesis of the RV free wall.<br><br>**ECHO** showed RV dilation and dysfunction; TAPSE 16 mm, fractional area change 25%.<br>Moderate TR and severe PH, sPAP 97 mmHg.<br><br><br>Diuretics and oral anticoagulation were started with temporary clinical improvement associated with near normalization of RV overload (sPAP 30 mmHg).<br><br><u>2nd visit 6 months later</u><br>The patient became increasingly dyspneic and there were spontaneous ecchymosis along the left thigh and diffuse purpura over the upper and lower extremities.<br><br>A new pulmonary CT normal<br>Hb 104 g/L<br><br>**ECHO** showed RV overload and moderate PH (sPAP 46 mmHg).<br><br>Anticoagulation was interrupted with mild clinical improvement and partial reabsorption of leg ecchymosis.<br><br><u>3rd visit 9 months after the 1st visit</u>

	Patient reported dyspnea and spontaneous onset of ecchymosis in the abdomen.<br><br>Ventilation/perfusion scan again excluded pulmonary embolism.<br><br>**ECHO** showed severe RV pressure overload (sPAP 80 mmHg).<br><br>Bosentan was administered, no improvement<br><br>Vitamin C level undetectable.
Drugs in hospital	Diuretics on the 1st visit, temporary improvement<br>Oral anticoagulants on the 1st visit, temporary improvement, interrupted on the 2nd visit.<br>Bosentan on 3rd visit, no improvement
Other vitamins and minerals	
Diet before the event	For the past 8 months he had eaten mostly rice and chicken, with no fresh fruits or vegetables.
Vitamin C treatment	Vitamin C 1 g/day orally.
Effect of vitamin C	In 1 week, relief of dyspnea and normalization of SpO_2.<br><br>After 2 weeks, repeat **ECHO** showed only mild RV dilation and normalization of sPAP (31 mmHg).<br>Over 1 y, the patient felt progressively better, with normal exercise capacity, and repeat **ECHO** confirmed sPAP normalization (28 mmHg).
Time series	p. 279:<br>[-9 months]:<br>"TTE … moderate tricuspid regurgitation and severe pulmonary systolic hypertension (pulmonary artery systolic pressure [PASP] 97 mm Hg"<br>[-3 months]:<br>"Six months later… New TTE showed RV overload and moderate PH (PASP 46 mm Hg)."<br>[Hospital visit to diagnose scurvy]:<br>"3 months later … TTE showed severe RV pressure overload (PASP 80 mm Hg)"<br>[After vitamin C]:<br>"After 2 weeks, TTE showed only mild RV dilation and normalization of PASP (31 mm Hg). …<br>The patient felt progressively better throughout 1-year follow-up … TTE confirmed PASP normalization (28 mm Hg)."<br><br>These figures are shown in our graph.
NOTES	

**Gilmore (2021) [88] Mazen Al-Qadi**	https://doi.org/10.1164/ajrccm-conference.2021.203.1_MeetingAbstracts.A3545
Patient	22 y man with ASD
Duration of the symptoms before hospital	No data
Signs and symptoms	Dyspnea, bilateral lower extremity edema, and syncopal episodes.
Main findings	Petechial hemorrhages, corkscrew hairs, and easy bruising.<br>“Typical findings of right-sided heart failure”<br><br>CTA and ventilation/perfusion scan excluded pulmonary embolism.<br><br>**ECHO** showed elevated sPAP ≥50 mmHg with RV dilatation and dysfunction.<br><br>Vitamin C level undetectable.
Drugs in hospital	None
Other vitamins and minerals	
Diet before the event	No description.
Vitamin C treatment	Vitamin C supplementation, route not defined, dose not reported.
Effect of vitamin C	In 6 weeks, symptoms resolved and repeat **ECHO** showed normalization of sPAP (16 mmHg) and resolution of RV strain with return to normal size and function.
NOTES	We were able to contact Mazen Al-Qadi, see following pages.

Mazen Al-Qadi responded 2023-3-17 to our question about vitamin C dose and route as follows:

Below are the answers to your questions:

Vitamin C was provided at 1000 mg once daily (Orally)

Here's the full ECHO report:

**Left Ventricle**	The left ventricle is normal in size with normal wall thickness.<br>The left ventricular systolic function is normal.<br>The ejection fraction is visually estimated at > 55%.<br>There is normal left ventricular diastolic function.
**Mitral Valve**	The mitral valve leaflets are normal, with normal leaflet mobility.<br>There is no significant mitral regurgitation by color and continuous wave Doppler imaging.
**Left Atrium**	The left atrium is normal in size.
**Aortic Valve**	The aortic valve is trileaflet with normal excursion.<br>There is no significant aortic regurgitation by color and continuous wave Doppler imaging.<br>There is no evidence of a significant transvalvular gradient.<br>- Peak transvalvular velocity: 1.1 m/sec
**Ascending Aorta**	The aorta is normal in size in the visualized segments.
**Pulmonary Artery**	The pulmonary artery is not well visualized.
**Pulmonic Valve**	The pulmonic valve is normal.<br>There is trivial pulmonic regurgitation by color and continuous wave Doppler imaging.<br>There is no evidence of a significant transvalvular gradient.<br>- Peak transvalvular velocity: 0.9 m/sec
**Right Ventricle**	The right ventricle is dilated.<br>The right ventricular systolic function is mildly decreased.<br>- TAPSE: 13.0 mm<br>- TASV: 11.0 cm/sec
**Tricuspid Valve**	The tricuspid valve leaflets are normal, with normal leaflet mobility.<br>There is mild to moderate tricuspid regurgitation by color and continuous wave Doppler imaging.<br>The pulmonary artery systolic pressure is moderately elevated.<br>- Peak tricuspid regurgitant velocity: 3.4 m/sec<br>- Estimated pulmonary artery systolic pressure: 50-55 mmHg
**Right Atrium**	The right atrium is mildly dilated.
**IVC/SVC**	The IVC diameter is <=21 mm with <50% decrease in size with inspiration suggesting mildly elevated right atrial pressure (5-10 mm Hg).
**Pericardium**	There is a trivial pericardial effusion.
**Other Echo Findings**	Prominent coronary sinus suggestive of left-sided SVC.

Our question “Would you have similar data for the repeat TTE: "complete resolution of RV dilation..."”

Mazen Al-Qadi gave further data on 2023-3-19:

Baseline vital signs:

BP 117/75 | Pulse 70 | Temp 36.6 °C (Oral) | Resp 18 | Ht 157.5 cm (5' 2.01") | Wt 85.8 kg (189 lb 2.5 oz) | SpO2 98% | BMI 34.59 kg/m²

The first ECHO showed IVC of 1.6 cm. Repeat ECHO below (3 months after vit c therapy). Unfortunately, no TAPSE provided but RV size and function normal. IVC not well visulaized.

1. Normal left ventricular size and systolic function, ejection fraction 55-60%.
2. Normal right ventricular size and systolic function.
3. No significant valvular abnormalities.

Left Ventricle
The left ventricle is normal in size with normal wall thickness.
The left ventricular systolic function is normal, LVEF is visually estimated at 55-60%.
There is normal left ventricular diastolic function.
The left ventricular ejection fraction was quantified (3D) at 55 %.

Right Ventricle
The right ventricle is normal in size, with normal systolic function.
Right ventricle wall thickness is normal.

Left Atria
The left atrium is upper normal in size.

Right Atria
The right atrium is normal in size.

Aortic Valve
The aortic valve is trileaflet with normal appearing leaflets with normal excursion.
There is no significant aortic regurgitation by color flow and continuous wave Doppler imaging.
There is no evidence of a significant transvalvular gradient.

Pulmonic Valve
The pulmonic valve is normal.
There is no significant pulmonic regurgitation by color flow and continuous wave Doppler imaging.
There is no evidence of a significant transvalvular gradient.

Mitral Valve
The mitral valve leaflets are normal with normal leaflet mobility.
There is no significant mitral valve regurgitation by color flow and continuous wave Doppler imaging.

Tricuspid Valve
The tricuspid valve leaflets are normal, with normal leaflet mobility.
There is trivial tricuspid regurgitation by color flow and continuous wave

Doppler imaging.
No pulmonary hypertension, estimated pulmonary artery systolic pressure is at least 16 mmHg.

Other Findings
Rhythm: Sinus Rhythm.

Pericardium/Pleural
There is no pericardial effusion.

Inferior Vena Cava
The IVC is not well visualized precluding the ability to accurately assess right atrial pressure.

Aorta
The aortic root at the sinus of Valsalva is normal.

Please let me know if you have further questions!

Best,
Mazen

**Ichiyanagi (2019) [89]**	https://pubmed.ncbi.nlm.nih.gov/31567273
Patient	3 y with ASD.
Duration of the symptoms before hospital	No data about duration
Signs and symptoms	General fatigue, shortness of breath.<br>Acute heart failure, decreased deep tendon reflexes, unstable gait.
Main findings	CXR showed cardiac dilation and an enlarged main pulmonary artery.<br><br>**ECHO** showed a dilated RV with a RV/LV basal diameter ratio of 1.3, TRV 4.1 m/s, and interventricular septal flattening.<br><br>Pro-BNP 14,513 pg/mL (normal <125).<br>Vitamin B1 20 ng/mL (21.3–81.9).<br>Vitamin B1 was initiated.<br><br>Hospital day 8<br>HR 135/min, BP 105/93 mmHg, respiratory rate 36/min, SpO2 100% on room air<br>Cardiac **catheterization** was scheduled under general anesthesia. Circulatory collapse occurred in a few minutes, and the procedure was cancelled, and transferred to pediatric ICU.<br><br>Hospital day 19<br>Cardiac **catheterization:**<br>mPAP 77 mmHg (PAP range 98/64 mmHg).<br>PVR index 22.62 Wood unit × m^2.<br>Below: BSA 0.55 m^2 Thus, PVR = 41.1 Woods units<br>Cardiac index 2.98 L/min/m2<br><br>Tadalafil was initiated.<br><br>Hospital day 21<br>The patient developed gingival bleeding and was found to have bilateral humeral fractures on X-ray.<br><br>Vitamin C level undetectable.
Drugs in hospital	Tadalafil
Other vitamins and minerals	Vitamin B1
Diet before the event	For 3 months, only chicken and rice, without fruit and vegetables.
Vitamin C treatment	Vitamin C intravenously 0.5 g/day, see NOTES.
Effect of vitamin C	After 1 day, the hemorrhagic diathesis improved and **ECHO** showed improvement of the RV dilation and TR (RV/LV basal diameter ratio, 1.0; TRV 2.4 m/s).<br>“After initiating vitamin C therapy, the findings of PH on [ECHO] improved the following day and the clinical manifestations (ie, gingival bleeding, general fatigue, shortness of breath) improved within 2 weeks”

<table>
<tr><td></td><td>After 6 weeks, repeat ECHO showed sustained improvement of PH on vitamin C therapy, without tadalafil.

Cardiac catheterization:
mPAP 19 mmHg (PAP range 26/12 mmHg).
PVR 2.32 Wood units.
Cardiac index 4.32 L/min/m2</td></tr>
<tr><td>Time series</td><td>Dr. Ichiyanagi kindly provided us with the data for our time series graph, see below.</td></tr>
<tr><td>NOTES</td><td>We were able to contact Shogo Ichiyanagi, who responded 2023-2-9 to our question about vitamin C dose and route as follows:

What was your dose of vitamin C and was that orally or iv?

“The dose of vitamin C was as follow;
week 1: 250mg twice a day/ i.v.
week 2: 250mg once a day/ i.v.
week 3: 125mg once a day/ i.v.
week 4-6: 40mg twice a day/ p.o.
week 7-: 40mg twice a day/ p.o.”

“We still have the echo data.
The peak velocity of TR after 6 week was 2.0 m/s.”

Shogo Ichiyanagi sent more data on 2023-2-20.

<table>
<tr><th></th><th>pre</th><th>post</th></tr>
<tr><td>TR velocity</td><td>3.7m/sec</td><td>2.1m/sec</td></tr>
<tr><td>TRPG</td><td>56mmHg</td><td>17mmHg</td></tr>
<tr><td>TAPSE</td><td>13.0mm</td><td>17.5mm</td></tr>
<tr><td>e'/a'/s'</td><td>8.3/10.4/10.7</td><td>16.1/10.6/12.3</td></tr>
<tr><td>TMF e/a</td><td>41.4/53.2</td><td>54.6/55.1</td></tr>
<tr><td>ventricular septum</td><td>flat</td><td>round</td></tr>
<tr><td>RV basal diameter/ LV basal diameter</td><td>>1.0</td><td><1.0</td></tr>
<tr><td>eccentrisity index</td><td>not appricable</td><td>not appricable</td></tr>
<tr><td>RV-FAC</td><td>21%</td><td>49%</td></tr>
<tr><td>RVdiameter</td><td>not appricable</td><td>not appricable</td></tr>
<tr><td>RV-Tei index</td><td>0.53</td><td>0.03</td></tr>
<tr><td>RV sys to dia duration ratio</td><td>1.8</td><td>1.1</td></tr>
<tr><td>acceleration time</td><td>40</td><td>130</td></tr>
<tr><td>PR velocity</td><td>not appricable</td><td>not appricable</td></tr>
<tr><td>PA diameter</td><td>16.3</td><td>16.1</td></tr>
<tr><td>RA diameter</td><td>6</td><td>6.1</td></tr>
</table>
</td></tr>
</table>

See further data on the next page.

Ichiyanagi sent further data about ECHO and catheterization on 2023-2-24.

event				(pre echo)	1st. cardiac catheterization (PH crisis)					2nd. cardiac catheterization (Before)	Diagnosed possible scurvy.	Vit.C was initiated
date	12/22	12/24	12/26	12/27	12/28	1/1	1/2	1/4	1/5	1/8	1/10	1/11
hospital day	1	3	5	6	7	11	12	14	15	18	20	21
TR velocity (m/s)	2,6	3,4	4,1	3,7		4,9	4,7	4,4	3,7	4,9	3,8	4,0
TRPG (mmHg)	27	46	67	56		97	87	77	56	97	58	64
TAPSE (mm)				13.0mm								
e'/a'/s'				8.3/10.4/10.7								
TMF e/a				41.4/53.2								
ventricular septum				flat								
RV basal diameter/ LV basal diameter				>1.0								
eccentrisity index				not appricable								
RV-FAC				21 %								
RVdiameter				not appricable								
RV-Tei index				0,53								
RV sys to dia duration ratio				1,8								
acceleration time				40								
PR velocity				not appricable								
PA diameter				16,3								
RA diameter				6								
BSA (m^2)										0,55		
Wedge pressure (FiO2 1.0) (mmHg)										9,5		
Wedge pressure (FiO2 1.0 + NO20ppm) (mmHg)										11		
event									(post echo) Approximately 6 weeks after the start of vit C administration	3rd. cardiac catheterization (After)	ent	
date	1/12	1/13	1/15	1/24	1/31	2/8	2/16	2/22	2/26	2/27	3/2	
hospital day	22	23	25	34	41	49	57	63	67	68	71	
TR velocity (m/s)	2,4	2,1	2,4			2,3	2,6	2,4	2,0			
TRPG (mmHg)	22	18	24			21	27	23	17			
TAPSE (mm)		16,1		19,8	17,3			17,3	17.5mm			
e'/a'/s'		14.0/14.5/15.3		13.0/12.5/15.6	10.7/11.2/15.0	12.2/12.7/16.1		10.9/10.1/13.1	16.1/10.6/12.3			
TMF e/a									54.6/55.1			
ventricular septum									round			
RV basal diameter/ LV basal (	1,0								<1.0			
eccentrisity index									not appricable			
RV-FAC									49 %			
RVdiameter									not appricable			
RV-Tei index									0,03			
RV sys to dia duration ratio									1,1			
acceleration time									130			
PR velocity									not appricable			
PA diameter									16,1			
RA diameter									6,1			
BSA (m^2)										0,56		
Wedge pressure (FiO2 1.0) (mmHg)										9		
Wedge pressure (FiO2 1.0 + NO20ppm) (mmHg)										11		

**Kupari (2012) [90]**	https://pubmed.ncbi.nlm.nih.gov/22796843
Patient	40 y woman with mild asthma and food allergies.
Duration of the symptoms before hospital	18 months
Signs and symptoms	Tender red-bluish nodules, ecchymoses, and palpable purpura on legs 18 months earlier.<br>Acute issue was anemia, increasing breathlessness, and subcutaneous bleeding.
Main findings	There were large subcutaneous hematomas on her legs but she was in no acute distress.<br><br>HR 105 bpm, BP 115/75 mmHg, SpO_2 of 100% on ambient air.<br><br>ECG showed flattening of the T waves in the right precordial leads.<br>CXR normal.<br><br>Oral Fe administration was started.<br>Because of a vague suspicion of vasculitis, treatment with oral prednisone was initiated.<br><br>Within a few days, the patient became increasingly dyspneic and SpO_2 dropped to 77-80% on ambient air.<br>Oxygen was administered.<br><br>**ECHO** showed a dilated and poorly contracting RV, TRV 3.5 m/s, an eccentrically deformed LV, pericardial effusion, and flow from right to left atrium through open foramen ovale.<br>Dilated right side of the heart and severely flattened LV.<br><br>CTA revealed dilatation of the pulmonary artery but no pulmonary embolism.<br>A ventilation-perfusion scan excluded pulmonary embolism.<br><br>Right-sided heart **catheterization**, with the patient breathing room air, revealed severe precapillary PH, RV failure, and a large right-to-left shunt.<br>mPAP 48 mmHg (PAP range 74/36 mmHg).<br>PVR 30 Wood units (2400 dyn/s/cm^5).<br>Wedge pressure 3 mmHg.<br><br>**Echo**:<br>RV+RA 52 cm^2<br>Vena cava inferior max diameter 27 mm<br>LV eccentricity index 2.5<br>TAPSE 7 mm<br><br>Hb 74 g/L.<br>Fe 5.4 µM (normal 9-34).

	Transferrin saturation 10% (17%-52%).<br>Transferrin receptor 7.0 mg/L (1.9-4.4).<br><br>Vitamin C level undetectable, <10 µM.
Drugs in hospital	Prednisolon, rapidly tapering doses after discharge<br>Epoprostenol<br>Sildenafil was initiated, but discontinued 3 weeks later.
Other vitamins and minerals	Fe, discharged with Fe administration
Diet before the event	For several years, diet had been deficient of fruit and vegetables, because of proven and presumed food allergies.
Vitamin C treatment	Vitamin C 1 g/day orally.<br>Sildenafil was discontinued 3 weeks later. Therefore, we classify the treatment as vitamin C alone.
Effect of vitamin C	In 2 days, she experienced relief of dyspnea and normalization of SpO_2.<br><br>After 1 week, control **ECHO** showed no signs of PH.<br><br>After 8 weeks (with 5 weeks off sildenafil), she reported normal exercise capacity and had normal Hb 140 g(L<br>Findings at **catheterization** and **ECHO** showed that the PAP, flow, and resistance were completely normal, as was right-sided heart function.<br><br>**Echo 1 week**:<br>RV+RA 38 cm^2<br>LV eccentricity index 1.0<br>Vena cava inferior max diameter 18 mm<br>TAPSE 20 mm<br><br>**Echo 8 weeks**:<br>RV+RA 28 cm^2<br>LV eccentricity index 1.0<br>Vena cava inferior max diameter 5 mm<br>TAPSE 23 mm<br><br>Cardiac **catheterization 8 weeks:**<br>mPAP 15 mmHg (PAP range 26/9 mmHg).<br>Wedge 6 mmHg<br>PVR 1.3 Wood units (107 $dyn/s/cm^5$)..<br><br>Effect of vitamin C on laboratory values:<br><br>Hb<br>at diagnosis 74 g/L<br>8 weeks: 140 g/L (+89%)
Time series	Data for our graph was published in Table 1, the data are extracted above.
NOTES	

**Kurnick (2023) [91]**	https://pubmed.ncbi.nlm.nih.gov/37650076<br>https://doi.org/10.1016/S0735-1097(23)02958-3 (meeting abstract)
Patient	25 y female, USA<br>History of iron deficiency anaemia secondary to menorrhagia.
Duration of the symptoms before hospital	5 months shortness of breath and generalized weakness. ECHO was "unremarkable" (see below).
Signs and symptoms	2 weeks progressive weakness, shortness of breath, myalgia, arthralgia.
Main findings	On admission, HR 123, BP 131/93 mmHg.<br>O_2 saturation 100% on room air.<br><br>ECG revealed sinus tachycardia with right axis deviation.<br><br>CT angiogram of the chest ruled out pulmonary embolism; but revealed significant right heart strain.<br><br>**ECHO** showed sPAP 71.1 mmHg with severely dilated RV and RA. TAPSE 9.5 mm. Non-collapsible inferior vena cava dilated to 3.0 cm.<br><br>**ECHO** 4 months prior to admission: normal ventricles and papillae, normal size RV. Normal TAPSE 23 mm.<br><br>HD4. cardiogenic shock, HR 45, BP 77/56 mmHg, O_2 saturation 69% on room air.. Two episodes of cardiac arrest with pulseless electrical activity but return of spontaneous circulation.<br><br>HD5. **Catheterization**<br>mPAP 48 mmHg (63/43/48 mmHg)<br>Wedge pressure 13 mmHg.<br>PVR 16<br>Cardiac output 2.2 L/min<br>Cardiac index 1.6 L/min/m^2.<br><br>HD14 **Catheterization**<br>mPAP 39 mmHg<br>Wedge pressure 19 mmHg.<br>PVR 4.3 WU<br>Cardiac output 4.6 L/min<br>Cardiac index 2.8 L/min/m^2.<br><br>On admission<br>Hb 71 g/L<br>pro-BNP 330000 pg/mL<br>mildly elevated troponin<br><br>Vitamin B1 and D level were within normal limits<br><br>HD5 AST 136, ALT 71

Drugs in hospital	Norepinephrine , Dobutamine, Milrinone
Other vitamins and minerals	
Diet before the event	Severely restricted diet lacking fruits and vegetables, mostly consuming chickpeas
Vitamin C treatment	2 g intravenous vitamin C per day.<br>Discharged on oral vitamin C 1 g daily
Effect of vitamin C	HD23 Repeat **ECHO** revealed the resolution of right ventricular dilation with complete resolution of RHF and PH. Collapsible inferior vena cava measuring 14 mm. TRPG 16 mmHg.<br><br>HD23 **Catheterization**<br>PAP 36/9 mmHg [we calculate mPAP = 24 mmHg]<br>Wedge pressure 9 mmHg.<br><br>Repeat ECG showed resolution of right axis deviation.
NOTES	We contacted John Sabu to ask for more details (email 2023-3-15, 2023-9-26 and 2023-9-29). We did not receive any response.<br>We were able to contact Adam Kurnick (email 2023-3-15) but we were unable to get any additional information of the case.<br>The case was first published as an abstract (above), but a final report was published before we finished the manuscript and details were extracted from the report (above)

**Mehta (1996) [92]**	https://pubmed.ncbi.nlm.nih.gov/8607903
Patient	40 y woman with anorexia nervosa. She had been in psychiatric care and was on sertraline. Amenorrheic since the birth of her 5 y child; breastfed this child until 6 months prior to presentation.
Duration of the symptoms before hospital	3 months
Signs and symptoms	Arthralgia, myalgia, weakness, fatigue for 3 months.<br>1 week prior to presentation, shortness of breath and a purpuric rash on her legs.
Main findings	On the lower extremities, there were erythematous tender nodules, diffuse perifollicular purpura, and an ecchymosis. Tenderness on palpation in knees, ankles and feet. Ankles showed moderate synovial swelling, warmth, and pain on palpation.<br><br>Height 157.5 cm, and weight 44 kg, BMI 17.7.<br><br>Heart sounds normal<br><br>Hb 118 g/L.<br>ESR 12 mm/h.<br><br>CXR and CT showed diffuse interstitial prominence and multiple intraparenchymal nodules with size <1 cm.<br>Pulmonary function testing showed DLCO 53% and vital capacity of 70%.<br><br>Bronchoscopic evaluation led to severe bleeding and hypotension, and fluid resuscitation thereafter led to severe anasarca.<br>A lung biopsy showed interstitial edema.<br><br>**ECHO** showed severe PH, with a dilated pulmonary artery, and RA and RV enlargement.<br><br>Right heart **catheterization** showed a PAP 55/35 mmHg.<br><br>Vitamin B 1178 nM (88-192)<br><br>Vitamin C level 2 mg/L (11 µM).
Drugs in hospital	None
Other vitamins and minerals	
Diet before the event	Diet consisted of 2-4 bagels per day.<br>Prolonged breastfeeding might also contribute to the decline in vitamin C level in the mother's body.
Vitamin C treatment	Vitamin C supplementation indicates oral administration, but dose was not reported.<br>In addition, "other supportive therapies" which were not described, but they were not corticosteroids or antibiotics.
Effect of vitamin C	In 1 week, her musculoskeletal, skin, and pulmonary symptoms

	resolved.<br><br>In 19 days, the CXR, **ECHO**, and pulmonary function test results had normalized.
NOTES	

**Mertens (2011) [93]**<br>**Case 3**<br>**Elie Gertner**	https://pubmed.ncbi.nlm.nih.gov/21185063
Patient	74 y female with no significant medical history, no smoking.
Duration of the symptoms before hospital	“Several months”; we translate this to >3 months
Signs and symptoms	Worsening exertional dyspnea.
Main findings	Bilateral erythematous and swollen ankles, and a purpuric rash along her right forearm.<br><br>Cardiac auscultation showed grade 2/6 systolic murmur.<br><br>Right heart **catheterization** showed severe PH with sPAP >80 mmHg.<br><br>A chest CT showed a 1.5-cm consolidation in the left upper lobe.<br><br>Pulmonary function test showed moderate restrictive disease with FVC 65% and FEV_1 63%, and DLCO 46% of predicted.<br><br>Hb 124 g/L<br>ESR 41 mm/h<br><br>Vitamin B6 and Zn levels normal<br>Vitamin B1 slightly low, but no data<br><br>Vitamin C level undetectable, <0.12 mg/dL (<7 µM).
Drugs in hospital	Nifedipine, self-discontinued after 1 week.
Other vitamins and minerals	
Diet before the event	She had a number of delusions about sensitivities to foods and other chemicals and limited her diet to ground beef, goat’s milk, tapioca, spring water, and vitamin B_6 and vitamin A supplements.
Vitamin C treatment	Vitamin C supplementation 1 g/day, but route not defined.<br>Vitamin C alone.
Effect of vitamin C	After 5 months, a repeat **ECHO** showed near complete resolution of her PH with vitamin C supplementation alone and she was asymptomatic for any cardiopulmonary symptoms.
NOTES	To ask for further details of the case, we contacted Elie Gertner by email on 2023-2-8 and 2023-2-23 but did not get a response.

**Nagamatsu (2009) [94] Charlene McEvoy**	https://doi.org/10.1378/chest.136.4_MeetingAbstracts.4S-e
Patient	73 y female. Nonsmoker, nondrinker.
Duration of the symptoms before hospital	3 months
Signs and symptoms	Worsening exertional dyspnea. She could walk no more than 6 m. Exercise tolerance was categorized as NYHA class III. Chest pain (in Power Point [PP] presentation)
Main findings	Multiple dark, pigmented, hyperkeratotic skin lesions with excoriations covering all her extremities and trunk.<br><br>from the PP presentation:<br>HR 79/min, BP 100/59, respiratory rate 16/min, SpO2 97% on 2 L/min.<br><br>from the PP presentation:<br>Heart: 3/6 holosystolic murmur at the left lower sternal border which increases with inspiration. No right ventricular lift and P2 is not loud. No S3. JVP <8 cm H_2O.<br>Extremities: lower extremities edema. Generalized atrophy of the muscles.<br><br>Weight 43kg (BMI 16.4).<br><br>Cardiac auscultation showed grade 3/6 systolic murmur.<br><br>Coronary angiogram showed normal coronary arteries.<br><br>Ventilation/perfusion scan excluded pulmonary embolism.<br>Chest CT scan showed biapical parenchymal scarring with mild emphysematous changes.<br><br>Pulmonary function test showed a mildly restrictive ventilation defect: FVC 65%, FEV_1 63%, FEV_1/FVC ratio 96%, DLCO 46%.<br><br>from the PP presentation:<br>ECG: SR, HR 95, cQT 398 msec, non-specific T wave change<br><br>**ECHO** showed normal LVEF, normal wall motion, moderate TR, and severe PAH.<br>from the PP presentation:<br>– normal LVEF (55-60%).<br>No regional wall motion abnormality.<br>– RV normal in size with systolic function.<br>– Right atrium is mildly enlarged. The intraatrial septum appears to be grossly intact with no obvious color Doppler evidence of a shunt.<br>– Moderate tricuspid regurgitation and severe pulmonary hypertension.<br>– Estimated right ventricular systolic pressure is approximately 82 mmHg plus right atrial pressure.

	Right-side heart **catheterization** showed mPAP 48 mmHg (73/32 mmHg), cardiac index 1.2 L/min, normal pulmonary wedge pressure, without evidence of RHF.<br><br>Vitamin C level undetectable, <0.12 mg/dL (<7 µM).<br><br>from the PP presentation:<br>Vitamin B1 23 nM (87-280)<br>Vitamins B6, B12, folate, Zn normal or above normal
Drugs in hospital	Nifedipine 30 mg/d was initiated, but there is no description how long it was continued.
Other vitamins and minerals	
Diet before the event	The patient believed she was sensitive to multiple foods and could not tolerate any citrus. Very strict diet (in the PP presentation): goat milk and cheese, tapioca, ground beef, yams, squash, spring water (Chippewa Falls)
Vitamin C treatment	Vitamin C supplementation indicates oral administration, but dose was not reported.
Effect of vitamin C	At 3 months, she no longer had dyspnea or exercise limitation and the skin lesions had resolved.<br>Weight had increased to 49 kg (BMI 18.6).<br>Vitamin C level rose above normal range.<br><br>Repeat **ECHO** showed a markedly improved estimated PAP of 35 mmHg above the RA pressure.<br>HH+AM: we assume this PAP refers to TR-based sPAP estimate, and we used that to calculate mPAP estimate.<br><br>Chest CT showed resolution of the parenchymal changes.<br>PFT demonstrated a normalization of lung volumes, though DLCO remained reduced at 45%.
NOTES	We were able to contact Dr. Charlene McEvoy, who responded 2023-2-24 that the data were no more available.<br>However, she had a Power Point presentation which gave some further details, see above.

**Nariai (2022) [95]**	https://pubmed.ncbi.nlm.nih.gov/35396776
Patient	2 y girl with unremarkable medical history.
Duration of the symptoms before hospital	2 months
Signs and symptoms	Weight loss, muscle weakness, difficulty in walking.
Main findings	Joint swelling.<br><br>HR 140 bpm, BP 86/49 mmHg, respiratory rate 40/min,<br>SpO_2 80% under room air, temperature 38.8 °C.<br><br>Hb 70 g/L.<br>BNP 195 pg/mL (normal <19).<br><br>CXR showed prominence of the left second cardiac arch without clear evidence of cardiomegaly or pulmonary edema.<br><br>Contrast-enhanced CT excluded pulmonary embolism.<br><br>**ECHO** showed normal LV size and function. There was dilation of the RA and RV with mild TR, D-shaped LV. TRPG was 75 mmHg.<br><br>Vitamin B1 13 ng/mL (normal 24-66)<br><br>Vitamin C level undetectable, <0.2 µg/mL (<1 µM).
Drugs in hospital	Milrinone was administered for 2 days.
Other vitamins and minerals	Vitamin B1 was administered but discontinued in 10 days.
Diet before the event	She had a history of eating unbalanced meals.<br>For 3 months, only white rice and fermented soybeans.
Vitamin C treatment	Vitamin C 0.84 g/day, "intravenously only on the first day" (see below).<br>Vitamin C was administered for 70 days, whereas thiamine was administered just for 10 days.
Effect of vitamin C	By day 2, TRPG decreased to 57 mmHg,<br>By day 3, TRPG decreased to 35 mmHg.<br>*"The D-shape seen on the short axis of the echocardiogram improved immediately"*<br><br>In 1 month, anemia improved.<br>By day 35, she achieved independent walking.<br><br>In 5 weeks TRPG was normalized to 22 mmHg.<br>Vitamin C was discontinued after 10 weeks.
Time series	Dr. Nariai kindly provided us with the TPRG and TAPSE data for our time series graph, see below.
NOTES	We were able to contact Ryoko Nariai, who responded 2023-2-9 to our question about vitamin C dose and route as follows:

"We gave the patient vitamin C intravenously only on the first day, and orally after the second day."

Thereafter Ryoko Nariai sent more data 2023-2-15

"According to the remaining echo data, the TRPG and TAPSE data were as shown in the table below."

Day	1	2	3	4	5		5 weeks later
TRPG	75	57	35	26	30		22
TAPSE	12.1	14.6	15.6	20.7	19.9		19.7

**Penn (2019) [96] Nicholas Marston**	https://pubmed.ncbi.nlm.nih.gov/31403849
Patient	48 y female, history of recurrent pericarditis. 2-5 cigarettes/day.
Duration of the symptoms before hospital	3 weeks
Signs and symptoms	Progressive chest pain and dyspnea. Her chest pain was pleuritic, nonpositional, similar in character to her previous episodes of pericarditis, but was atypical in that it was constant, severe in intensity, and associated with palpitations and dyspnea at rest. She denied orthopnea, paroxysmal nocturnal dyspnea, fevers, chills, or cough. She reported several days of intermittent lightheadedness without syncope.<br>Longstanding bilateral shoulder and knee pain without joint erythema or swelling.
Main findings	Lethargic, spoke in 2-3 word sentences, used accessory respiratory muscles.<br><br>Trace bilateral pitting edema. Skin examination was notable for discrete, nonblanching, purpuric, circular macules on her bilateral dorsal arms in a perifollicular distribution. There was no rash of the lower extremities.<br><br>HR 120 bpm, BP 93/48 mmHg, respiratory rate 18/min, SpO_2 97% under room air.<br><br>Cardiac examination revealed a regular rhythm, a loud P2, right-sided S3, and a holosystolic murmur that increased with inspiration. Jugular venous pressure 15 cm H_2O and increased with inspiration.<br><br>Hb 117 g/L.<br>NT-proBNP 30178 pg/mL (normal <450).<br>AST 138 U/L, ALT 78 U/L, ALP 171 U/L.<br><br>ECG showed sinus tachycardia, right axis deviation, low voltage, and T wave inversion in leads V2–V5.<br><br>**ECHO** demonstrated normal LV function and severe RV dysfunction, with systolic and diastolic septal flattening. Estimated RV systolic pressure was 84 mm Hg, with severe TR and moderate pulmonic insufficiency.<br><br>Patient was started on dopamine, and was taken to the catheterization laboratory.<br>Right heart **catheterization:**<br>mPAP 41 mmHg (PAP range 57/31 mmHg).<br>PVR 13 Wood units ((PVR) was 1050 dyn×sec/cm^5).<br>Wedge pressure 5 mmHg.<br>Right atrium 16 mmHg, RV 54/19 mmHg.<br>Cardiac index 1,9 L/min/m2<br><br>CTA of the chest demonstrated enlarged central pulmonary artery and no

	evidence of pulmonary embolism or parenchymal lung disease.<br><br>Intravenous epoprostenol and furosemide.<br>Norepinephrine, dobutamine, vasopressin.<br><br>Fe 18 µg/dL (37–145)<br>Transferrin saturation 6% (25–45%).<br><br>HD6: Vitamin C level undetectable, <0.1 mg/L (<0.6 µM).
Drugs in hospital	Dopamine<br>Epoprostenol<br>Furosemide.<br>Norepinephrine<br>Dobutamine<br>Vasopressin<br>Sildenafil
Other vitamins and minerals	Fe<br>Multivitamin
Diet before the event	Diet consisted of mostly coffee, chocolate bars, iceberg lettuce.
Vitamin C treatment	Vitamin C orally, but dose not described.<br>In addition, multivitamin, and intravenous Fe repletion.<br>At 36 hours, inhaled epoprostenol was discontinued and oral sildenafil initiated. Sildenafil was later discontinued, and she remained asymptomatic.
Effect of vitamin C	Within 48 hours of vitamin C, multivitamin, and Fe administration, her symptoms had resolved. Repeat right heart **catheterization** demonstrated a RA pressure 17 mmHg, mPAP 26 mmHg.<br>She was discharged home on vitamin C, multivitamin, and sildenafil.<br><br>6 weeks later repeat **ECHO** demonstrated complete normalization of RV size and function, with estimated RV systolic pressure of 28 mmHg with only trace pulmonic and tricuspid regurgitation.<br>Sildenafil was discontinued, and she has remained asymptomatic.
NOTES	We were able to contact Nicholas Marston by email on 2023-3-7, who responded<br>“Unfortunately, I can't find what the exact dose of Vitamin C she was given. I know it was oral supplementation and she responded quickly. I don't think it was a particularly high dose.”

**Petersen (2019) [97]**	https://shmabstracts.org/abstract/when-life-doesnt-give-you-lemons-a-rare-case-of-acute-heart-failure
Patient	5 y boy with ASD
Duration of the symptoms before hospital	2 months
Signs and symptoms	Fatigue, lower extremity pain, petechial rash, weight loss, Refusal to walk for 1 week.
Main findings	HR 160 bpm, BP 93/68 mmHg, respiratory rate 30/min.<br>SpO_2 normal on ambient air.<br><br>Cardiac auscultation S3 gallop, no jugular venous distension.<br><br>ESR 33 mm/h.<br><br>After intravenous fluids, he became acutely hypotensive and hypoxic necessitating oxygen via a face mask and vasopressor support.<br><br>Lower limb Doppler ultrasound excluded DVT.<br>CTA normal.<br><br>**ECHO** indicated PH, a severely dilated RV with systolic dysfunction, and elevated right-sided pressures.<br><br>Erythrocyte sedimentation rate 33 mm/h<br><br>Vitamin C level undetectable.
Drugs in hospital	Vasopressor
Other vitamins and minerals	
Diet before the event	Extremely restricted diet due to oral aversion, but no further description of the usual diet is described.
Vitamin C treatment	Vitamin C intravenously, but dose not reported.
Effect of vitamin C administration	Immediate improvement in PAP.<br>In 1 week, normalization of strength and gait.<br><br>In 2 weeks, complete reversal of RV dysfunction.
NOTES	We were able to contact Dr. Liz Petersen, who responded 2023-2-26 that the data were no more available.

**Quinn (2022) [98] Benjamin Frank Moore**	https://pubmed.ncbi.nlm.nih.gov/36389378
Patient	6 y boy with ASD
Duration of the symptoms before hospital	3 months
Signs and symptoms	1-week prior to presentation, inability to bear weight on the left leg. Parents brought him to clinic in a stroller.<br>Weight loss 5% in 3 months.
Main findings	During upper endoscopy the patient developed bradycardia, hypotension, cardiac arrest.<br><br>CT excluded pulmonary embolism.<br><br>**Echo** shortly post-arrest showed signs of PAH including elevated TRV and severely diminished RV function, and significant RV hypertrophy, suggesting that PAH predated the cardiac arrest.<br><br>"Other diagnoses on the differential for the cardiac arrest that were excluded included tachyarrhythmia (no evidence on cardiac monitor during the event), cardiac tamponade (no evidence on echocardiogram or clinically), pulmonary embolism (normal CT scan), and underlying primary neurologic or genetic/metabolic disorders. Given this, scurvy-associated PAH was thought to be the primary contributor to his arrest."<br><br>Vitamin B1 55 nM (70–180)<br>Vitamin A 15 µg/dl (19–77)<br>Vitamin D <3.5 ng/ml (30–96l)<br>ferritin 16.6 ng/ml (>20)<br>Fe saturation 12% (>20%)<br>Se 43 ng/mL (70–150)<br>Zn 0.39 µg/mL (0.6–1.2).<br><br>Vitamin C level undetectable, <0.1 mg/dL (<0.6 µM).
Drugs in hospital	iNO on HD9<br>Sildenafil on HD9
Other vitamins and minerals	Vitamin B1 within 24 h of admission to pediatric ICU.<br>Multivitamin within 24 h of admission to pediatric ICU.<br>Se started on HD2.<br>Discharged with multivitamin, Zn, Fe.
Diet before the event	Diet had been restrictive after choking on a French fry at 2 y of age, and consisted of a nutritionally complete supplemental beverage and candy. In 6 months prior to presentation, he further narrowed diet to only chocolate peanut butter cups and water.
Vitamin C treatment	Vitamin C within 24 h of admission to the pediatric ICU
Effect of vitamin C administration	HD9: Repeat **ECHO** showed improvement in RV dilation and TR.<br><br>At follow-up 3 months, no signs or symptoms of cardiac

	dysfunction, and no evidence of PAH on repeat **ECHO**.<br><br>Sildenafil was discontinued 6 months after the cardiac arrest and a follow up **ECHO** 3 months off sildenafil remained stable: normal endsystolic septal position, normal RV size, and no RV hypertrophy.
Time series	Dr. Frank kindly provided us with the data for our time series graph, see below.
NOTES	We were able to contact Benjamin Frank, who responded 2023-2-9 to our question for the ECHO data:

Echo Timing/Parameters	Shortly After Arrest on ECMO	9 Days Post Arrest	~4 Weeks Post Arrest (Fig 1 B) on sildenafil	9 Months Post Arrest off sildenafil
Mean pulmonary artery pressure	42	25		
**Additional Parameters from Table 1 Below**				
Evidence of pericardial effusion (Y/N)	N	N	N	N
Right ventricular fractional area change	16.90%	33.80%		41.30%
Tricuspid annular plane systolic excursion	0.4	1.5		
Tricuspid valve regurgitation	3.3 m/s	2.6 m/s	2.3 m/s	2.3 m/s
Eccentricity	1.6	1.2	1.1	1.1
RV systolic to diastolic duration ratio	2.3	1.1	0.9	1

We were also able to contact Jaime Moore, who responded 2023-3-21 to our question about vitamin C doses and the vitals:

“1) Regarding the details of our patient's separate Vitamin C supplementation:
-Within 24h of the admission, he received an initial dose of enteral ascorbic acid 250mg crushed tablet delivered via nasogastric tube

-The following day, he received both a dose of enteral ascorbic acid 250mg crushed tablet via nasogastric tube and a dose of IV ascorbic acid 100mg

-For the next 6.5 days, he received IV ascorbic acid 100mg twice a day

-For the next 20 days, he received enteral ascorbic acid 125mg once a day

2) Baseline HR, BP, and respiratory rate:

It was documented on more than one occasion that because of the patient's (autism-related) behaviors, BP and HR were difficult or

	impossible to obtain during routine outpatient clinic visits.<br><br>On the date of his admission to the hospital, the first recorded full set of vitals were:<br><br>HR: 138<br>BP: 118/89<br>RR: 28<br>I would interpret these cautiously as this likely reflects some degree of patient anxiety and thus, inflation, compared to what they might be in an environment where he was calm/relaxed.”

**Ratanachu-Ek (2003a) [99]**	https://pubmed.ncbi.nlm.nih.gov/14700174<br>https://www.researchgate.net/publication/8938594
Patient	1 y 9 month boy. This is one of 28 cases reported, the next case is one from the same series.
Duration of the symptoms before hospital	No data
Signs and symptoms	Fever, limb pain and clinical right-sided heart failure [we infer that this indicates edema in this context].
Main findings	"Did not have any neurological signs of beriberi and diuretic effects from thiamine treatment" (p S738)<br><br>Pulmonary hypertension was diagnosed by **ECHO** (p S736)<br>"Moderate pericardial effusion"<br><br>"Two cases of pulmonary hypertension and right-sided heart failure diagnosed by echocardiogram without available investigation for vitamin B1 level. Both cases of pulmonary hypertension did not have any neurological signs of beriberi and diuretic effects from thiamin treatment. So the pulmonary hypertension might not relate to thiamin deficiency" (p S738).
Drugs in hospital	None
Other vitamins and minerals	Vitamin B1, but dose not mentioned
Diet before the event	"In the present study, the dietary history revealed that intakes of fresh vegetables and fruit or fruit juice were inadequate" (p S737).<br>"fed with well-cooked foods and small amounts or no vegetables and fruits" (p S734).<br>In all, there were 28 scurvy cases, but the above statement seems to apply also to the 2 cases we include in our study.
Vitamin C treatment	0.15-0.3 g/d vitamin C.<br><br>This is one of 28 cases, with description that "underweight cases were supplemented with multivitamins and iron was supplemented in cases suspected of iron deficiency anemia" but there is no specific description for this case.
Effect of vitamin C	"Clinical signs of limb pain and heart failure improved after a few days"<br>Normal **ECHO** was recorded 1 week after treatment (p S736).<br><br>"The result of vitamin C treatment in this study was dramatic with gradual improvement of all physical signs in a few days" (p S738).<br><br>"There was no previous report on pulmonary hypertension in scurvy but two cases in the present study responded to vitamin C treatment" (p S738).
NOTES	We were able to contact Suntaree Ratanachu-ek, who responded 2023-3-31:<br>"I did not have any recollection about 2 cases of pulmonary hypertension in 28 patients in 2003, and all previous records were destroyed."

**Ratanachu-Ek (2003b) [99]**	https://pubmed.ncbi.nlm.nih.gov/14700174<br>https://www.researchgate.net/publication/8938594
Patient	2 y 10 month boy. This is one of 28 cases, see previous case.
Duration of the symptoms before hospital	No data
Signs and symptoms	Fever, limb pain and clinical right-sided heart failure [we infer that this indicates edema in this context].
Main findings	“Did not have any neurological signs of beriberi and diuretic effects from thiamine treatment” (p S738)<br><br>Pulmonary hypertension was diagnosed by **ECHO** (p S736)<br>“Hypertension” but no data<br><br>“Two cases of pulmonary hypertension and right-sided heart failure diagnosed by echocardiogram without available investigation for vitamin B_1 level. Both cases of pulmonary hypertension did not have any neurological signs of beriberi and diuretic effects from thiamin treatment. So the pulmonary hypertension might not relate to thiamin deficiency” (p S738).
Drugs in hospital	None
Other vitamins and minerals	Vitamin B1, but dose not mentioned
Diet before the event	“In the present study, the dietary history revealed that intakes of fresh vegetables and fruit or fruit juice were inadequate” (p S737).<br>“fed with well-cooked foods and small amounts or no vegetables and fruits” (p S734).<br>In all, there were 28 scurvy cases, but the above statement seems to apply also to the 2 cases we include in our study.
Vitamin C treatment	0.15-0.3 g/d vitamin C .<br><br>This is one of 28 cases, with description that “underweight cases were supplemented with multivitamins and iron was supplemented in cases suspected of iron deficiency anemia” but there is no specific description for this case.
Effect of vitamin C	“Clinical signs of limb pain and heart failure improved after a few days”<br>Normal **ECHO** was recorded 1 week after treatment.<br><br>“The result of vitamin C treatment in this study was dramatic with gradual improvement of all physical signs in a few days” (p S738).<br><br>“There was no previous report on pulmonary hypertension in scurvy but two cases in the present study responded to vitamin C treatment” (p S738)<br><br>Hypertension was slowly improved in 2 weeks.
NOTES	We were able to contact Dr. Ratanachu-ek, who responded 2023-3-31:<br>“I did not have any recollection about 2 cases of pulmonary hypertension in 28 patients in 2003, and all previous records were destroyed.”

**Sakamornchai 1 (2022a) [100] Oraporn Dumrongwongsiri**	https://pubmed.ncbi.nlm.nih.gov/36337659
Patient	6y boy with ASD. The same report published 2 cases, see also the next.
Duration of the symptoms before hospital	2 months.
Signs and symptoms	Progressive bilateral knee swelling and refused to walk for 2 months.<br>Progressive dyspnea for 1 day.
Main findings	He was pale and had respiratory distress.<br>Left leg was swelling and limited movement due to pain.<br>Corkscrew hairs were presented over the body.<br><br>HR 130 bpm, BP 107/78 mmHg, respiratory rate 48/min.<br>SpO_2 90% on room air, corrected with 10 L/min oxygen via face mask.<br><br>**ECHO** showed TR with TRPG of 80 mmHg and D-shape LV.<br>He was diagnosed with severe PAH, but wedge pressure and PVR were not published.<br>"No evidence of other causes of PAH such as congenital heart disease, pulmonary embolism, and malignancy was found", but no further examinations are described to exclude them.<br><br>Hb 70 g/L<br>MCV 60 fL<br>Fe 14 µg/dL (normal 50-120)<br>Transferrin saturation 4.4% (>16%)<br>Ferritin 41.5 ng/mL (7-140)<br>25-OHD 16.2 ng/mL (20-100)<br>Folate 4.6 ng/mL (4-20)<br><br>Vitamin C level undetectable
Drugs in hospital	Inotropics<br>Pulmonary vasodilator (not specified)
Other vitamins and minerals	Multivitamins, vitamin D, Fe, folate and "oral nutritional supplement" were continued after discharge.
Diet before the event	Meals consisted of rice porridge and boiled eggs. Some biscuits were provided as a snack. Never drank milk for 2 y and refused red meat, fruit, and vegetables.
Vitamin C treatment	0.3 g/day of vitamin C was given via oral route since the 1st day of admission.
Effect of vitamin C	Gradually recovered from respiratory distress and could be discharged in 13 days.<br><br>1-month follow-up visit after discharge showed that he was able to mobilize both lower extremities without pain and had no dyspnea while doing the activity.

	**ECHO** at 2 months after discharge showed no evidence of PAH and the pulmonary vasodilator could be weaned off.
NOTES	To ask for further details of the case, we contacted Oraporn Dumrongwongsiri by email on 2023-2-8 and 2023-2-23 but did not get a response.

**Sakamornchai 2 (2022b) [100] Oraporn Dumrongwongsiri**	https://pubmed.ncbi.nlm.nih.gov/36337659
Patient	5 y boy with ASD, allergic rhinitis, and snoring.<br>The same report published 2 cases, see also the previous.
Duration of the symptoms before hospital	2 weeks
Signs and symptoms	Progressive dyspnea and refused to walk.
Main findings	Limited range of motion of joints due to pain.<br>No scorbutic rosary and no corkscrew hairs.<br><br>HR 136 bpm, BP 110/60 mmHg, respiratory rate 50/min.<br><br>Chest X-ray showed cardiomegaly, normal pulmonary blood flow and no pulmonary congestion.<br><br>ECG showed right axis deviation and low QRS voltage.<br><br>**ECHO** showed moderate to severe TR with TRPG 80 mmHg, RA and RV enlargement with D-shape LV, impaired RV systolic function, no evidence of intra-cardiac shunt.<br>"PAH was diagnosed", but wedge pressure and PVR were not published.<br><br>Hb 99 g/L<br>MCV 54.2 fL<br>Fe 20 µg/dL(normal 50-120)<br>Transferrin saturation 4% (>16%)<br>Ferritin 7.5 ng/mL (7-140)<br>25-OHD 6.02 ng/mL (20-100)<br>Vitamin B1 49.2 µg/L(28-85)<br>Folate 3.2 ng/mL (4-20)<br><br>Vitamin C level undetectable
Drugs in hospital	Pulmonary vasodilator (not specified)
Other vitamins and minerals	A single dose of 100 mg vitamin B1 intravenously.<br>10 l/Multivitamins, Fe, folate supplement were given and continued after discharge.
Diet before the event	Only rice porridge without any meat for 1 y, and he just started having some amount of boiled egg for 1 week. He usually drank 2 L/day of plain UHT milk. Refused fruit and vegetables and did not receive any vitamin or mineral supplementation.
Vitamin C treatment	0.3 g/day of vitamin C orally.
Effect of vitamin C	Symptoms improved dramatically the next day after vitamin C, and he was able to wean himself off respiratory support, and an **ECHO** revealed that his PH had improved.<br>Discharged on HD5.

	**ECHO** at 3 months after admission showed no PH.
NOTES	To ask for further details of the case, we contacted Oraporn Dumrongwongsiri by email on 2023-2-8 and 2023-2-23 but did not get a response.

**Shah (2021) [101]**	https://pubmed.ncbi.nlm.nih.gov/33859699
Patient	35 y female, obesity, substance abuse, 22-pack-yr smoking, major depressive disorder, anxiety
Duration of the symptoms before hospital	6 months
Signs and symptoms	Progressively worse shortness of breath, painful lower extremity swelling, decreased food intake, ecchymosis.<br>Persistent anemia.
Main findings	Diffuse joint swelling with ecchymosis, perifollicular petechiae with some corkscrew hairs, tenderness in the lower extremities.<br><br>Decreased breath sounds with mild crackles.<br><br>HR 105 bpm, BP 115/59 mmHg, respiratory rate 24/min, SpO_2 99% on room air.<br><br>A ventilation/perfusion scan excluded pulmonary embolism.<br><br>**ECHO** showed severe PH, with sPAP of 86 mmHg, dilated inferior vena cava with a RA pressure 15 mmHg, and severely enlarged RV with flattening of the interventricular septum with D-shape LV. LV was concentrically hypertrophic, with LVEF 70%. LV outflow tract septal obstruction from an enlarged RV.<br><br>Hb 66 g/L.<br>INR 1.5 (normal 0.87-1.17).<br>BNP 2714 pg/ml (<299).<br>Folate <2 ng/ml (normal >4.5)<br>Vitamin B1 <7 nM (8-30)<br>Urine test found benzodiazepines, morphine, codeine.<br><br>Vitamin C level undetectable, <0.1 mg/L.
Drugs in hospital	No PH drugs<br>Antibiotics
Other vitamins and minerals	
Diet before the event	Primarily cheese steaks, chicken, and pork chops.
Vitamin C treatment	Vitamin C 1 g/day orally.
Effect of vitamin C	Within 1 week, the patient reported improvement in her shortness of breath, lower extremity tenderness, and gingival bleeding.<br><br>After 2 weeks, repeat **ECHO** was negative for any indications of PH. RV size and RA pressure (3 mmHg) were normal. Right heart **catheterization** had a baseline mPAP 19 mmHg and PVR 1 Wood unit, wedge 10 mmHg.<br><br>"the patient's severe pulmonary hypertension resolved after 2 weeks of vitamin C supplementation."

	2 y later on cardiology assessment, there was no shortness of breath and no evidence of PH.
NOTES	We were able to contact Vandan Shah, who responded 2023-1-25 to our question about vitamin C dose in hospital as follows:<br><br>“Yes the same dose was used as an inpatient!”

**Singh (2017) [102]**	https://www.atsjournals.org/doi/abs/10.1164/ajrccm-conference.2017.195.1_MeetingAbstracts.A6187
Patient	48 y woman. Smoked marijuana, denied alcohol.<br>Was on prednisone for presumed pericarditis.
Duration of the symptoms before hospital	2 weeks
Signs and symptoms	Progressive dyspnea, chest pain, and 2 episodes of emesis.
Main findings	HR 120 bpm, BP 93/48 mmHg, SpO_2 97% on room air.<br><br>Cardiac auscultation found TR murmur.<br>Jugular venous pressure was elevated with loud P2, prominent S3.<br><br>Chest CT excluded pulmonary embolism.<br>Abdominal CT found pancreatitis.<br><br>**ECHO** showed severely dilated and dysfunctional RV [6 months earlier ECHO was normal].<br><br>Right heart **catheterization** showed severe PAH with significant vasoreactivity. mPAP 41 mmHg, RA pressure 16 mmHg<br><br>Vitamin C level undetectable.
Drugs in hospital	Trepostinil<br>Antibiotics<br>Stress dose steroids, but the above led to worsening of cardiogenic shock.<br>Vasopressin<br>Inotropics<br>Epoprostenol<br>Sildenafil on discharge, but was later tapered.
Other vitamins and minerals	
Diet before the event	Diet over past year consisted primarily of lettuce.
Vitamin C treatment	Vitamin C orally, but dose not reported.<br>In addition, sildenafil was prescribed on discharge, but that was tapered and she remained asymptomatic.<br>Therefore, we classify the treatment as vitamin C alone.
Effect of vitamin C	In 36 hours, significant clinical and hemodynamic improvement.<br><br>After 1 month, repeat **ECHO** showed normal RV size and function.
NOTES	

**Tan (2020) [103] Leong Ming Chern**	https://pubmed.ncbi.nlm.nih.gov/33300487
Patient	7 y boy with ASD.<br>Prior to this illness, he had no activity limitation and played on the trampoline daily.
Duration of the symptoms before hospital	No data
Signs and symptoms	Lethargy, anorexia, refusal to walk.
Main findings	He was planned for MRI brain and spine. However, after MRI brain, he developed unexplained sinus tachycardia and MRI spine was aborted.<br><br>BP 90/57 mmHg.<br>ECG no significant changes except tachycardia.<br>Cardiac enzymes mildly elevated, but levels not reported.<br><br>**ECHO** showed a hypertensive RV with a septal shift to the left and moderate TR with a peak gradient 75 mmHg consistent with PH.<br>Flattening of the interventricular septum<br><br>He underwent physiotherapy and was discharged with diuretics only to present again 2 days later with dyspnea and RHF.<br><br>2nd visit (2 days later)<br>Dyspnoea, breathless, RHF.<br>Bilateral ankle edema, hepatomegaly, tachycardia.<br><br>Failed to maintain saturation in room air. Taken to ICU for ventilation.<br><br>CT scan of the lungs showed right heart congestion. There was no pulmonary embolism or interstitial lung disease.<br><br>Vitamin C level undetectable, <5 µM.
Drugs in hospital	Diuretic on 1st visit, no improvement, and came back 2 days later<br>iNO, no improvement<br>phosphodiesterase 5 inhibitor, no improvement<br>endothelin-receptor antagonist, no improvement
Other vitamins and minerals	
Diet before the event	Very limited diet of only fish and soup daily. No vegetables and very selective with fruit intake.
Vitamin C treatment	Vitamin C supplementation 1 g/d orally, see NOTES.
Effect of vitamin C	In 10 days, PH improved significantly. Ventilator settings were gradually weaned and he was extubated. All organ functions improved and underwent physiotherapy. Discharged with vitamin C 1 g/day.<br><br>Over 3 months, regained full power of lower limbs and was back to his usual activities. A follow-up diagnostic catheterization revealed a normal

	mPAP of 14 mmHg and PVR index 1.18 Wood units × m^2.<br><br>PH medication was stopped and he was maintained on vitamin C supplementation.
Time series	Dr. Leong kindly provided us with the data for our time series graph, see below.
NOTES	We were able to contact Leong Ming Chern, who responded 2023-1-16 to our question about vitamin C route as follows:<br><br>"We gave oral vitamin C supplement 1000mg daily via the Ryle's tube. Under the National Institute of Health (NIH), the daily upper limit for vitamin C for 9-13 year olds is 1200 mg and we settled for 1000 mg daily.<br>Dr. MC Leong<br>Congenital Cardiologist"<br><br>See further data on the next page

On 2023-3-27 we received further data from Leong Ming Chern:

I have traced the notes and would like to reply your queries.

At the time of diagnosis:

Heart Rate: 118/min

Respiratory Rate : 36/min

You also show the echo figures for "post-vitamin C", But there are no pulmonary artery pressure (PAP) values for those other echos

We did repeat the echocardiogram one week after the child was admitted. We found that the peak TR gradient was 60mmHg but the RV function has significantly deteriorated. The RV was very likely too weak to generate a good TR.

Did you have more than 1 repeat echo?

Yes. We were monitoring his echo on a weekly basis and more during the acute stage.

Do you still have data for the echos, tricuspid regurgitation velocity or PAP values

The peak velocity of the TR 1 week later was 3.9m/s. The RV function: RV FAC : 11.9%; TAPSE : 13mm (but the heart was just rocking), presence of pericardial effusion.

Do you have available other measures such as TAPSE or eccentricity index?

Eccentricity : 1.4

3 months later catheterization. PVR index was 1.18 Wood units x m2. Can you find the estimate of body surface?

Weight : 32kg; Height : 137cm.

I did not perform a cardiac catheterization during the acute stage as the patient was very ill. I am sending you the echo image via wetransfer to allow you to have a better picture.

Dr. MC Leong

Congenital Cardiologist

On 2023-3-30 we received further data from Leong Ming Chern:
###################
I have extracted these data from our echocardiogram recordings.

Column1	15/10/2019	21/10/2019	31/10/2019	04/02/2020
Dominant RV	Yes	Yes	No	No
Peak TR gradient	75	60	40	12
LV eccentricity	2.05	1.4	1.02	1.01
RV FAC	18.7	11.9	52.7	51.1
Pericardial effusion	Yes	Yes	No	No

Thank you.

MC Leong

###################

**Ueki (2022) [104]**	https://pubmed.ncbi.nlm.nih.gov/35942728
Patient	11 y boy with ASD
Duration of the symptoms before hospital	3 months
Signs and symptoms	Fatigue, myalgia, arthralgia of the knees, difficulty ambulating.<br>Weight loss 4.4 kg in 5 months
Main findings	Dry skin, mild swelling of the knees, and limited range of movement at the knees and pelvis because of severe muscular pain.<br><br>CXR, ECG, and **ECHO** demonstrated RV volume overload (% of RV end diastolic volume index; 136.5) with mild PH and TRV 2.9 m/s<br><br>HR, BP, and SpO_2 saturation were within normal ranges.<br><br>Hb 68 g/L<br>MCV 67 fL<br>Fe 250 μg/L (normal 400–1880)<br>BNP 26.9 pg/mL (<18)<br>CRP 34 mg/L<br>D-dimer 3.77 mg/L (<1.0)<br>Vitamins B1, B12, folate, Se, Zn all within normal range<br><br>Vitamin C level undetectable, <0.2 μg/ml (<1 μM).
Drugs in hospital	None
Other vitamins and minerals	Oral Fe daily for 10 days. Thereafter “iron supplementation for 3.5 months” but doses were not reported.
Diet before the event	Selective diet, including pancakes, apple juice, milk, bananas, and boiled eggs at home. A very minimal amount of vegetables, meat, and fish from his school lunches and had never taken any nutritional supplements
Vitamin C treatment	“1 g intravenous vitamin C for 10 days. Thereafter, oral 0.3 g/day vitamin C for 3.5 months. Then vitamin C was stopped, but plasma level decreased below detection and thereafter 0.2 g/day was continued”.<br>See NOTES.
Effect of vitamin C	At discharge (following 10 days of intravenous vitamin C treatment), there was almost complete resolution of pain, and the patient was able to ambulate without assistance”<br>Abnormalities associated with pulmonary hypertension on ECG and ECHO improved following 3.5 months of oral vitamin C<br><br>Effects of vitamin C on laboratory values:<br><br>Hb<br>at diagnosis 68 g/L<br>At discharge: 97 (+43%)<br>4 months: 141 (+107%)<br><br>BNP<br>at diagnosis 26.9 pg/mL

	At discharge: 15.8 pg/mL (-41%)<br>4 months: 6.0 pg/mL (-78%)<br><br>CRP<br>at diagnosis 34 mg/L<br>At discharge: 2.8 mg/L (-92%)<br>4 months: 0.4 mg/L (-99%)<br><br>D-dimer<br>at diagnosis 3.77 mg/L<br>At discharge: 0.72 (-81%)<br>4 months: <0.25 (-93%)<br><br>IL-6<br>at diagnosis 38.08 pg/mL<br>At discharge: 11.08 (-71%)<br>4 months: 7.61 (-80%)<br><br>IL-8<br>at diagnosis 15.53 pg/mL<br>At discharge: 12.53 (-19%)<br>4 months: 201.67 (+1200%)<br><br>IL-18<br>at diagnosis 104.70 pg/mL<br>At discharge: 155.29 (+48%)<br>4 months: 201.94 (+93%)
NOTES	We were able to contact Masahiro Ueki and received the following reply on 2023-3-8.<br><br>"In our patient, he was treated with 1000mg of vitamin C intravenously for initial 10 days. Following resolution of muscle pain and inflammation, 300mg of vitamin C was administered orally.<br>At 3.5 months, abnormalities associated with pulmonary hyper-tension on ECG and UCG improved.<br>I stopped vitamin C supplement at 6 month, whereas his serum vitamin C level decreased below detection without any symptoms following 3 months. After this event, He continues 200 mg of vitamin C supplement."<br><br>We received the following addition on 2023-3-10.<br><br>"We evaluated PH by tricuspid valve regurgitation.<br>At diagnosis, velocity of Tr was 2.9 m/s.<br>At 3.5 month, velocity of Tr reduced to 2.5 m/s.<br><br>I'm sorry that TAPSE was measured only at 3.5 months.<br>TAPSE at 3.5 month was 21mm.<br><br>Right ventricular outflow tract diameter (RVOTD) was measured

	sequentially.<br>RVOTD at diagnosis was 23.6mm (he is suffered from mild dehydration), and 26mm at 3.5 month.<br><br>On 2023-3-23:<br><br>“Changes in vital signs were not significant during the treatment.<br><br>At diagnosis/ hospital admission<br>HR 85 BP 89/52 RR 18<br>At discharge<br>HR 97 BP 100/59 RR 18<br>At 3 months<br>HR 93 BP 107/59 RR 16”

**Valencia (2022) [105] Melody Duvall**	https://pubmed.ncbi.nlm.nih.gov/35969659
Patient	19 y male.<br>Crohn's disease, primary sclerosing cholangitis, autoimmune hepatitis, decompensated cirrhosis had progressed over 6 y to end-stage liver disease.<br>The patient underwent liver transplantation.<br>An ECHO performed at another hospital 1 y before transplantation showed RV pressure >½ systemic, normal biventricular size and systolic function, and normal valvular function.
Duration of the symptoms before hospital	
Signs and symptoms	Postoperative ECG normal.<br>24 h after transfer to the ward (postop day 2), the patient developed persistent sinus tachycardia (rate, 110–130 beats/min) and hypoxia with escalation of his oxygen support to 4 L/min of oxygen via nasal cannula.
Main findings	CXR demonstrated retrocardiac opacification, mild interstitial edema, trace pleural effusions.<br>Became progressively more tachycardic, tachypneic, and hypoxemic, requiring 5 L/min of nasal cannula oxygen to maintain SpO2 > 90%.<br>Hospital emergency call for pediatric ICU-team bedside assistance was activated on postop day 3. Upon arrival of the ICU team, the patient developed bradycardia, loss of consciousness, and pulselessness and was in pulseless electrical activity arrest.<br>ECMO team was emergently activated.<br><br>**Echo** obtained prior to completion of ECMO cannulation demonstrated mildly depressed LV function, severely depressed RV function, RV dilation, and RV hypertension (RV pressure 2/3 systemic; RV pressure ~55 mm Hg plus the RA v-wave, while noninvasive systolic blood pressure 80 mmHg). No pericardial effusion, no evidence of pulmonary embolism at the bifurcation of the main or proximal branch pulmonary arteries. TRV 4.84 m/s<br><br>After 220 min resuscitation, an adequate circulation was achieved.<br><br>Laboratory results prior to cardiac arrest<br>Hb 93 g/L<br>Platelet 65<br>postoperative day 2<br>Hb 79 g/L<br>Platelet 6<br><br><br>**Cardiac catheterization:**<br>mPAP 77 mmHg (right) and 75 mmHg (left) (in their Supplement)<br>PVR 536.6 dynes/s/cm^{-5} = 6.7 Wood units (in their Supplement)<br>Wedge pressure 30 mmHg (right) and 31 mmHg (left) (in their Supplement)

	On day 12 of ECMO, after 72 hours without any bleeding, he was given IV epoprostenol... On day 15 of ECMO, epoprostenol was discontinued. Other medical therapies included use of low-dose epinephrine and macitentan, at an enteral dose of 5 mg daily.<br><br>"At the time, we also considered the possibility of vitamin C deficiency as an associated causal factor in the development of severe PAH, since vitamin C deficiency is a complication of Crohn's disease, even in patients with disease remission. We did prescribe vitamin C in this patient: we used high dosing via parenteral nutrition and eventually enterally." (p e446)<br><br>The blood level of vitamin C was measured 7 weeks after the cardiac arrest event, and it was severely low (<5 µM).
Drugs in hospital	Epoprostenol<br>Milrinone<br>Sildenafil<br>Epinephrine<br>Macitentan
Other vitamins and minerals	
Diet before the event	Not described, and may be a secondary issue for this particular patient
Vitamin C treatment	Vitamin C initially intravenously and thereafter orally. Day of starting is not described, and doses not described (only "high dosing via parenteral nutrition and eventually enterally")
Effect of vitamin C	"The patient underwent ECMO decannulation after 29 days and was solely supported by the paracorporeal lung-assist device. It should be noted that prior to removing the patient from ECMO, aggressive, serial bronchoscopies were required to ensure patent airways for gas exchange. Additionally, a PA catheter was placed for evaluation of readiness for decannulation from the paracorporeal lung-assist device. PA pressures ranged from 35/25 to 45/30 mm Hg (mPAP, 30–35 mm Hg) (<½ systemic)"<br><br>He was weaned from sildenafil 5 months later and from macitentan 12 months later. Follow-up echocardiogram at 21-month post-transplant demonstrated low RV pressure and qualitatively normal RV function. His follow-up vitamin C level at the time of pulmonary hypertension resolution had normalized.
NOTES	The report is confusing.<br><br>"The blood level of vitamin C was measured 7 weeks after the cardiac arrest event" (p e446), which corresponds to 49 days.

	On the other hand, "The patient underwent ECMO decannulation after 29 days and was solely supported by the paracorporeal lung-assist device… decannulated from the paracorporeal lung-assist device after 15 days (3-d post-ECMO decannulation). TTE thereafter demonstrated mild RV hypertension (<½ systemic) and normal biventricular systolic function."<br><br>It is not clear how the 49 day vitamin C measurement relates to the 32 day (=29+3) decannulation from paracorporeal lung assist device.<br><br>The time of starting vitamin C is not described.<br>Therefore the role of vitamin C in the recovery from PH remains obscure in the report.<br><br>To ask for further details of the case, we contacted Melody Duvall by email on 2023-2-8 and 2023-2-23 and 2023-3-4 but did not get a response.
